# Veterinary peer study groups as a method of continuous education—A new approach to identify and address factors associated with antimicrobial prescribing

**DOI:** 10.1371/journal.pone.0222497

**Published:** 2019-09-19

**Authors:** Valerie-Beau Pucken, Gertraud Schüpbach-Regula, Manuela Gerber, Corina Salis Gross, Michèle Bodmer

**Affiliations:** 1 Clinic for Ruminants, Vetsuisse Faculty, University of Berne, Berne, Switzerland; 2 Veterinary Public Health Institute, Vetsuisse Faculty, University of Berne, Liebefeld, Switzerland; 3 Swiss Research Institute for Public Health and Addiction, University of Zurich, Zurich, Switzerland; University of Illinois, UNITED STATES

## Abstract

Within the dairy industry, most antimicrobials are used for dry-cow therapy or mastitis treatment. To reduce antimicrobial usage in dairy cows, increasing awareness and behaviour change is necessary. As veterinarians are known to be influenced by their peers, peer study groups as a continuous education might contribute to this. Therefore, the objective of this study was to analyse written records of veterinary peer study group meetings to identify factors associated with antimicrobial prescribing decisions, and to analyse veterinarians’ attitude towards the benefits of this continuous education method. Twenty-three participating Swiss cattle practitioners were divided into three groups. Each group met every two to five months, together with a facilitator and an expert on the topic to be discussed. Written records from every meeting were taken and analysed qualitatively to identify factors influencing veterinarians’ decisions on antimicrobial prescribing and mastitis therapy. In addition, focus group discussions were conducted after the last meeting, to assess the veterinarians' learning achievements gained during the peer study group meetings. Extrinsic factors such as external pressure, competition, farmer, individual animal, farm and diagnostics as well as intrinsic factors such as own experience/attitude, knowledge and change of mindset during career could be shown to influence veterinarians’ decisions on antimicrobial prescribing. In the focus group discussions, the veterinarians stated that they gained new knowledge, received new stimuli, exchanged with their peers and felt supported in their relationship to their farmers. Since the identified factors are partly interrelated, it is not sufficient to change a single factor to achieve a change in the antimicrobial prescription behaviour of veterinarians. Veterinary peer study groups could contribute to the intention to change, because veterinarians experienced multiple benefits from this method of continuous education. In order to quantify this, the prescription data of the veterinarians are analysed in a next step.

## Introduction

Antimicrobial usage (AMU) in food producing animals is known to contribute to the emergence of antimicrobial resistance [1–3]. As a result, several countries aim for the reduction of AMU by the introduction of a surveillance of antimicrobial usage with a special focus on food producing animals, which includes dairy cows [4–6]. In Switzerland, the *National Strategy on Antibiotic Resistance (StAR)* was launched in 2015 [7], and the *Veterinary Medicinal Products Ordinance* was changed in April 2016 to aim for an evidence-based use of antimicrobials (AMs) and restrict antimicrobial drug dispensing [8]. Even before that, veterinary drugs belonging to the category of antimicrobials could only be prescribed by a veterinarian [9]. The change in the *Veterinary Medicinal Products Ordinance* made this even more stringent. Now veterinarians may no longer leave stockpiles (or stores) of AMs intended for the prophylactic treatment of farm animals or "highest priority critically important antimicrobials" (HPCIAs) on farms. They are only allowed to dispense quantities of HPCIAs sufficient to treat those animals they have examined [8].

Within the dairy sector most AMs are used for treatment of mastitis and dry cow therapy [10–13]. Therefore, the focus of antimicrobial reduction is on the improvement of udder health, evidence-based treatment of mastitis and selective dry cow therapy [14–16]. Switzerland has a large dairy sector and is the country with the highest sale of intramammary antimicrobial preparations per population correction unit (biomass of livestock and slaughtered animals) in 2015 and 2016, in comparison to 29 other European countries [17–21].

A reduction of AMU in the dairy sector might be achieved by the implementation of a herd health management (HHM) plan that focuses on the prevention of infection and evidence-based use of AMs [16,22]. In this process the farm veterinarian, as one of the farmers' most important contact person for udder health and mastitis treatment, has a key role [23]. As the veterinarian can influence farmers' use of AMs to a high degree [24,25], a change in behaviour to more evidence-based medicine is also necessary among veterinarians for the successful reduction of AMs. Continuing veterinary medical education (CVME) as continuous education (CE, continuing professional development (CPD)) can provide the motivation, education and support to achieve this, and might therefore be a promising approach [26–29]. CE with general practitioners for human patients have already shown to improve evidence based medicine skills and to reduce antimicrobial prescribing [30,31].

CE could be provided via peer study groups, which are consistent groups, meeting on a regular basis and aiming for professional improvement (e.g. critical discussion of personal practice) [32]. It has been shown that peer study groups are common among general practitioners for human patients in Switzerland [32] and are also appreciated among farmers, enabling successful knowledge transfer [23,33]. Moreover, it was shown that peer study group participation of farmers could lead to a reduction of AMU without deterioration of udder health [34,35]. Advice and opinions from colleagues are important for decision making processes not only for farmers but also for veterinarians [36,37]. Nevertheless, at present, the majority of CVME in Switzerland consists of lecture-like events, with a large and/or variable number of participants. Peer group education has not yet been used as CVME among veterinarians in Switzerland. In veterinary medicine students, the positive aspects of peer study group learning were already investigated [38–40]. The benefits of veterinary peer study groups (VPSGs) for CVME have not yet been investigated in detail.

Considering the important role of veterinarians in the reduction of AMs, it is important to get insight into veterinarians' mindset towards the antimicrobial prescription practice for mastitis treatment [23,41]. Factors affecting the prescription patterns of veterinarians and the administration patterns of farmers have already been analysed several times in recent years [27,28,42–47]. Within those studies, data were collected qualitatively (interviews, questionnaires and focus group discussions) and quantitatively (antimicrobial data collected through veterinary prescription and dispensing records and sales data). Up to now, VPSG discussions have never been used as a data source to identify the factors influencing antimicrobial prescription for udder health.

Therefore, the first objective of this study was to identify factors influencing veterinarians' decisions to prescribe AMs, using written records from peer study group meetings. The second aim was to obtain veterinarians' view on this method of CVME in terms of their perceived learning experience.

## Materials and methods

### Selection of participants

Swiss cattle practitioners were recruited for participation in regular peer study group meetings with the headline topic udder health.

For this purpose, two newsletters were sent out via the *Cattle Health Service (RGD)* and one article was published in the *Swiss Archive for Veterinary Medicine* [48]. Additionally, the project was presented at the CVME for farm animal practitioners in Berne and Zurich, which are organized by the Vetsuisse Faculties of the Universities of Berne and Zurich. This monthly lecture-like event presents case studies and projects from the faculties and is open to every interested veterinarian. The participating practitioners were required to fulfil the following two preconditions. Firstly, they should be a member of the Swiss Association for Ruminant Health and secondly, they should work with a specialized practice software that facilitates the acquisition of antimicrobial prescription data. These data will be used in further studies to analyse the amount of AMs prescribed during the peer study group period.

In total 30 interested veterinary practices replied. Each practice represented at least one veterinarian. Eight veterinary practices decided against participation due to time constraints, and one veterinary practice was too far away to contribute to the peer study groups on a regular basis. As a result, 21 participating veterinary practices were subdivided into three peer study groups. From these 21 veterinary practices 23 veterinarians, from approximately 500 Swiss cattle practitioners, participated [49,50]. The VPSG were built according to mother tongue and for organisational reasons, as the veterinarians had to be able to reach the venue of the meetings within an hour. Seven veterinarians were French-speaking and formed the peer study group in the Romandie, the French-speaking part of Switzerland. Eight veterinarians came from Berne and its surroundings and formed the peer study group in Berne. The remaining eight veterinarians came from the northern and eastern part of Switzerland and formed the peer study group in eastern Switzerland. The geographical distribution of the participating veterinarians largely corresponds to the nationwide distribution of dairy farms and thus also to the distribution of cattle practitioners [51,52]. However, the study population is too small to be considered representative for all Swiss cattle practitioners.

The participants were aware that the VPSGs were part of a project which focuses on antimicrobial reduction for udder health. They received an information sheet on the purpose of the study and signed an informed consent, assuring confidential handling of the data (S1 Appendix). The veterinarians did not receive any compensation payment for the participation and the only incentives they received were snacks during the evenings and credit points for continuous education granted by the Swiss Association for Ruminant Health.

### Peer study group meetings

Each peer study group met every two to five months (depending on the needs of participants) for one evening, starting in August 2016 and ending in February 2018. During the initial kick-off meeting, the veterinarians decided firstly on the topics of the peer study group meetings and secondly on the sequence of topics for the next three meetings. The sequence of the remaining topics evolved during the following peer study group meetings. During every meeting a facilitator (the first author, VP) and an expert, which was always a person with expertise in the respective topic, was present. To train as a facilitator, VP attended a farmers’ peer study group and twice met the head of consulting of INFORAMA Rütti, an educational, advisory and convention centre for agriculture and home economics that offers peer study groups for farmers. She did not know the veterinarians personally in advance. Several times the senior author (MB) acted as the expert of the evening.

The evenings were designed so that veterinarians could share their assessments and experiences on the subject. For this purpose, the facilitator asked questions that were always adapted to the respective topic. For the questions on the assessment within the topic, the veterinarians were supposed to classify themselves with the help of a scale. However, there was also room for an open discussion. The questions on the veterinarians' experiences aimed to encourage an open discussion on the specific issue. All these questions were essentially the same for each VPSG. Occasionally questions were added, which resulted from experiences that had been gained at previous meetings on this topic. In addition, there was a discussion round with the expert. This discussion round was either an open discussion with the expert and the veterinarians or the expert gave a presentation about the respective topic during which the veterinarians could ask questions throughout. The evenings always ended with a final feedback session among the participants. During the feedback session, the veterinarians were asked to give positive and negative feedback of the evening and which main conclusion they drew from the subject.

To offset variations in the culture of discussion between the peer study group members, the facilitator alternated between asking the veterinarians for oral or written contributions. Written contributions were collected by the facilitator and either read out or written on the blackboard. The written contributions ensured that each participant had the opportunity to express or share his or her opinion.

The facilitator’s main task was to prevent the discussion changing to other topics during the evening and to ensure that all participants were given equal time and opportunity to speak. Written records were taken from every peer study group meeting by one note-taker, which was one additional person. The initial kick-off meeting was excluded from analysis, as it was only explanatory and organisational.

### Data collection and analysis

The written records, taken from one note-taker, were digitalized and imported into the qualitative software MAXQDA Analytics Pro 12 (VERBI Software, Berlin, Germany). The quotations of the veterinarians were anonymized in order to make a direct allocation impossible. These anonymized written records were applied as datasets to identify factors that are influencing veterinarians' decisions on antimicrobial prescription and mastitis therapy (S2 Appendix).

### Framework method

**Fig 1:**
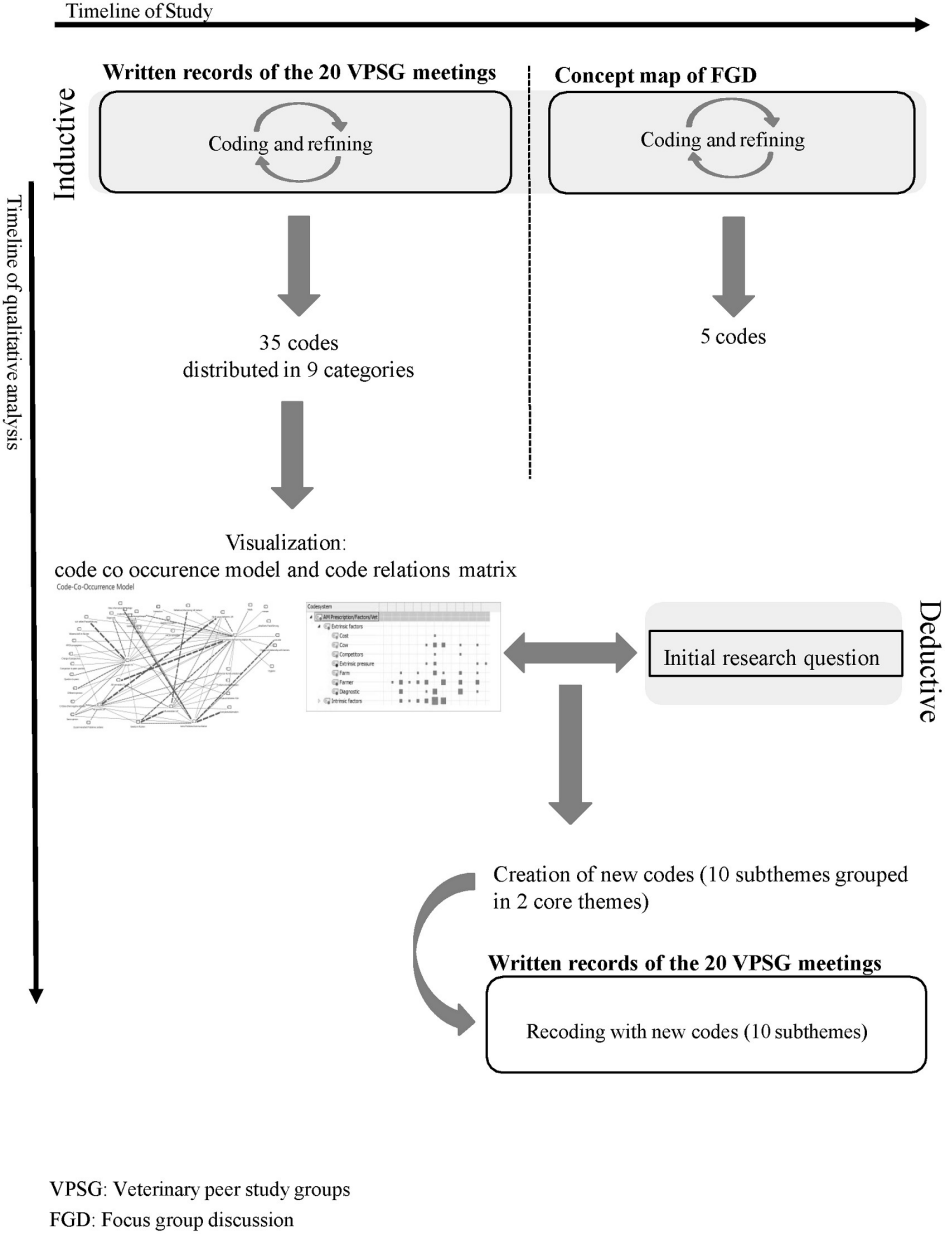
Flowchart as simplified illustration of the qualitative data analysis.

All written records were analysed using a modification of the thematic framework analysis from Gale et al. 2013 to identify factors which influence veterinarians' decisions on antimicrobial prescription and mastitis therapy (Fig 1) [53].

Firstly, for familiarization with the data, the written records from every peer study group meeting were read by two researchers (First author (VP, a veterinarian with training in qualitative health research methods) and third author (MG, a veterinarian working on a research project on reduction of AMU on farms)).

Afterwards, an inductive open coding was done independently by each researcher on three written records of each peer study group. A working analytical framework was developed by discussing the codes, dividing broad codes into more specific ones and identifying new codes. This initial framework was revised to incorporate refined codes. The refining and applying was repeated until no new codes were generated. In the end, 35 codes were selected to address the research question. These were summarized into nine categories reflecting the prominent subjects within the dataset. With MAXQDA Analytics Pro 12 the 35 codes were charted in a code-co-occurrence model (S1 Fig). With the code-co-occurrence model the overlap or common occurrence of codes in one segment are visualized in a map. In this map, the codes that overlap or co-occur are connected with a black line. The data were additionally charted in a code-relations-browser. This illustrates how many document segments contain the same combination of two codes. All possible combinations of the codes were taken into account. By reviewing the charted data, and considering the initial research question, two core themes and nine subthemes were identified as factors that influence veterinarians’ prescription practice of AMs for udder health (Fig 1).

Researchers responsible for data analysis met repeatedly during the whole data analysis to enable further exploration of participant responses and agreement on recurring themes.

### Focus group discussion

At the end of the study, all participants met for a final meeting. A focus group discussion (FGD) was conducted with each VPSG. During this FGD questions were asked in order to better evaluate the VPSG. Three questions focused mainly on the perceived learning experience of the veterinarians. These questions focused on therapy, diagnostics and the relationship with the farmer. One question was specifically tailored to the veterinarians' own perception of a change in their antimicrobial prescription (Table 1).

**Table 1:** Questions asked during the focus group discussion on the benefit of the study.

Questions on the benefit of the study:
1. To what extent has this VPSG helped you with the topic of diagnostics?
2. To what extent has this VPSG helped you with the topic of therapy?
3. To what extent has this VPSG helped you regarding relationships with farmers?
4. To what extent has this VPSG changed your prescription practices for AMs?

VPSG: Veterinary peer study groups

AM: Antimicrobials

In addition, the veterinarians were asked whether they would participate again in a VPSG. In order to collect and evaluate the answers of the participants to the questions on the benefit of the VPSG, concept maps were used [54]. For this purpose, every question was posted on the blackboard. A facilitator collected the answers from the veterinarians, which were then written onto the blackboard by a note-taker. The use of concept maps for data collection aims at a complete data representation. It is not relevant which dominant opinions are prevalent in the group [54]. The concept maps were digitalized and imported into the qualitative software MAXQDA Analytics Pro 12. The responses of the participants were inductively coded. The resulting codes were optimized and shortened until finally all answers of the participants could be reflected in five codes (Fig 1).

### Ethics statement

According to the Ethics Committee for Research of the Canton of Berne (decision: Req-2018-00020) and the Swiss legislation (Human Research Act), this study does not require ethical approval, as neither human diseases nor the structure and function of the human body were part of this study. In order to guarantee anonymity and to prevent any damage to the participants' reputation, the participants were anonymised. The informed consent signed by each participant can be found in the supporting information (S1 Appendix).

## Results

### Peer study groups

Due to the intervals between the VPSG meetings and limited study time, each of the groups met seven to eight times. They discussed six to seven topics, which were proposed by the participating veterinarians. One VPSG discussed the same topic during two meetings (Table 2).

**Table 2:** Order and topics of the VPSG meetings.

Meeting:	VPSG Berne	VPSG Eastern Switzerland	VPSG Romandie
1	Initial kick-off meeting	Initial kick-off meeting	Initial kick-off meeting
2	Diagnostics	Diagnostics	Drying off
3	Treatment of mastitis	Drying off I	Treatment of mastitis
4	Communication	Drying off II	Diagnostics
5	Complementary medicine	Treatment of chronic mastitis	Computer aided HHM
6	HHM in relation to mastitis	Treatment of acute mastitis	Feeding
7	Milking and technique	HHM in relation to mastitis	Milking and technique
8	Drying off	Milking and technique	

VPSG: Veterinary peer study group

HHM: Herd health management

Twelve out of the 23 participating veterinarians were women and 19 participants were practice owners. Age and years in practice were approximately evenly distributed among all participants, ranging from 32 to 69 years of age and five to 42 years in practice. However, there were large differences in the distribution of age, gender and number of years in practice between the individual VPSG (Table 3).

**Table 3:** Demographic data of the participating veterinarians, distributed per VPSG.

Category	Subcategory	VPSG Berne (n = 8)		VPSG Eastern Switzerland (n = 8)		VPSG Romandie (n = 7)		Total (n = 23)	
		Number	%	Number	%	Number	%	Number	%
Gender	male	2	25.0	3	37.5	6	85.7	11	47.8
	female	6	75.0	5	62.5	1	14.3	12	52.2
Age	30–39	1	12.5	3	37.5	4	57.1	8	34.8
	40–49	3	37.5	1	12.5	2	28.6	6	26.1
	50–59	4	50.0	2	25	0	0.0	6	26.1
	≥ 60	0	0.0	2	25	1	14.3	3	13.0
Years in practice	5–10	1	12.5	3	37.5	3	42.8	7	30.4
	11–20	2	25.0	1	12.5	2	28.6	5	21.8
	21–30	3	37.5	2	25	1	14.3	6	26.1
	≥ 31	2	25.0	2	25	1	14.3	5	21.7
(Personal) Veterinary activity in bovine sector	< 50	1	12.5	1	12.5	0	0.0	2	8.7
	≥ 50	7	87.5	7	87.5	7	100.0	21	91.3
Practice owner	Yes	6	75.0	7	87.5	6	85.7	19	82.6
	No	2	25.0	1	12.5	1	14.3	4	17.4

VPSG: Veterinary peer study group

In comparison to the total population of Swiss veterinarians, the age and gender distribution of our participating veterinarians was approximately representative. However, more practice owners participated in our study and there were no veterinarians with less than five years of experience [55].

Overall, there was a good attendance of the veterinarians in all VPSG meetings (S1 Table).

The VPSG meetings lasted around 2–3 h, depending on the number of participants, the expert, the topic and whether the participants were communicative, which differed between the groups. At the end of the last VPSG meeting, every group requested to continue the group meetings with different topics to be covered.

### Factors associated with antimicrobial prescription

**Fig 2:**
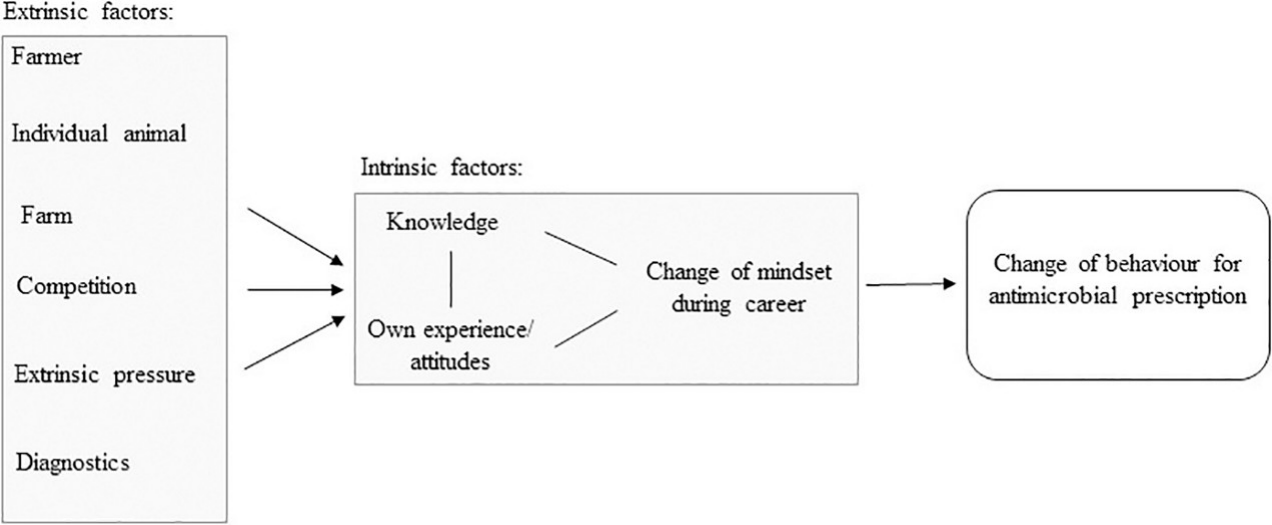
Factors associated with veterinarians’ antimicrobial prescription practice.

It was possible to determine nine subthemes as factors that were experienced to influence veterinarians in their prescription behaviour. These subthemes were grouped into two core themes, firstly extrinsic factors, which are associated with external pressure, competition and the individual case (farmer, individual animal, farm, diagnostics) and secondly, intrinsic factors, which are related to the veterinarians themselves. These factors were "own experience/attitude", "knowledge" and "change of mindset during career" (Fig 2).

#### Extrinsic factors

This core theme includes all factors that are not directly linked to the veterinarians themselves.

*Farmer*:

During the group meetings, the veterinarians stated that the farmer has an influence on their antimicrobial prescription. Particularly different mentalities, persisting behaviour, age and knowledge of farmers were mentioned as influencing on how veterinarians managed udder health and antimicrobial prescription.

V16: “*Farmers with less interest in udder health management are typically the ones that give up their profession*.” V10: “*Young farmers are more open minded* (in regards to udder health management).”

V9: *"The past veterinarian treated all animals with one specific antimicrobial agent*, *after his retirement*, *it was very difficult to change this practice amongst the farmers*.”

V18: *"With the therapy of Staphylococcus aureus there are two possibilities*, *either one treats the cows*, *or one removes them*. *(…) The treatment is expensive*, *but the farmer has then made the decision and preferred the treatment*. ”

*Individual animal*:

In addition, the age of the individual animal and the general health condition are factors that were mentioned as influencing the veterinarians’ decision on how and if to treat this individual animal.

V6: *"With a freshly calved cow one can wait at least six weeks* (before the decision has to be made whether or not a treatment against coagulase-negative staphylococci should be carried out), *and use dry off treatment sooner* (for cows in late lactation).”

In conjunction with the individual animal the farmer and diagnostics were also mentioned as having an impact on further decisions.

V17: *"I use an antibiogram before treatment*. *(…)*. *But the treatment decision always depends on the animal as well (young*, *old*, *etc*.*)*.”

V15: *"In the past fever used to be regarded as life fire*, *as energy*. *This means the inflammation has an important healing aspect*. *(…) Whether the cow can be left with having fever and without antimicrobial therapy depends on the farmer and the cow*, *the fever level does not matter*. *I would propose injecting anti-inflammatory medication*, *because of the pain and the inflammation*, *not because of the fever*, *and take a milk sample*. *The farmer should be able to assess it well*, *and you have to make new decisions on a case-by-case basis*.”

*Farm*:

The farm itself was mentioned together with the farmer as factors influencing the antimicrobial prescription.

Expert: *"V5*, *you mentioned a problem with a farm that had a milking robot*?”

V5: *"Yes*, *they didn`t have enough places at the feeding fences and the farmer only wanted to analyse milk samples from half of the herd*. *We then used the somatic cell count as a reference*, *we fixed the stray voltage problem in the milking robot and treated the worst cases*. *But the end result was not satisfying*.”

*Diagnostics*:

Another aspect that affects the veterinarians in their decision of antimicrobial prescription are diagnostic tools, upon which the veterinarians base their treatment decision. During the group meetings, different diagnostic tools were mentioned such as the California Mastitis Test (CMT), mastitis pathogen detection test kits, polymerase chain reaction (PCR), culture and susceptibility screening. The last two methods were either undertaken in the veterinary practices or performed by professional laboratories. Statements from the veterinarians about diagnostic tools, related to a decision-making process for antimicrobial treatment, were often made in conjunction with the individual animal or the farmer as influencing factors.

V1: *"Therapy* (of *Staphylococcus aureus*) *should only be initiated following an antibiogram and depending on the genotype* (Genotype might indicate a livestock problem). *If there is a herd problem changes need to be made in the milking order and the farmer needs to collaborate*.”

*Competition*:

Everything the veterinarians perceived as competitive in the prescription of antimicrobials was summarized in our study by the factor "competition". This was the competition from other colleagues but also that farmers sometimes buy cheaper antimicrobials abroad.

V17: *"Farmers can independently order cheap antimicrobial drying agents from France*. *This becomes problematic because it cannot be controlled*. *Farmers then have two separate refrigerators*.”

V14: *"In the region of my veterinary practice*, *there is still someone* (another veterinarian) *that leaves stockpiles of antimicrobials*. *This is one of the main problems that arises when talking with the farmers from my region*.”

In addition, the veterinarians indicated that they perceived other non-veterinary farm advisors as competitors. These competitors were, for example, consultants offering homeopathy. Since some farmers tend to involve these consultants rather than their farm veterinarian in udder health problems and mastitis treatment they might compete in the sales of AMs. However, they also mentioned feed consultants or milking technicians with whom the veterinarians compete for advising their farmers.

*Extrinsic pressure*:

The *Veterinary Medicinal Products Ordinance* changed in the spring of 2016 regulating the prescription of antimicrobials even more strictly [8]. The veterinarians stated this change of law as an impulse that changed their antimicrobial prescription and could have an impact on the cooperation between farmers and veterinarians.

V6: *"Before* (the change of the *Veterinary Medicinal Products Ordinance*) *we already used selective dry cow treatment*. *Now* (after the implementation of the *Veterinary Medicinal Products Ordinance*) *we are more precise with our selective dry cow treatments*.”

V10:*"The farmer asks* (about HHM) *or the veterinarian suggests it* (visits for the purpose of HHM). *It is interesting how farmers have different tolerance levels* (acceptance and discussion of potential problems on the farm). *The extra on-farm visits that were undertaken* (because of the change of the *Veterinary Medicinal Products Ordinance*) *were used as a chance to discuss with the farmer* (about AMU on farm). *Thanks to these extra discussions*, *udder health was improved*. *Farmers are therefore more willing to accept that not everything needs to be treated with antimicrobials*.”

#### Intrinsic factors

The second core theme contains the intrinsic factors. The intrinsic factors are centred in Fig 2, since in Switzerland the veterinarian is the person who is authorized to prescribe AMs [56]. Three intrinsic factors, "own experience/attitude", "change of mindset during career" and "knowledge" could be identified. These factors interact and are not independent of each other. In Fig 2 this is indicated by the black lines.

*Knowledge*:

The decision of veterinarians to prescribe AMs or not is based on their knowledge. This knowledge is derived from their education in pharmacology, immunology, physiology, and knowledge they have gained during their working life through CVME or reading scientific articles.

This knowledge was shared between the group members and might have an influence on the future antimicrobial prescription practice of the veterinarians.

V3: *"From a pharmacological standpoint it is not bad to use certain antimicrobials at the same time*, *they act the same way*, *even though there are different opinions about it*.”

(Pharmacodynamics and usage of certain AMs at the same time)

V23: *"I would probably do* (as a somatic cell count threshold level for use of antimicrobial drying agents) *what scientific articles suggest*, *which means 50'000 for primiparous and 150’000 for multiparous*.”

*Own experience/attitude*:

During the peer study group meetings, nearly every veterinarian mentioned that the decisions made were based on his or her own experience. Their own observations that were made during working life played an important role in the decision-making process.

V4: *"For very many years I only used anti-inflammatory drugs* (for the treatment of coliform bacteria) *and I had positive experiences*.”

V15: *"Experience has shown me that when I am sure and convinced about the therapy I am using*, *then it works better*.”

Every veterinarian’s own perceptions enter into the decision of antimicrobial prescription. However, this aspect is also related to and dependent on other factors, including extrinsic factors, as shown by the next quotation.

V17: “*Every veterinarian is free to work how he or she wants*, *taking into account their own values and methods*. *They are responsible for their actions*. *However*, *the client is always to be considered as the king*, *hence if we don`t want to lose them we must adjust to his or her needs*.”

*Change of mindset during career*:

There were some quotes that show a change of mindset among the veterinarians that participated in the peer study groups. This change was always related to a previous trigger, for instance because the law was changed, or because this topic was discussed in the VPSG. Since a change of mindset can lead to a change of intention, this factor is closer to behaviour change (Fig 2) [57].

V11: *"For acute but not quite so severe mastitis* (caused by *Escherichia coli*), *I will try to use only anti-inflammatory drugs* (no AMs). *At some farms I will try to have the courage to address and perform this*.”

(End of VPSG meeting on the topic “Therapy of acute mastitis”.)

V18: *"With regards to legal protection* (implementation of the new *Veterinary Medicinal Products Ordinance*), *we need to write down everything we do and prescribe* (patient history details and every antimicrobial usage and dispense). *There are no strict procedures that need to be followed*, *everybody can decide on their own*, *while having the reduction in the overall antimicrobial usage*!”

### Focus group discussion

Unfortunately, not all veterinarians were present at the FGD. From the VPSG Berne six veterinarians (75%) could participate and from the VPSG Romandie four veterinarians (57%) were present. From the VPSG in eastern Switzerland, five veterinarians have already announced in advance that they are not able to attend this meeting and two veterinarians had to cancel at short-term due to an emergency, therefore only one person (13%) from this VPSG was present. Altogether 11 veterinarians (48%), from 11 veterinary practices took part in the FGD. The answers of the participants to the questions on the benefits of the VPSG could be summarized in five codes (Table 4). The number of checkmarks does not represent the number of responses, since several responses could be assigned to a single code.

**Table 4:** Coded answers to the questions of the focus group discussion.

To what extent has this VPSG….	Support / reinforcement	Continue with old behaviour	New information/ knowledge	Comparison to peers	New stimuli
… helped you with the topic of diagnostics?			✔	✔	✔
… helped you with the topic of therapy?		✔	✔	✔	✔
… helped you regarding relationships with farmers?	✔			✔	
… changed your prescription practices for AMs?		✔			✔

✔: A checkmark indicates that this code could be coded for the answers to this question.

VPSG: Veterinary peer study groups

AMs: Antimicrobials

For all four questions there was a diverse feedback of the veterinarians whereby not every question could be coded with the same codes. The fact that the veterinarians could exchange experiences with their peers and found this helpful could be reflected in the code "comparison to peers". This was given for the topics therapy, diagnostics and the relationship to the farmer. A rethinking of the veterinarians is reflected with the code "new stimuli". This was indicated on the questions considering the topics of diagnostics, therapy and prescription practice of AMs. The most varied answers were given by the veterinarians to the question of whether the VPSG had helped them with the topic of therapy. Here the codes "continue with old behaviour", "new information/ knowledge", "comparison to peers" and "new stimuli" could be coded.

All veterinarians indicated that they would certainly participate a second time in a VPSG.

## Discussion

The good participation, the predominantly positive feedback and the request of the veterinarians to continue with the peer study group meetings demonstrated a general positive perception of this type of continuous education. Furthermore, we applied the framework method on the written records of the VPSG meetings. As a consequence, we could identify factors that possibly influence veterinarians’ antimicrobial prescription behaviour. The feedback given during the FGD showed that the veterinarians not only gained new knowledge through the VPSG, they also felt supported and received new stimuli to reflect on their present working practices. Although there were huge differences in the distribution of age, gender and number of years in practice between the individual VPSGs these differences were neither reflected in the factors nor in the feedback.

### Factors associated with antimicrobial prescription

In line with other studies, we found several factors (intrinsic and extrinsic) involved in the antimicrobial prescription behaviour of veterinarians [27,28,37,42–44,46]. These factors influence each other and are not independent, as shown by the quotations that include several factors within one sentence. An interrelation between the different factors related to antimicrobial prescription has already been shown by Speksnijder et al. 2015 [43].

Contrary to other studies [42,46,58], cost could not be identified as a single factor. In fact, costs were only mentioned once in connection with AMs in a quotation in which the factor "farmer" was predominant. This might be due to the fact that the participating veterinarians were mainly working in the dairy sector. This was also stated in a study by Speksnijder et al. 2015, in which all veterinarians, except dairy veterinarians, reported costs as a relevant factor for antimicrobial prescribing [43]. Another possibility would be that costs for AMs play a minor role for Swiss farmers, and thus also for Swiss veterinarians [59]. This could be explained by the small herd sizes and high levels of subsidies for Swiss agriculture [60].

There was no difference between the VPSGs in the identified factors or the interrelationships between the factors. However, there were regional differences in the factor "competition" regarding what or who was perceived as competition. The problem of farmers illegally buying AMs in European Union countries was only reported by veterinarians living near the border. In addition to the competition problem that arises for these veterinarians, this practice is also illegal and would have to entail legal consequences [9]. A consistent policy within the European countries could presumably prevent these illegal imports [43].

The factor "change of mindset during career" has a key role. Although it could be attributed to the intrinsic factors, it is closely related to the intention to change [57]. Furthermore, in our study there has always been a previous trigger related to this factor. These triggers were e.g. the recent change in legislation, which was also present as an extrinsic factor or the discussion within the VPSG. The fact that the discussion within the VPSG could be identified as a trigger might be an indication that self-efficacy was also strengthened during the VPSG. This could be an indicator for behavioural changes [61].

### Focus group discussion

The benefits of VPSG as a CVME method were assessed using FGD. Through the concept maps method, and the subsequent coding of the answers, the feedback of the veterinarians could be condensed to the essentials.

The veterinarians felt that the VPSG could support and strengthen their relationship with their farmers. This could help less experienced veterinarians, as it is difficult for them to gain the confidence of farmers [44].

With regard to their therapy, diagnosis and a change in their antimicrobial prescription practice, veterinarians received new stimuli from the VPSG. This could be an indication that veterinarians are being encouraged by VPSG to reconsider their routine and possibly develop an intention to change. However, veterinarians also stated that they will not change anything in their therapy and antimicrobial prescription. Since all answers were listed without prioritization [54], we can unfortunately not determine whether the new impulses, or the persistence in old practices predominate.

The veterinarians were able to exchange with their peers on the topics of diagnostics, therapy and relationship to the farmer. In the decision-making process for the prescription of AMs, the peers of veterinarians play an important role [36,37]. This is an advantage compared to other CVME methods where most traditional teaching structures take place, teaching is not interactive and does not necessarily address the needs of the veterinarians [27].

### Limitations

Because only 23 veterinarians from 21 veterinary practices participated in the VPSG, our results cannot be extrapolated to the entire population of Swiss dairy practitioners. In addition, participants who attend a training programme voluntarily are more motivated than participants who are obliged to attend [62,63]. Some of the participating veterinarians are actively involved in national working groups and education. Therefore, the participating veterinarians were more interested, open-minded, progressive and willing to learn compared to the average Swiss cattle practitioner. However, through their position in education, some of these veterinarians could act as precursors.

Due to the facilitator's lack of experience, it might be that not all statements of all participants could be collected or that not all topics could be discussed. However, this could also have happened to an experienced facilitator, as this CVME method was new and unfamiliar to the participating veterinarians. It was unfortunately not possible to record the meetings and then transcribe them. Therefore, the meetings of the VPSG were recorded by one note-taker. Since it is almost impossible for one note-taker to write down everything that is said in a discussion of seven or more people, only the most important points were summarized and no verbatim transcription took place. This could have had an effect on the analysis, due to the fact that some words were not recorded. However, this proportion is likely to be small, because the note-taker was able to transcribe the essentials of the statements.

To our knowledge, VPSGs have never been used before to collect qualitative data. The fact that the veterinarians were not specifically asked about the possible drivers for their antimicrobial prescription practice might have led to an incomplete capture of the factors. On the other hand, the discussion among members of peer study groups gave detailed insight into beliefs and attitudes [64,65], which might have helped undisguised qualitative data to be obtained. This makes written records of peer study group meetings a good addition to qualitative data collection. As a consequence of the qualitative analysis, no statement could be made regarding which factors were more important for the decision to prescribe antimicrobials [66]. The fact that participants could choose the topics of discussion themselves was important to ensure interest and active participation of the veterinarians in the discussions. On the other hand, the variety of topics made a structured analysis of the data more difficult.

Since unfortunately only one veterinarian from the VPSG in eastern Switzerland took part in the FGD, the veterinarians' feedback on the learning effect of the VPSG could be biased. However, we consider this probability to be low as the veterinarians of the other VPSGs participated numerously in the FGD.

## Conclusion

With the written records of the VPSG, we were able to identify a variety of factors that influence veterinarians’ antimicrobial prescription practice. These extrinsic and intrinsic factors are partly dependent on each other and can therefore only to a limited extent be considered separately. Due to these interrelations, it is not likely to achieve a behaviour change of veterinarians, which might lead to a reduced antimicrobial prescription practice, by addressing just a single factor.

In the FGD, the veterinarians indicated that the VPSG could support them in their relationships with their farmers. They also gained new knowledge, new stimuli and the opportunity to exchange with their peers. Therefore, VPSG can address several factors and could be an important method to change the behaviour of veterinarians’ antimicrobial prescription practice. The next step is to analyse the antimicrobial prescription data. These data were collected from the participating veterinarians practice software during the study to show whether peer group training is an efficient way to reduce antimicrobial consumption.

## Supporting information

S1 Appendix Informed consent for veterinary peer study groups. (PDF)

S2 Appendix Abbreviated and anonymised written records of the VPSG and summary of the knowledge maps gained from the FGD. (PDF)

S1 Fig Code-co-occurrence-model of 35 codes summarized into 9 categories. (PDF)

S1 Table Number of meetings, topics and participation per VPSG. (DOCX)
