# Supplementary material for: Veterinary peer study groups as a method of continuous education—A new approach to identify and address factors associated with antimicrobial prescribing
Source: PLoS One. 2019 Sep 19;14(9):e0222497. doi: 10.1371/journal.pone.0222497 (PMC6752762; doi:10.1371/journal.pone.0222497)
Supplement: S1 Appendix — (PDF) [file pone.0222497.s001.pdf]

Declaration of consent veterinary peer study groups

Project name: A Field trial to reduce the sales of antimicrobials used for intramammary treatment at veterinary practice level using veterinary peer study groups

Project manager: Michèle Bodmer

Project participant:

Thank you very much for your participation in the veterinary peer study group on udder health and the focus group discussion. In the case of social science studies, the consent of each participant is required. In addition, each participant must be informed about what information will be used for the study and in what form. This declaration of consent is necessary for us to ensure that each participant has understood his or her participation and agrees with the terms and conditions that accompany it. For this purpose, we ask you to read this declaration of consent carefully and to confirm with a signature that you are willing to participate in the study, that you have understood what you have read and that all your questions have been answered.

- I will be one of 23 people participating in the three veterinary peer study groups.
- My participation in this project is voluntary. I understand that I will not be paid to participate. I can stop at any time without consequences. If I refuse or stop participating, no one will be informed.
- My participation means regularly participating in the peer study groups, participating in the focus group discussion and completing a questionnaire at the end.
- The veterinary peer study groups and the focus group discussion are noted or taped. Transcripts are created from the recordings. If I do not want to be noted or taped, I cannot participate in the study.
- If I feel uncomfortable during the veterinary peer study groups or the focus group discussion I have the right to leave at any time or to refuse an answer.
- The transcripts of the veterinary peer study groups and the focus group discussion will be sent to me. I can then correct any factual errors.
- The transcripts of the veterinary peer study groups and the focus group discussion as well as the completed questionnaire will be analysed by Valerie-Beau Pucken in the scope of a social science study on working groups in veterinary medicine.
- Valerie-Beau Pucken, colleagues and scientists involved in this project have access to the data collected during this study.
- Summaries of the veterinary peer study groups and the focus group discussion, which are made publicly accessible through scientific publications or other academic channels, are anonymised. Furthermore, no information or statement will be attributed to me or my practice.
- The records are stored for 5 years after publication.
- Any change, from the description above, will only be made with my explicit consent.
- - I have understood the above. All my questions were answered to my satisfaction and I voluntarily participate in this study.
- - I was provided with a copy of this consent form.

--	--
Signature participant	Date

-------------------------------------	--	--------------------------------------
Participant (written out)	Signature Valerie-Beau Pucken	Signature Michèle Bodmer

For questions or further information please contact Valerie-Beau Pucken.

Citation agreement:

I understand that I can be quoted directly. With that in mind, I agree with the following:

- I would like to see notes, minutes and other data that concern me and were collected during the study
- I agree that I can be quoted directly
- I agree that I may be quoted if my name is not published, or replaced by a pseudonym
- I agree that documents in which I am cited may be published

--	--
Signature participant	Date

-------------------------------------	--	--------------------------------------
Participant (written out)	Signature Valerie-Beau Pucken	Signature Michèle Bodmer

For questions or further information please contact Valerie-Beau Pucken.
