# Supplementary material for: Veterinary peer study groups as a method of continuous education—A new approach to identify and address factors associated with antimicrobial prescribing
Source: PLoS One. 2019 Sep 19;14(9):e0222497. doi: 10.1371/journal.pone.0222497 (PMC6752762; doi:10.1371/journal.pone.0222497)
Supplement: S2 Appendix — (PDF) [file pone.0222497.s002.pdf]

Protokoll Diagnostik

Mehrere Teilnehmer möchten wissen, was XYZ eigentlich alles an Diagnostik anbietet.

Experte:

- Amtliche Milchprüfung: Hemmstoff, ZZ, Keimzahl
- Milchleistungsprüfung Zuchtverbände:
    - Fett, Eiweiss, Harnstoff
    - Aceton für Startphasenkühe wenn gewünscht
    - Individuelle ZZ
- Milchdiagnostik: PCR Pathoproof C12 und M3
- PAG Test Milch
- Tierseuchenüberwachung Tankmilchserologie IBR, BVD, EBL im Auftrag des Bundes
- Wenig Testauswahl aber hohe Probendurchsätze

Vet: Wie beurteilen die Experten die PCR-Resultate aus den MLP Proben?

Beide Experten sehen es kritisch, da viel Umweltkontamination. Sie haben bei der Leitung angeregt nur noch Pathoproof aus sauberen bzw. aseptischen Proben zu machen.

Mehrere Teilnehmer äussern sich sich sehr kritisch zum Test und regen an, dass dieser Service eingestellt wird da sie mit diesen Resultaten die Landwirte nicht optimal beraten können.

Einhelliges Fazit: MLP Proben sollen aus dem Angebot genommen werden.

Vet: Ist eine Genotypisierung sinnvoll? Will XYZ es anbieten?

3 Teilnehmende verwenden Genotypisierung nach XYZ oder XYZ.

Experte sieht ein Angebot von Seiten XYZ vor allem als Teil eines PCR Packages in der Zukunft.

Experte Seht Ihr auch Herdenprobleme mit Non-GTB (GTB= S. aureus Genotyp B)?

Generell sehen sie wenig S. aureus Probleme, S. uberis ist ein grösseres Problem

Mehrere Teilnehmende bestätigen, dass sie z.T. Herdenprobleme mit Non-GTB Sehen.

Experte: Was macht Ihr dann?

Alle Teilnehmer werden sich einig, dass das Problem wie ein GTB Problem gelöst wird (Melkgruppen, Melkhygiene, Melkroutine…)

Es wird auch berichtet, dass es Betriebe mit sehr gutem Management gibt, die den GTB auf einer sehr niedrigen Herdenprävalenz halten können.

Vet: Was ist denn der Goldstandard?

Experten: PCR hat einen hohe Sensitivität und eine hohe Spezifität und entdeckt sehr kleine Keimmengen, aber nur von den Keimen die im Kit enthalten sind.

Experte: Ihr werdet von einem Neukunden beauftragt, ihm bei der Behebung seines Zellzahlproblems zu helfen, wie geht Ihr vor? Ihr habt keine Probenergebnisse von früher.

- Zellzahldaten anschauen auch retrospektiv.
- Tiere für Beprobung auswählen (hohe ZZ, neu hohe ZZ etc.)

Experte: Welche Tests?

- Einhellig: initial Kultur für Überblick, Leitkeim
- Die meisten schicken Proben einer Bestandesabklärung in ein kommerzielles Labor ein auch wenn sie zu Hause ein eigenes Praxislabor betreiben.
- Bei S. aureus dann mit PCR weiterfahren

Vet: Werden Pathoproof Resultate international gleich ausgegeben?

Experten: Ja, da gibt es Herstellerangaben dazu (Ct 37 und Ct 34 für andere Staph.)

In den USA untersuchen sie fast ausschliesslich die Kuhassoziierten Keime.

Experte: In Norwegen untersuchen sie gerade wie stark die automatisch gefassten Roboterproben kontaminiert sind.

Vet: Kann ein Nicht Zuchtverbandsmitglied auch Proben schicken für die ZZ und die Milchinhaltsstoffe?

Experten: Ja das kostet 1.50 /Probe

Bakteriologie Bern: Blutagar und Malditof = mittlerweile Routine fürs gleiche Geld

„Neue" Tests wurden besprochen:

XYZ kennt nur 1 Vet (Farbumschlag nach 48 Std. Kultur) XYZ??

XYZ Test kenne Vet (schlechte Erfahrung) und Vet (gute Erfahrung). Vet hat danach gefragt. (Farbige Kulturen auf Agarplatten) XYZ- Mastitis-Schnelltest

Erfahrungen untereinander austauschen

- Wie sieht dein Praxislabor aus?
- Wo hast du das, was du bei deinem Praxislabor anwendest, gelernt?
  - > Diese Fragen wurden ausgiebig besprochen.
- Welches war der letzte Fall, bei dem du Schwierigkeiten in der Diagnostik hattest?
  - > Diese Frage wurde schon in der Runde vorher besprochen
  - >

Vet: Auffällig viel S. uberis Befunde aus XYZ; Problem der Diagnostik?

Exp: Identifikation immer noch heterogen.

Vet: Wie geht Ihr generell vor und wie differenziert Ihr Staph und Strep? Finde es manchmal schwierig

Vet: Gelernt in Praxis 20-25 Fr./Probe

- Ausstrich mit Tupfer auf Blutplatten ½ Platte (Tupfer 10s in Probe)
- Ablesen im 12 h Takt
- Morphologie
- Katalase, Indolprobe
- Evt Gramfärbung
- SAED Platte für Staphylokokken
- Penplättchen

Vet 10 Fr/Probe

- Giesst Milch über Blutplatte
- Hat noch Selektivplatte von XYZ stimmt nicht immer so genau
- Penicillin-Neomycin und Cephalosporin Plättchen
- Schickt oft auch Platte oder Probe ins kommerzielle Labor wenn eine Weiterdifferenzierung notwendig ist

Vet: Gelernt in Praxis in Deutschland

- Giesst Milch über Blutplatte
- V.a. bei akuten Mastitiden G- G+ Unterscheidung

Vet: Hat es von TPA mit Laborerfahrung gelernt 21 Fr./Probe

- Mit Öse ausstreichen auf Blutplatte
- Morphologie, Hämolyse
- Katalase
- Gramfärbung immer
- Test neues Kit von XYZ zur Keimidentifikation und Resistenzuntersuchung

Stimmt rel. gut mit Routineprotokoll überein

Zusammenfassung Experte: rel. grosse Variabilität schon beim Inokulum (Tupfer, Öse = Standard, draufgiessen) dies ist im kommerziellen Labor sicher etwas standardisierter aber die Keim Spezifizierung ist dort auch nicht überall gleich. Goldstandard wahrscheinlich Malditof ab Kultur mit guter Datenbank.

Exp:Wer macht routinemässig Bakt vor dem Trockenstellen?

Vet: Biobetriebe konsequent, andere zum Teil.

Fazit alle: Wir müssen rausfinden, wie weit wir mit unserer Diagnostik kommen, evt auch vereinfachte Tests anwenden.

"Diagnostikrichtlinien" Praxislabor

Übereinstimmend: Wir sind keine Experten, daher können wir keine Diagnostikrichtlinien erarbeiten.

Wir können und wollen aber PRAXISRICHTLINIEN für unsere eigenen Praxen erarbeiten:

> Was ist seriös/ wie unterschiedlich sind denn eigentlich die Resultate
> Wie weit komm ich mit meinem Labor,Wo sind meine Grenzen
> Wie viel Standardisierung bringt’s?
> Was lohnt sich auch kostenmässig für meine Praxis?

Expertenrunde 2

- "Diagnostikrichtlinien" erstellen
  > Jeder soll für sein Labor seine eigenen, individuellen Richtlinien erstellen:
  jede Praxis muss für sich überlegen: „Was wollen wir, wie weit wollen wir gehen?“
  > Bestandesproblem und nicht eindeutige Fälle werden gerne an externes Labor geschickt

- Antibiogramme
  > Plättchentest :
  schon im akkreditierten Labor schwierig (unterschiedliche Richtlinien, in Europa: Europäische Richtlinie (EUCAST): wollen verbindliche Veterinärmedizinische Richtlinien erstellen (Zukunft wird evtl. besser?)

sehr komplex, bestimmte Platte, bestimmte Verdünnung

Experte lasst die Finger davon als Praxislabor!

> Antibiogramme im kommerziellen Labor zuverlässig?

VET Jedes Labor macht es anders, jedoch deckt es sich hauptsächlich schon.

> In vivo vs. In vitro

Keime haben verschiedene „Mechanismen“ um sich Antibiotikum zu entziehen

S. Uberis kann in Zellen gehen und wird evtl. von Schleimkapsel geschützt.

S. Aureus kann in Zellen gehen; Staph. die nicht in Zellen sind, „schwimmen“ in Milch, „kleben“ an Zellwand: erwischt man gut

Mykoplasmen erwischt man nicht, Pasteurellen erwischt man schwierig

Jedoch gilt eigentlich: wenn in vitro empfindlich, dann auch in vivo empfindlich.

> Ist Antibiogramm überhaupt sinnvoll?

VET Ob Antibiogramm sinnvoll ist hängt vom Erreger ab.

Antibiogramm empfehlenswert bei:

1. Staphylokokken (Penizillinresistenz liegt bei ca. 40 %)

   M. behandelt diese nicht unbedingt, ausser wenn erhöhte Zellzahl über längere Periode oder Klinik, Kühe können AB trockengestellt werden

   M. Erfahrungen: Sensibel auf Aminoglykosid, Amxocillin, Ampicillin

   Resistent auf Penicillin, Cloxacillin

2. S. aureus (penizillinresistent 15-25%)
3. Klebsiellen (bei Bestandesproblem)
4. E. coli
5. Enterococcen

   laut XYZ eher empfindlich auf Amoxicillin, Clavulansre

   Penizillinresistent: 40-50 % (je nach Land)

Antibiogramm nicht empfehlenswert bei :

6. Serratia (weil Prognose eh schlecht ist)

> Wenn man direkt ans Labor schickt, weil man kein Praxislabor hat, kann man mit Labor absprechen in welchen Fällen man kurz Rücksprache hält wegen eines Antibiogramms.

- Resistenzmechanismen:

> Staphylococcen haben spezifisch beta-lactamase als Resistenzmechanismus

> Streptococcen haben andere Resistenzmechanismen (verändern Penicillin binding protein und werden so Penicillin resistent (Enterococcen genauso)

Strep Uberis:

a. verminderte Penicillin Empfindlichkeit (keine Resistenz)
b. Cloxacillin resistent
c. Ampicillin sensibel
d. Aminoglykosid nicht sehr sensibel
e. Erfahrung bei Betriebe, der viel Makrolide eingesetzt hat: Makrolid Resistenz

- Wie viel Routine braucht man:
  > Je mehr je besser, jedoch davon abhängig, wie viel man machen möchte

- Agarplatten
  > Chromogene Agarplatten werden immer mehr benutzt (schwierig bei Mischflora)
  > Klassische mit Blut und Mc Conkey werden auch häufig genutzt
  > VET hat gerne wenn man Hämolyse sieht (Blutagar)
  > Mit welchem Agar man arbeiten möchte kommt aufs Praxislabor drauf an: was will man machen?
  > EXP Selektivplatte gleiche Sensitivität wie bei Blutplatten?
  > VET Blutplatten natürlich weniger selektiv- kein Hemmstoff. Es gibt jedoch kein Muster, mal wachsen welche nur auf chromogenem Agar, mal nur auf der Blutplatte

- Proben zentrifugieren?
  > VET Wir vortexen die Probe. Zeitfaktor! Vortexen geht schneller
  > Je nach Erreger ist Sediment oder gevortexte Probe besser

- Schnelltests
  > XYZ
    1. Antibiogramm, Mykoplasmen
    2. relativ teuer (im Einkauf 25 CHF im Einkauf, 40 CHF verlangt Tierarzt)
    3. VET Fand es umständlich, was ist mit Mischflora? Kennt Tierarzt, der damit arbeitet und es funktioniert super.
    4. VET Hat es auch mal gemacht und mit Ergebnis im Labor übereingestimmt. Relativ einfach zum Ablesen, jedoch nicht für jede Milchprobe geeignet

  > XYZ LDH Test: Sehr unspezifisch (gleich Info wie beim Schalmtest)
    1. VET nimmt es bei Frischgekalbten, da diese im Schalmtest eh anschlagen. Als Routine Test nicht geeignet
    2. Vet Ist dieser Spezifisch?
    3. EXP Müsste ich schauen, ob Daten in diese Richtung ausgewertet wurden.
    4. Nachfrage bei Olga Wellnitz: LDH in der Milch geht hoch wenn die Blut-Euterschranke sich öffnet. Ist v.a. bei akuten gram negativen Mastitiden deutlich (LPS).

- Beurteilung der Güte eines Labors?
  > Akkreditierung ? Akrreditiert heisst, dass die Institutionen regelmässig überprüft werden und u.a. auch an Ringversuchen teilnehmen.
  > Wie werden die Ergebnisse/Informationen berichtet, aufgelistet?
  > Im Zweifel mal anrufen
  > Falls ein Test nicht akkreditiert ist, müssen Labore das auflisten

- Milchprobenlagerung:
  > Kühlen oder gefrieren (könnte positiv sein, da Zellen kaputt gehen und man somit intrazelluläre Erreger evtl. auch bekommt)

- Sterile Milchprobe bei Schalmpositivem Test:
  > Mykoplasmen (sieht man jedoch meistens schon klinisch)
  > Erreger brauchen z.T. länger als 24 h zum Wachsen:
    - Mykobakterien
    - Atyp. Mykobakterien, Nokardien (brauchen 1 Woche zum Wachsen)
    - Pyogenes braucht evtl. länger

        - Hefen, aber die kann man im Direktausstrich mit Gramfärbung erkennen
    - > Nicht infektiöse Mastitis: bei Mastitis meistens Bakterien?
        - Evtl. chemischer Reiz, oder Trauma
        - Stierig
        - Stress
        - Windviertel?
        - Bei Fieber evtl. E. coli (Würde man mit PCR nachweisen können)
            - o Gibt es eine Studie? Müsste M. mal nachschauen
        - Wetterwechsel ist auch ein Stressor
    - > Vieren? Herpesviren sind schon mal diskutiert worden.
    - > Bei steriler Milchprobe durch einfrieren evtl. noch was rausbekommen?
        - Exp: Kann man probieren, lohnt sich jedoch die Mühe für den Ertrag? Evtl. vorher länger bebrüten.

- Wie verschickt man am besten Kolonien zu einem externen Labor?
    - > VET von der Biosafety her darf man keine Platten verschicken. Am geschicktesten ist ein Tupfer mit Medium und der Kolonie, wo man am ehesten denkt, dass es sie ist

- Experte merkt an, dass Praxislabore im Moment noch nicht kontrolliert werden, das könnte sich jedoch in den nächsten Jahren ändern (Qualitätskriterien und Abfall dekontaminieren!)
    - > Informationen stehen in der Einschliessungsverordnung:
    - > Bakterien werden in 4 Gruppen unterteilt:
        1. Umweltkeime
        2. Wenig pathogene (Mastitis)
        3. Pathogene
        4. Pathogene für Hochsicherheitslabor

      Praxislabor: Klasse 2 Labor, müsste eigentlich beim Bundesamt für Umwelt; Thema Biotechnologie angemeldet sein. Anmeldung funktioniert gut, ist jedoch Papierkrieg.

Austausch:

VET
Ausstrich mit Tupfer auf Blutplatten ½ Platte (Tupfer 10s in Probe)

- Ablesen im 12 h Takt
- Morphologie
- Katalase, Indolprobe
- Evtl. Gramfärbung
- SAED Platte für Staphylokokken
- Penplättchen

VET
Giesst Milch über Blutplatte

- Penicillin-Neomycin und Cephalosporin Plättchen

VET

- Giesst Milch über Blutplatte
- V.a. bei akuten Mastitiden G- G+ Unterscheidung

VET

- Mit Öse ausstreichen auf Blutplatte
- Morphologie, Hämolyse
- Katalase
- Gramfärbung immer

Experte merkt noch an, dass es vorgefertigte Ringversuche gibt

Feedbackrunde

Stimmungsabfrage: Alle kleben gelbe Punkte aufs Smiley Blatt.

- Interessant aber keine klare Beantwortung der Frage wie weiter mit meinem Praxislabor
- MikrobiologIn hat gefehlt
- Durchdenken welcher Test für was war sinnvoll (UB)
- Austausch von Ansätzen sehr interessant
- MLP Proben mit PCR untersuchen taugt nicht
- Evtl. häufiger Proben in ein kommerzielles Labor einschicken, wenn man nicht sicher ist
- Gut zu wissen, was XYZ alles macht
- Entsorgung der Platten etc. muss auch besprochen werden bzw. müssen da auch Richtlinien für Privatlabors geschaffen werden

Protokoll Bestandesbetreuung

Erfahrungen austauschen

Fragen an Pinnwand:

> Bestandesbetreuung: wie geht ihr vor?
  - Macht ihr einen Termin mit dem Landwirt zur Bestandesbetreuung aus?
  - Wie oft im Jahr macht ihr das?

> Stolpersteine bzw. was kann fehlen?

> Wie ist das Bedürfnis der Tierhalter nach Bestandesbetreuung?

VET: Ich habe einen Betrieb mit Staph Problem. Tank-Zellzahl war erhöht. Schalmtest habe ich gemacht und ich durfte die Milchprobe von 4 Kühen ansetzen, Staph aureus ist bei mir gewachsen. Auf Nachfrage durfte ich dann die Probe weiterschicken.

VET: Ich lass mir die ZZ vom letzten Jahr geben (zur Not den Ordner), Schalmtest nur bei Tieren, wo es etwas bringt, sprich die eine erhöhte Zellzahl zeigen.

VET: Wie bekommst du Landwirte zum Mitmachen?

EXP: Die Schmerzgrenze muss einfach hoch genug sein.

Mehrheitlich: Diese ist sehr individuell! Wenn Landwirt es nicht als Problem sieht bist du Chancenlos.

VET: Bei mir ist es so, dass wenn ich mich wirklich mal mit dem Landwirt hinsetze um alles anzuschauen/durchzusprechen, dann kommt die Initiative vom Landwirt aus. Wenn ich den Landwirt darauf anspreche, warum ZZ hoch ist, dann kommen eigentlich nur Ausflüchte

VET: Das Bedürfnis muss da sein, dass sie was machen wollen ->Abzug beim Milchgeld

VET: Bei uns kommen die LW oft zu spät. Dem Tierarzt machen sie Vorwürfe, dass nichts gefunden wurde in der Probe. Wir sind generell selten diejenigen, die sie zur Hilfe ziehen. Sie bringen uns die Milchprobe zum Ansetzen, jedoch kommen wir selten auf den Betrieb. Wir haben 3 Betriebe, die auf einmal komplett ohne TS trockenstellen und die beschweren sich jetzt, dass es nicht funktioniert. Allerdings wurde ein mögliches Vorgehen im Vorfeld nie mit den Betriebsleitern diskutiert.

Wie komme ich da wirklich rein?

Und wenn ich einen Strep nachweise. Was mache ich dann? Soll ich weiter testen? Soll ich häufiger nach den Daten fragen? Milchwägedaten?

VET: Ich schicke bei Betrieben die keine Milchwägung haben, die Milchproben von jeder Kuh zu XYZ um ZZ zu messen, das gibt dann einen Überblick über die momentane Situation und verschafft dem Betriebsleiter auch etwas Zeit. Bei den Zuchtverbandsbetrieben erst um Ordner mit ZZ bitten und sich das mal anschauen. Dann um einen Termin. So hat LW Zeit sich darauf vorzubereiten.

VET: Ich würde nach dem XYZ Zugang fragen. Dann bekommst du auch schon ganz viele Daten. Das geht natürlich nur für Betriebe die bei den Zuchtverbänden sind.

EXP: Als aller erstes muss Bereitschaft da sein, etwas ändern zu wollen.

VET: Wenn du die Proben zu XYZ schickst findest du immer einen Ansatz um mal irgendwo anfangen zu können.

VET: : Ich habe einen Betrieb, wo LW im Labor arbeitet. Betrieb hat von 100 auf 0 mit den Trockenstellern aufgehört. Jetzt hat LW alle Einzelviertel- Milchproben auf einer Graphik dargestellt. Ganz schwer LW davon zu überzeugen, dass das nichts bringt und eine Bakteriologie gemacht werden muss

VET: Das ist eine blöde Situation

EXP:. ZZ ist ja nicht die Ursache, sondern die Folge. Natürliche Abwehr vom Körper. Du behandelst ja nicht die hohe ZZ sondern den Verursacher.

VET: Die ZZ ändert sich ja von Tag zu Tag, das ist immer nur eine Momentaufnahme.

EXP: Um nochmal auf Vet Problem zurückzukommen. Was macht ihr mit Landwirten, die keine grössere Intervention wollen aber trotzdem immer etwas „vorwurfsvoll Jammern“? Da steht man doch an der Wand.

VET: Solche kommen einfach zu dir und sagen: Gib mir 50 Tuben.

VET: Das sterile Bakteriologie was Positives ist, verstehen die meisten nicht. Das sagt mir ja: OK ich schau jetzt woanders, ob ich da was finde. Sie empfinden es als einfacher was nachzuweisen, als den ganzen Betrieb auseinander nehmen zu müssen.

VET: Manchmal bietet sich ein Einstieg mit ein paar Standardfragen an etwa: „Wann hattest du den letzten Melkmaschinen-Service?“ „Hast Du irgendwas im Bereich Melken geändert in den letzten 3 Monaten?“ ist auch ganz geschickt, wenn man unter Zeitdruck ist.

EXP: Wenn die Landwirte im Zuchtverband sind, dann ist es das einfachste sich die Daten von denen anzuschauen. Dazu müssen sie bereit sein, einem das Login zu geben bzw. Dich als Berater freizuschalten.

VET: Wenn LW wirklich will, dann macht es Sinn sich das gemeinsam anzuschauen. Wenn er nicht will, dann bringt auch das nichts.

VET: Oder er hatte den XYZ schon da.

EXP: Ja, XYZ oder den Melkberater

VET: Den Melkberater höre ich auch oft.

EXP: Schade, dass es da zu wenig Zusammenarbeit gibt.

Welche anderen Mitbewerber gibt es denn noch?

VET: XYZ vor allen Dingen. Aber auch Homöopathen.

VET: Wie den XYZ Der verkauft ja für alle das passende. Oder die XYZ von XYZ

VET: Ich habe noch nie von einem Landwirt explizit gehört, dass er einen anderen Berater dahatte. Fragt ihr das explizit eure Landwirte?

VET: und VET: Wir fragen das explizit.

EXP: Gibt es weitere Mitstreiter?

Vet: Ja, die den Kriechstrom feststellen

VET: Der XYZ zum Beispiel aber der arbeitet ja nicht mehr bei XYZ.

EXP: XYZ von XYZ kann das recht zuverlässig. Ich kann euch mal Adressen von Ansprechpartner zusammenstellen dafür.

VET: Ich hatte einen Betrieb, der wollte mit Bioresonanz sanieren. Letztlich war es doch nicht so erfolgreich.

VET: Es gibt auch noch die Fernheiler übers Telefon.

EXP: Habt ihr das Gefühl, das die Landwirte viel selber ausprobieren?

VET: Nein, das Gefühl habe ich nicht. Ich bin ziemlich schnell mit involviert. Viele kommen bei mir von sich aus und fragen um Rat.

VET: Die, die was wollen rufen mich an. Bei den anderen kommt der Melkberater, der wird ja dann von den Verarbeitern geschickt wenn die Milchqualität eines Produzenten zu schlecht wird.

VET: Die meisten kommen zeitig, bevor Dilemma da ist.

VET: Die chronischen Problembetriebe kommen eh nicht zu mir.

VET: Bei einem Roboterbetrieb mit riesen Problem, da bin ich erst ganz zum Schluss gerufen worden. War ein S. uberis Problem. Und nachdem ich meine Vorschläge genannt hatte war ich nicht mehr erwünscht.

EXP: Sind das denn Betriebe, wo auch oft andere Probleme sind?

VET: Ja, ganz oft ist bei denen auch die Fütterung ein Problem.

VET: : Die sind oft eh am Schwimmen.

VET: Die müssen Geld sparen.

VET: Ich finde es schwierig zu sagen, dass meine LW gar nichts machen wollen. Aber ich komme ja auch nie in den Betrieb.

VET: Glaubst du aus Kostengründen, oder weil sie sich schämen für Ihre Sauerei?

VET: Kostengründe!

VET: Lass sie doch schmoren, bis es explodiert.

EXP: Haben die LW vielleicht auch Angst vor Mehraufwand?

VET: Ich habe auch einen Laufstallbetrieb mit S. aureus Problem. Da habe ich gefragt, ob sie was machen wollen. Der Junge ganz klar: „Ja!“, der Alte hat nur geantwortet, dass es ja auch andere Betriebe mit S. aureus-Problemen gibt. Daraufhin habe ich gesagt: „Ja, die haben aber kein Problem.“ Nun rede ich mit dem Jungen und kläre das vorher ab. Und wenn es dann konkret wird erkläre ich dem alten LW was jetzt gemacht werden muss.

VET: : Wenn nach einem halben Jahr nichts passiert oder der Betrieb ist wieder schlecht, dann frage ich nach: Hast du das gemacht? Wenn dann ein Nein kommt, braucht man sich auch nicht zu wundern.

VET: Wenn wir nach einem halben Jahr am gleichen Punkt wie zuvor sind, dann ist mir meine Zeit und meine Mühe zu schade. Wenn nichts umgesetzt wird kann man sich das auch alles sparen.

EXP: Es gibt auch die anderen, die nach 6-8 Monaten fast gut sind.

VET: Bestandesbetreuung macht nur Sinn, wenn alle es wollen. Alle müssen an einem Strang ziehen. Die Bedürfnisse sind extrem unterschiedlich.

VET: Manche haben auch Angst vor Routinewechsel. Für Tipps wären sie eigentlich zu haben, aber haben Angst vor Kosten.

EXP: Was sind denn Stolpersteine?

> Individuelles empfinden
> Fehlende Daten oder Sie liefern sie nicht

EXP: Wie oft redet ihr mit Betriebsleiter?

VET: Am Telefon oft, aber auf einen Betrieb selber max. einmal im Jahr

VET: J: Einmal im Monat ein Gespräch in die Richtung und 4-5 mal im Jahr, dass man dann auch wirklich was angeht

VET: So 2-mal im Monat

VET: Bei mir Ähnlich. Der erste Schritt ist häufig, aber das man dann wirklich was macht ist eher schwer.

VET: Die, die wir lange kennen betreuen wir (7 Betriebe in der Bestandesbetreuung) aber wenig zusätzliche Fälle

VET: Ich bekomme viele Milchproben. Dann spreche ich die heiklen, oder die, die oft eine Milchprobe bringen selber schon an. Manchmal geht es weiter. Im Moment habe ich 2 mit Bestandes Sanierung.

VET: Wir diskutieren viel und sanieren dann 1-2 im Jahr. Die anderen (wenige) nehmen evtl. auch gar keine Milchprobe (Dunkelziffer). Insgesamt sind aber die Stimmung und die Vertrauensbasis anders als bei Vet.

EXP: Bestimmt ist es auch eine andere Kundschaft.

VET: Die Betrieb die an Käserei liefern ticken ganz anders als Betriebe, die nur Milch abliefern. Die Käsereibetriebe haben ihre Melkmaschinen im Griff. Allerdings neigen sie zur schnellen Behandlung von Kühen mit nur geringgeradigen ZZ Erhöhung um die Qualitätslimite der Käserei sicher zu erreichen.

Und grosse Betriebe haben auch viel Verdünnung in der Tankmilch. Da gibt es auch einen Unterschied.

VET: Wir haben auch einen grossen Betrieb, der läuft Tip-Top, macht aber nie einen Wechsel bei der Melkmaschine. Es funktioniert halt so.

EXP: Es wird immer die Ausnahmen vom Standard geben.

Fazit:

Die individuelle Schmerzgrenze bei den Produzenten spielt eine riesige Rolle, wann und ob ein Eutergesundheitsproblem angegangen wird. Dies bedeutet, dass man auch als Tierarztpraxis kein unverrückbares Standardvorgehen anbieten kann sondern individuell auf die Betriebe eingehen muss.

Vorschlag von Exp für Dauer-Jammernde Betriebsleiter: Ganz konkret nach ihren Zielen fragen! Dies führt beim Einen oder Anderen dazu, dass sie/er sich mal Gedanken macht über die Situation und evtl. auch erst Monate später in eine Zusammenarbeit einwilligt. Klar kommunizieren, was von Eurer Seite möglich ist und was nicht, keine falschen Hoffnungen machen;)

Expertenrunde:

Beispiel von VET :

Der Bestand:

- Milchviehbestand mit ca. 32 Holstein Milchkühen
- Boxenlaufstall
- Tandemmelkstand mit 4 Plätzen, einzeln gesteuert
- Tages oder Nachtweide hauptsächlich Naturwiese
- Fütterung im Stall: Heu, Gras-, Maissilage als Einzelkomponenten, Kraftfutter ab Station.
- Kein Mitglied in einer Viehzuchtgenossenschaft, keine Zellzahlen oder Milchinhaltsstoffe für Einzeltiere.
- Aufzeichnungen der monatlichen Untersuchung mittels Schalmtest sind lückenhaft

Problem:

- Immer wieder Episoden mit erhöhter Tankmilchzahl.
- Ältere Kühe, mit chronisch infizierten und generell ungesunden Eutern (schlechte Euteraufhängung, veränderte Zitzen) will er lange nicht ausmerzen
- Aus Kostengründen sagt er lange nichts und versucht immer wieder mit selber applizierten Euterinjektoren die Tiere zu behandeln. Nach der 2. oder 3. Beanstandung meldet er sich jeweils und möchte eine rasche und billige Lösung (Mit dem Bezahlen der Rechnungen ist er sowieso immer im Verzug).
- Immer wieder eher magere Kühe mit Schwächeanzeichen, teilweise Zusammenbrechen im Laufstall oder Festliegen
- Klauengesundheit ist oft mangelhaft

VET Einschätzung:

Ein grosses Problem liegt meines Erachtens in der Haltung und Fütterung (hier haben auch schon Speziallisten aus der Futtermittelbranche erfolglos versucht einzugreifen).

Die Folge sind wegen hygienischer Mängel und eher schlechter Klauenpflege dann eben auch Zitzenverletzungen, und schlechte Eutergesundheit. Empfohlene Massnahmen scheitern meistens am Geld, die sie kosten würden bzw. an der benötigten Zeit.

VET Lösung:

- Bei jeder Kuh Schalmtest während dem Melken, schreibe alles auf,
- entnehme Milchproben wo nötig
- versuche einen Leitkeim zu bestimmen

Massnahmen vom Landwirt:

- feuchte Einmaltücher zur Euterreinigung hält er eine gewisse Zeitlang durch
- Zitzentauchen tut er, aber eben oft mit einem ungenügend desinfizierenden Mittel.
- Die Melkmaschine muss er jeweils kontrollieren lassen, was meistens auch gemacht wurde (externer Sündenbock).
- Für weitere Massnahmen finde ich oft zu wenig Gehör, bzw. wenn sich die Situation wieder etwas bessert ist der Zusatzaufwand nicht mehr nötig und der Mann fällt wegen Arbeitsüberlastung rasch wieder ins alte Muster zurück.

VET: Die Festlieger: sind das Frischkalbende?

Mod: Da VET alles recht detailliert beschrieben hat, denke ich, dass er diese Info sonst dazu gegeben hätte.

VET: Er hat halt einfach kein Geld

EXP: Der befindet sich in einer Negativspirale.

VET: Sollte er nicht besser aufhören?

VET: Wenn ein Landwirt jammert und jammert sag ich ihm: dann hör doch auf

VET: : Dann sagt er: Was soll ich denn dann machen?

VET: Ja, aber doch nicht weiter negativ schaffen.

VET: : Dann kommt die Antwort: Ich muss ja nicht mehr so lange.

VET: Ein Kunde von mir will aufhören, wenn ich aufhöre☺.

EXP: Schaut ihr auch auf „Schwäche“ und „Klauen“?

VET: Kranke Kühe können keine gesunde Milch geben.

EXP: Kranke Kühe sollte man ausmerzen, wenn das dann vom Zustand her noch geht.

VET: Zu dem Futter: Wie viel gibt er vor? Wie ist die Qualität? Essen sie alles? Die Fütterung scheint falsch, nicht angepasst.

EXP: Es gibt ja viele magere Kühe. Das könnte auch von den Klauen kommen. Man sollte nicht nur vom Euter aus schauen, aber eben die Fütterung ist sicher wichtig! Mir scheint die Versorgung etwas am Limit für Holsteinkühe, mit Einzelkomponenten ist er schnell limitiert bei der Grundfutteraufnahme und auch vom Arbeitsaufwand her. Aber Mischwagen ist teuer….

VET: Ansatz von Vet finde ich gut. Ist Kostenarm.

VET: Milchproben zu nehmen ist wichtig.

VET: Ich habe mal ausgewertet wie gut unsere Landwirte Milchproben nehmen. Wir hatten nur 8% Mischflora. Beim Tag der offenen Tür haben wir Platten mit Mischflora und Platten mit Einzelkultur gezeigt. Das war ein gutes Aha-Erlebnis für Landwirte.

VET: : Ja, dann wissen sie, wovon man redet.

EXP: Bei so wenig Spielraum bleibt fast nichts anderes als der Vorschlag vom Vet. Ich würde evtl. auch schauen, ob ein ausmerzen von einzelnen Tieren für Landwirt in Frage kommt. Und dann eine Prioritätenliste fürs Ausmerzen zusammenstellen.

4 Viertel Milchprobe?

Wenn Geld knapp ist, ist eine 4 Viertel Milchprobe besser als nichts.

VET: in Deutschland gibt es nur Einzelviertel -Milchproben. Da kostet aber die Probe auch fast nichts

EXP: Wenn du den Schalmtest vorher machst, kann du ja auch nur die Viertel beproben, mit den höchsten CMT Resultaten. Das reduziert dann auch die Kosten und steigert die Chance, dass man auch den wahren Verursacher findet.

Ohne eine Milchwägung ist eine Verfolgung der Daten nicht möglich.

VET: Bei Kühen, die über 3 Wochen nach der Kalbung sind würde ich einen Ketontest machen. Im Hinblick auf Fütterung wund weil ich Kuh als Gesamtbild sehen möchte.

EXP: Der Mann scheint überlastet. Auch beim Thema Tierschutz ist es da eine Gratwanderung.

VET: : Ich sage in solchen Fällen dann zu meinen Landwirten, dass wir ein Problem haben werden, wenn jemand kommt. Also wir beide;)

VET: Das sag ich auch.

EXP: Und, passiert dann etwas?

VET: : Bei solchen wird es meist schwierig.

EXP: Vet, Du hast eben empfohlen Proben bei XYZ einzuschicken wegen der Zellzahl.

VET: Ja, das ist gar nicht so teuer.

VET: Und man kann noch die Inhaltsstoffe bestimmen lassen.

EXP: Bei dem Landwirt gibt es wenig Anhaltspunkte. Wie kennen die Milchinhaltsstoffe nicht. Fütterung ist zu wenig oder schlechte Qualität. Ich hab Euch die Preisliste von XYZ mitgebracht und ein Antragsformular für Nicht-Zuchtverbandsmitglieder. Die Zellzahlbestimmung kostet 2.80 CHF/Probe.

VET: : Der will doch eh nichts ausgeben

EXP: Ja dann bleibt man der Feuerlöscher, wenn es eskaliert.

Beispiel Eutergesundheitsproblem: keine Milchkontrolle (Experte)
Vorbericht

- Hohe Tankzellzahlen (Ablieferungsmilch!!) sind seit Jahren ein Problem auf dem Betrieb.
- Immer wieder akute klinische Mastitiden: guter Behandlungserfolg
- Subklinische Mastitiden nur schwer heilbar trotz Behandlung
- Vor 2 Jahren S. aureus eher sporadisch im Bestand, Trägertiere ausgemerzt

- Melkmaschinenservice gemacht, keine Beanstandungen

Befunde auf dem Betrieb

Kennzahlen soweit berechenbar

- Melkarbeit: kein Vormelken, verspätetes Zitzentauchen
- Zitzenkondition bei 13% der Kühe schlecht = stark verhornt
- Verschmutzungsgrad der Euter bei Gustkühen zu stark! (Tiefstreu!)
- Keine Melkreihenfolge….

Andere Erkrankungen:

- 10% der Kühe liegen nach dem Kalben fest!

EXP: Wir haben uns von DeLaval ein portables ZZ-Messgerät geliehen. Dann haben wir alle beprobt, von denen die ZZ erhöht war. Wir haben zuerst vorgemolken und dann innerhalb von 30 sek die Probe genommen. Das ist somit relativ standardisiert.

Kosten liegen etwa bei 3 CHF pro Kuh. Mietkosten fallen nicht an ausser ein Depot, das bei intakter Rückgabe rückerstattet wird.

Es war zwar aufwändig, hat sich aber gelohnt. Das ist eine gute Alternative, wenn keine Milchwägung vorhanden ist.

Wir haben 4 Viertel Milchproben für Bakteriologie genommen.

Der Hoftierarzt hat später auch nochmal Proben genommen. Da kam nicht wirklich etwas anderes bei raus. Bei XYZ kostet es 2,8 CHF für ZZ und, Fett und Eiweiss kostet nochmal 2,8 CHF. Die Hefen Kuh wurde ausgemerzt.

VET: Wie war die Melkanlage?

EXP: Aufgrund der stark veränderten Zitzenenden hatten wir den Verdacht, dass etwas nicht stimmt. Die Zitzengummis wurden geändert. Ganz wichtig war aber, dass das Timing beim Melken nicht gepasst hat. Dies ist eben problematisch beim Swingovermelkstand. . Ein unabhängiger Milchberater ist für die Beurteilung und Nassmessung der Anlage gekommen. Am Schluss war es hauptsächlich ein Melkproblem. Eine Weiterverfolgung geht nicht, da keine Milchwägung gemacht wird. Ist natürlich unbefriedigend.

EXP: Gibt es zusätzliche Ideen?

VET: Regelmässig einen Schalmtest machen und dies immer dokumentieren.

VET: Hättest du C. Bovis von denen behandelt?

VET: Dann hätte er ja gar keine Milch mehr zum Abgeben gehabt.

EXP: Wir haben aufs Laktationsstadium geschaut und die Kühe dann evtl. früher Trockengestellt. Und wir haben das ganze Stufenweise gemacht. Die, die evtl. behandelbar sind, behandelt, die zum Trockenstellen Trockengestellt.

VET: Kennt ihr das rezidivierende Kühe nicht mehr Antibiotisch zu behandeln, sondern nur mit Dolovet oder Rifen zu behandeln?

VET: Ich habe das jetzt 3-mal gemacht. Eine ist gut geworden, eine habe ich erst heute behandelt und die andere habe ich nicht mehr verfolgt.

VET: Das funktioniert prima übers Wochenende. Man soll Milchprobe nehmen und Rifen geben. Dann ist gut.

VET: : Wie lange behandelst du?

VET: Ich habe für 4 Tage Dolovet mitgegeben.

VET: Provozierst Du da keine Labmagengeschwüre?

VET: Ich habe es mal geschafft nach 16 Tagen Dolovet eine Kuh, die einen Unfall hatte, ins die dann schwarzen Mist gehabt hatte. 14 Tage lang war es unproblematisch. Die Kuh hat es aber mit Transfusion überlebt.

EXP: Vet, du hattest doch mal einen Roboterbetrieb mit einem Problem, weitere Erfahrungen mit Roboterbetrieben?

VET: Ja, der hatte nicht genug Fressgitter und wollte nur von der Hälfte der Kühe eine Milchprobe einschicken. Wir haben dann nur mit den Zellzahlen gearbeitet, die Erdung vom Roboter korrigiert, die Verunreinigungen weg und die schlimmsten Behandelt. Aber so richtig gut ist es nicht geworden.

VET: : Wir haben im letzten Jahr einen gehabt. Viele akute Viertel, die nach 24h tot waren. Nach einer Wasserprobe von der Quelle stellt sich heraus, dass E-Coli im Wasser war. Die ganze Zitzenreinigung hat mit dem Colihaltigen Wasser stattgefunden. Sie haben die Stämme aus den Mastitiden mit den Stämmen im Wasser verglichen, die waren identisch. Eine wichtige Frage ist also: Gemeindewasser, oder eine eigene Quelle?

EXP: Wir haben einen Bestand mit Roboter. Da war die Wahrnehmung des Euterproblems leider etwas verzögert, da wir da seit Jahrzehnten nur eine reine Fruchtbarkeitsbetreuung machen. Der hatte sehr viele akute Viertel. Umgerechnet hatten 92 Kühe von 100 in einem Jahr eine akute Mastitis. Wir haben festgestellt, dass es im Kraftfuttersilo Schimmelbildung gab. Die Mykotoxine haben die Kühe dann so in ihrer Abwehr geschwächt, dass Umweltkeime heftige Mastitiden mit schweren Verläufen verursacht haben. Ausserdem hatte er Tiefstreu Boxen mit nassen Stellen, viele Fliegen, keine optimale Wasserversorgung, keine ordentliche Zwischendesinfektion (nur Spülen mit kaltem Wasser) und 5 alte Kühe mit chronischen Problemen. Das war dann auch der erste Schritt, sich von diesen zu trennen.

VET: Und bei Melkroboterbetrieben gibt es keine Melkreihenfolge.

VET: Habt ihr auch die Erfahrung mit Melkroboterbetrieben, dass die Landwirte sich die Tiere nicht mehr anschauen? Akute Viertel werden nicht mehr erkannt.

EXP: Die müssen dann lernen, wie man mit dem Alarm umgeht. Das bedeutet am Morgen die Alarmliste ausdrucken und in den Stall die Kühe anschauen gehen! Bei perakuten Coli- oder Klebsiellenmastitiden ist man da manchmal aber schon zu spät.

VET: Was ist eure Meinung bei der Einstreu. Ich habe einen Betrieb, der hatte Strohmatratzen mit Kalk und da viele Probleme. Jetzt separiert er die Gülle und streut dann die Feststoffe ein. Er meint er hätte weniger Probleme.

VET: Eigentlich gibt es beim separieren mehr Probleme.

VET: Wenn ich mir seinen Medikamentenverbrauch anschaue hat er auch Probleme.

EXP: Es sollte halt so trocken wie möglich sein. Es wird viel diskutiert wegen den Deckschichten, d.h. oben drauf noch eine trockene Schicht Häckselstroh aufzubringen. Wir haben einen

saisonalen Weidebetrieb, mit sehr guten Zellzahlen, der auch separierte Gülle einstreut. Allerdings ist der wenig mit anderen Vergleichbar, da die Kühe im Sommer der heiklen Zeit immer draussen sind

VET: Ich habe einen Betrieb, der benutzt Strohpellets und hat bis jetzt keine Euterprobleme.

VET: Habe ich auch einen Betrieb. Die sind so hart, dass mir die Knie weh tun wenn ich da Kalben muss. Und der hat viele Nabelentzündungen. Ich glaube das liegt daran. Nun ändert Landwirt das wieder. Die Strohpellets saugen sehr gut, sind dann halt aber auch feucht.

EXP: Grosse Oberfläche da kleine Partikel zusammen mit Feuchtigkeit ein Bakterienbrutgebiet.

VET: Die sind wahrscheinlich auch teuer.

VET: Wie ist es mit vermehrtem Kalken?

EXP: Man darf kein Chlorhaltiges Zitzendippmittel verwenden. Das gibt eine Interaktion. Und Jod mit wenig Glycerin trocknet die Zitzenhaut stark aus. Beim Kalkstroh kommt es auf die gute Mischung an. Generell sollte man auch alle Matratzenstreuarten gut belüften. Das ist aber Zeitaufwendig. Wenn es oben gut trocken ist, dann ist schon viel erreicht. Bei S. uberis hat man immer gesagt, dass man viel kalken soll. Aber den sehen wir ja auch bei den Landwirten, die kalken. Von Kalk aus Zuckerfabriken kann ich nur abraten. Da sind schon viele Keime drin

Einstreu sollte vor allen Dingen genug und trocken sein

VET: Ja man müsste glaub bis pH 9 kommen, damit es was bringt und das sind dann gewaltige Kalkmengen.

VET: wir haben einen Betrieb mit Sandboxen, der hat eine gute Eutergesundheit. Aber der ist auch sonst ein toller Betrieb.

EXP: Habt ihr Betriebe mit Kompoststall?

VET: Ja, hatte einen, der hat aber gewechselt.

VET: Wir haben einen, der zuerst nur Kompost hatte und jetzt noch Stroh oben drauf. Bis jetzt keine Probleme.

VET: Wir hatten einen, der hat es aber nicht trocken bekommen.

Feedbackrunde

VET: Wir haben alle die gleichen Probleme und probieren eine Lösung zu finden

VET: Es gibt kein Patentrezept

VET: Es ist individuell, von Landwirt zu Landwirt verschieden

VET: Alle kochen mit Wasser, auch Exp. Wir versuchen es anzugehen und schauen dann

VET: Ich muss mir mehr einfallen lassen, dass ich auch auf die Betriebe komme. Brauche da einen Ansatzpunkt.

VET: : Du könntest ja eine Aktionswoche anbieten? Aber das ist wirklich schwierig bei dir.

VET: Und es ist schwierig heraus zu finden, woran es liegen könnte. Habe da zu wenig Erfahrung. Mir gehen die Ideen aus

VET: : Das hab ich auch gedacht, als ich Vet gehört habe. Die hat eine Ausbildung. Ich habe im Team jemanden der das kann. Wenn ich nicht mehr weiter komme, schicke ich sie.

Zustimmung, dass wenn man an seine Grenzen kommt, an jemand anderen weiterverweisen sollte.

Kann ich mich selber in die Melktechnik soweit rein arbeiten, dass ich das komplett verstehe?

Wenn du ein Freak bist, ja!

EXP: Melken- beobachten muss man trainieren. Aber man lernt es: Vor allen Dingen auf die Kühe schauen: Wie benehmen sie sich? Das gibt viel Info darüber ob auch was mit der Maschine nicht stimmen könnte.

VET: : Wir habe alle die gleichen Problem. Wenn ich nicht mehr weiter komme hole/frage ich Experten

Protokoll Kommunikation

Experte stellt sich vor und gibt das Programm bekannt

- Übungen:
  - Merkstrategien: 20 Buchstaben merken 15s lang und danach wiedergeben: Stichwort „structure and remember“
  - Interpretation eines Bildes von einer Salzmühle: zeigt auf, dass gewisse Sachen sehr unterschiedlich gesehen werden so auch in der Kommunikation schnell Missverständnisse auftreten können.
  - 2 Situationen als Einstieg in die Kommunikation:

1. Roboterbetrieb mit massivem Salmonellenproblem- was mache ich als Tierarzt?
- Vorschläge: mehr Infos erfragen um die Situation besser managen zu können,
2. Unzufriedener Kunde, da Verspätung
- Vorschläge: proaktiv handeln von unterwegs anrufen, dass man verspätet ist (mehrere Teilnehmende)
3. Kuh mit chronischer Gebärmutterentzündung-Besitzer meint „das sagt ihr immer wenn ihr nicht wisst, wo das Problem liegt“
- Zurückfragen: was denkst Du denn, wo das Problem liegt?

Experte erläutert die Tabelle mit den **verschiedenen Rollen, die man in der Kommunikation einnehmen kann** (siehe Fotoprotokoll):

- Wissender
- Experte/Expertin
- „Hebamme“ (VermittlerIn)
- Coach

Situation mit Mistgabeldrohung

- Deeskalieren: VET schlägt vor, eine Frage zu stellen, damit das Gegenüber aus dem Adrenalinrausch herauskommt
- Tip: 3 x probieren, wenn es nicht geht verschwinden.

Exp erläutert **typische Gesprächstypen:**

1. Beratungsgespräch: Fragen>Zuhören, Problem definieren und präzisieren, Konsequenzen erörtern, Verständnis zeigen, konkrete Lösungen mit einem Fahrplan erarbeiten, Resultat überprüfen-nachfragen
    1. Zeitaufwändig!

2. Kritikgespräch: Ist-Zustand klar benennen, Ich-Aussagen machen, lösungsorientierte Optionen mit Einbindung des Gegenübers anbieten, eigene Wünsche, Vereinbarung schliessen

Exp erläutert das **Harvard Konzept:**

1. Totschlagargument vermeiden
2. Sachverhalte und Personen trennen!
3. Offene Alternativen:
   Neutrale Beurteilungskriterien heranziehen (z.B. Gesetze)
4. Beste Alternative wählen
5. Emotionen verbalisieren und Themenbezug immer wieder herstellen
6. Ziele und Konsequenzen aufzeigen
7. Wenn nötig vertagen

Erfahrungen austauschen:

1. VET: Bauer mit 50 Milchkühen ohne Hilfe hat ein Mastitisproblem und ist überzeugt von Homöopathie, er beklagt sich, dass Tierarzt davon nicht viel versteht und holt aber gleichzeitig ab und zu bei Vet Antibiotikatübli….
    - VET hat bereits versucht das Problem im Detail zu erklären, dass eben nicht alle Erkrankungen mit Homöopathie zu heilen sind, der Tierhalter meint damit beschwöre er/sie das Unglück herauf….
    - Der Tierhalter ist vermutlich überfordert und sucht einen Sündenbock….
    - Wir brauchen mehr Infos: wie ist dann konkret der Therapieerfolg mit H.? Gibt es andere Berater auf dem Betrieb? Was ist deren Meinung?
    - Gesprächsart? Kritikgespräch, Tierhalter ist den Argumenten nicht mehr zugänglich, Therapiegespräch: konkreter Kügeliplan machen wie eine kleine Studie. Interesse an Homöopathie zeigen, ihn um Infos bitten.
    - Wichtig: nicht die Nerven verlieren und Vertrauen schaffen, dann kommen die wahren Gründe für das Verhalten automatisch ans Licht.

2. VET: Therapiemisserfolg kommunizieren obwohl man alles gemäss good veterinary practice gemacht hat….
    - Nicht auf die Wippe geraten: Argument-Gegenargument, bringt das Ganze nicht weiter und ist Nervenzehrend
    - Fragen! „Was machen wir jetzt sinnvollerweise?“ Was hilft der Kuh? Gemeinsame Lösung vereinbaren¨!
    - Prävention: Bei Therapien immer gemeinsame Entscheidungen treffen!

Feedbackrunde

- viel Theorie
- muss man erst mal sacken lassen, gute Impulse bekommen
- evtl. wäre es besser, wenn man die Sachen eindeutiger/überspitzter darstellt
- zu kurz, aber sehr gut, spannend
- super Präsentation, es war gut die Fälle zu besprechen
- sehr gut, hoffe ich profitiere davon
- fast ein wenig zu viel
- viel Theorie gelernt, diese ist breiter als gedacht, Zeithorizont um das Gespräch zu führen
- fremde Materie, sehr spannend, automatisch macht man schon vieles richtig, aber es wäre spannend mal was anderes zu probieren
- Die "Wand" ist so voll, habe mich ein wenig überfahren gefühlt, man macht vieles schon richtig, ansonsten würde es auch gar nicht funktionieren
- Gut

Protokoll alternative Therapien

Was sind eure Erfahrungen mit alternativer Therapie?

Exp: Bitte Komplementär-Medizin sagen. Nicht alternative Medizin, da es eine Ergänzung ist!

VET Selber keine Erfahrung, wird aber nachgefragt, überlege, wie ich zu dem Wissen kommen kann.

VET Keine Erfahrung

VET: wenig Erfahrung, eine meiner Assistentinnen interessiert sich für das Thema

VET: Habe mal einen Homöopathie Kurs gemacht, aber vertraue dem Ganzen nicht wirklich, wende es nicht an.

VET: Mache Ausbildung in Phytotherapie, bei uns im Praxisgebiet ein grosser Boom, möchte dazu auch das Wissen haben.

VET: Wird von Landwirten selber durchgeführt. Mein Chef macht auch Homöopathie und Akkupunktur, bestimmte Kunden fragen dann spezifisch nach ihm. Wir überlegen, wie wir dieses Wissen in der Praxis halten können.

VET: Bei uns fragen viele nach homöopathischer Behandlung. Ich habe keine Erfahrung.

VET Viele Nachfragen in der Praxis, haben Landwirte, die auch homöopathische Mittel bei uns holen. Aber haben keine Ahnung zur Heilungstendenz. Wir wenden selber keine komplementären Methoden an.

Expertenrunde

I. Wie starte ich mit der Homöopathie am besten, welche Bereiche eignen sich besonders?

   - Rund um die Geburt, da fallen die verschiedenen Kuhtypen nicht so ins Gewicht, da die Geburt nach „festgelegtem Ablauf“ stattfindet. (Geeignete Mittel siehe PDF Exp Folie 7)
   - Einzeltierprobleme wie Saugschwäche bei Kälbern bieten sich auch an (Geeignete Mittel siehe Folie 8 PDF Exp)
   - Komplexe Fälle und Gruppenprobleme eher schwierig

Klassischerweise geht das doch eigentlich nicht, immer das gleiche Mittel für gleiches Problem anwenden?

Exp: Das ist korrekt für chronische oder sehr schwere Erkrankungen. Bei Geburt herrscht ja in dem Moment eigentlich immer das gleiche Problem vor.

II. Konstitutionsmittel: wie weit können sie einen Patienten z.B. bei chronischer Mastitis unterstützen?

Konstitutionsbehandlung ist die „klassische" Homöopathie. Da werden die 12 verschiedenen Kuhtypen (Charaktere) mit in Betracht gezogen (Folie 9 PDF Exp) je nach Typ kann dann das passende Mittel verabreicht werden. Bei Chronischen Mastitiden kann die Abwehr der Kuh unterstützt werden, dauert aber etwas. Animation zur Selbstheilung. Die Kuhtypen mit den gezeigten Bildern sind gut beschrieben (Homöopathische Konstitutionstypen beim Rind, Angela Lamminger-Reith)

Einzeltierbehandlungen :

- Akutbehandlung: nach dem Symptom gehen
- Chronisches Leiden: Konstitutionsbehandlung

Gruppenbehandlung:

- nach klinischen Symptomen behandeln (Komplexmittel)
- Von Wesselmann gibt es für die Bestandesbetreuung gute Homöopathische Zusammensetzungen.

III. Wie viel Evidence gibt es für die Wirkung von Homöopathischen Mitteln?

Exp: es gibt meines Wissens nicht viel klassische Studien über Homöopathie, in meiner Erfahrung wirkt es. Ob es jetzt der Inhalt der Kügeli ist, oder die Begleitmassnahmen weiss ich auch nicht.
Da ja immer verschiedene „Charaktere" mit unterschiedlichen Krankheiten und somit unterschiedlichen Mittel behandelt werden, ist das sehr schwer abzuklären!
EXP Ich habe mal 2 Metaanalysen rausgepickt. Das Problem ist, dass es kaum randomisierte (d.h. zufällige Patientenauswahl) und verblindete Studien gibt, die zu einer Metaanalyse zusammengefasst werden können. Eine leichtgradig bessere Wirksamkeit von Homöopathischen Mitteln gegenüber von Placebo beim Menschen (OR ca. 2) werden in der einen Studie angegeben. Eine weitere Studie besagt, dass es sehr auf die Indikation der Anwendung ankommt, so waren homöopathiscje Behandlungen im Falle eines postoperativen Ileus beim Menschen nicht sehr erfolgreich, allerdings gibt es im Bereich von Allergien Erfolge zu verzeichnen. www.wisshom.de
Mir ist die Idee gekommen, dass der sogenannte personalised Medicine Ansatz aus der Humanmedizin eine Möglichkeit wäre, homöopathische Behandlungen zu untersuchen. Da will man eben keine Mittelwerte oder Mediane vergleichen, mit dem Argument, dass jeder Patient „einmalig" ist. (NZZ Artikel zum Mitnehmen oder hier: https://www.nzz.ch/wissenschaft/medizin/klinische-studien-am-einzelnen-patienten-1.18649038).

IV. Gibt es standardisierte Behandlungsstrategien?

a. Grundsätzlich individualisierter Behandlungsansatz, Unterschied Behandlung akute und chronische Leiden (s. oben)
b. Mit Komplexmittel (XYZ) arbeiten, ist aber nicht der klassische Ansatz

c. Es gibt ein digitales Repertorium, das die Arbeit erleichtert beim Aufarbeiten der Fälle und dem Therapieentscheid, somit gibt es da einen Standard. (Programm heisst RADAR).

Kann man alle Mittel kombinieren?

Exp: Nein, es gibt Mittel, die man nicht kombinieren kann, da sie sich aufheben oder sich beeinflussen.

Wenn ich voll daneben liege mit meinem Mittel, mach ich dann was „kaputt"?

Bei einer einmaliger Gabe eigentlich nicht.

Es wird immer von einer initialen Verschlechterung berichtet bis es bessert, wie ist das zu erklären?

Exp: Die Kuh macht frühere Krankheiten noch mal im Schnelldurchlauf durch Stärkung des Immunsystems.

Wie wichtig ist der Erreger für die Homöopathie?

Exp: eher untergeordnet, es geht um das Immunsystem als Ganzes.

V. Ich habe Kunden der verwendet Phytolacca vet.comp, Phytolacca americana C30, Acidum silicicum C 30, Carbo vegetabilis C200, Streptococcinum C30, Asa Foetida C6, Sulfur C30, Staphylococcinum C30 für die Herde übers Trinkwasser oder Einzeltierbehandlung 3 ml / Tier 14 Tage lang gegen Mastitisprobleme, macht das Sinn?

Exp: Eher nicht sinnvoll (eigentlich 5-6 Mittel maximal laut Wesselmann). Und die Zusammensetzung macht auch nicht 100% Sinn ausserdem sind da noch Nosoden (Streptococcinum C30, Staphylococcinum C30) drin. Nosode selber machen, wäre wahrscheinlich sinnvoller. Ich habe keine gute Erfahrung mit Standardnosoden gemacht. Die Verabreichung über den Trinkbrunnen ist abzulehnen. Homöopathie ist nicht nur als billige Methode ohne Absetzfirst zu sehen!

Generell wichtig bei solchen Fragestellungen, dass Tierarzt und Homöopath sich mit dem Tierhalter zusammen um das Problem kümmern, Gegeneinander ist immer kontraproduktiv.

Lapis albus gilt als „Zellöffner" ist im Phytolacca als D Potenz drin, müsste über 14 Tage verabreicht werden.

Silicea leitet aus. Kann man einsetzen, wenn Milchprobe immer steril bei chronischen Mastitiden 3-mal alle 24 h).

Wie ist das mit „Konzentration" von homöopathischen Miteln:

Exp: Grundsätzlich kann man sagen, das man D6 14 Tage geben kann und C200 nur einmal.

VI. Hofhomöopathieapotheke mit Mittel gegen einzelne Symptome und nicht gegen das ganze Krankheitsbild, ist das nicht im Wiederspruch zur klassischen Homöopathie?

Exp: Ich würde die Stallapotheke von Omida mit dem zugehörigen Buch empfehlen, das ist sehr gut dokumentiert und die wichtigen Punkte sind miteinbezogen. Wichtig ist die Unterscheidung Akutbehandlung und andere Behandlungen. Bei Akutbehandlungen sind Lokalsymptome wichtiger als die Gemütssymptome, deshalb nicht zwingend im Widerspruch zur klassischen Homöopathie.

Kann man Homöopathie zusammen mit Schulmedizin verwenden?

Exp: Das kann man sehr wohl, es soll ja die Selbstheilung anregen.

VII. Kurse, die empfohlen werden können?

Exp: Ig Homöopathie: Sehr empfehlenswert und seriöse Kurse zur Stallapotheke. Ausbildung bei Camvet auch empfehlenswert.

VIII. Darmmilieu und Mastitistherapie, was ist da dran?

Exp: Effektive Mikroorganismen (EM) hab ich auch schon mit Erfolg eingesetzt. Es gibt Forschung aus der Humanmedizin im Zusammenhang mit Mikrobiologischem Priming, das Kindern aus Kaiserschnittgeburten fehlt, da sie die Scheidenflora der Mutter nicht mitbekommen haben.

In der Bioresonanz arbeiten wir mit den Schwingungen von und versuchen diese in den richtigen Takt zu bringen, ich weiss nicht wie, aber es hat einen Effekt.

Was ist wenn Landwirt mich anruft: Jetzt habe ich das homöopathische Mittel XY gegeben und jetzt hat Kuh Fieber?

Exp: Fieber an sich nicht schlimm! Den Allgemeinzustand anschauen.

Thymian im Zusammenhang mit Darmmilieu?

Exp: Ist altbekannt als Darmregulierendes Kraut, deshalb auch in vielen Würsten;)
VET: Thymianöl wirkt gegen Varroamilben bei den Bienen!

IX. Trank zum Trockenstellen?

EXP: Ist Produkt von NAVETA AG, enthält Pfefferminz und Salbei, auch bei Frauen zum Abstillen empfohlen. Bewirkt Milchrückgang.

Feedbackrunde

VET: das Ganze ist sehr komplex, braucht fast ein 2. Studium, kann ich momentan nicht machen, da fehlt die Zeit.

VET: Bei der Kundschaft eher variable Erfolge, Kurs zur Stallapotheke sinnvoll, um besser auf die Kundschaft eingehen zu können. Persönlich werde ich nicht zum Homöpath, hat lange genug gedauert ein anständiger Schulmediziner zu werden, aber unterstütze Mitarbeitende.

VET Fallauswahl wichtig, habe aber nach wie vor kein Interesse, werde aber etwas offener sein in den Diskussionen.

VET Die Berichte über Bioresonanz haben mich „beeindruckt“, möchte offen bleiben für Diskussionen und Bauern ernster nehmen im Bereich komplementäre Methoden.

VET: Fand es sehr spannend und sehr gut, dass jemand da war, die das täglich auch in der Praxis anwendet.

VET: Für mich war v.a. der Unterschied akut und chronisch wichtig und die verschiedenen Kuhtypen, kann mir eine Weiterbildung in Richtung Komplementärmedizin vorstellen.

VET: Schwanke noch etwas wie ich die Methoden beurteilen soll, bei den Kindern schon erfolgreich Homöopathie eingesetzt. Sicher weiterhin offenbleiben für Diskussionen mit den Kunden!

VET Wichtig die Diskussionen weiter zu führen und auch Grenzen der komplementärmedizinischen Behandlungen aufzeigen.

Protokoll Melktechnik

1. **Erfahrungen austauschen**

- Beratet ihr eure Landwirte hinsichtlich Melkarbeit, Technik
- Glaubt ihr genügend Erfahrung zu haben hinsichtlich der Beratung?
- Was glaubt ihr hat den grössten Einfluss auf die Eutergesundheit bei der Melkarbeit/Technik?

Statements:

VET: Melkarbeit berate ich bei der Technik fühle ich mich unsicher, Offensichtliche Fehler sehe ich aber auch.

VET: Wenn offensichtliche Probleme z.B. mit der Hygiene da sind, berate ich selber sonst Melktechniker

VET: Melkarbeit berate ich, frage immer nach Service und Wechsel der Zitzengummis, Melkarbeit verbessern immer erster Schritt. Ich sage ihnen immer, dass ich genau hinschaue weil er jetzt ein Problem hat, Problem = wir müssen was ändern.

VET: Landwirte wollen nicht immer Melkbesuch, der Melkberater kommt wenn Abzüge, habe persönlich von der Melktechnik zu wenig Ahnung.

VET: Berate nur, wenn sich die Tierhalter beraten lassen wollen, mache dann ab, zum Schalmtest machen und komme dann etwas zu früh, so kann ich sozusagen inkognito die Melkarbeit beurteilen, ohne, dass der Bauer es mitschneidet. Beim CMT kann ich dann auch Zitzenkondition beurteilen.

Frage immer nach dem letzten Service und was sie da gefunden haben. Oft bringt es etwas wenn der Techniker kommt und nachmisst. Für Bauer immer einfacher wenn die Technik schuld ist.

VET: Melkarbeit berate ich auch, Melktechnik nur wenn systematische Probleme bestehen z.B. Entzündungen immer am gleichen Viertel etc. Wichtigkeit der einzelnen Bereiche von Fall zu Fall unterschiedlich.

VET: Melkarbeit überprüfe ich auch, Melktechnik wenn ich denke es ist ein Problem vorhanden. Melkarbeit und Hygiene erfahrungsgemäss grössten Einfluss.

VET: Man gibt schnell der Technik Schuld, aber wahrscheinlich sind es viele andere Faktoren, die ein Mastitisproblem mit beeinflussen

Frage Exp Welche Indikatoren habt Ihr den konkret, damit Ihr empfehlt, die Technik abklären zu lassen? Zitzenkondition schlecht
Viele subklinische Viertel mit unterschiedlicher Ursache
z.B. wenn immer Frischgekalbte betroffen sind
Plötzliche Katastrophe
Werden nicht leer

Expertenrunde

- Experte stellt sich kurz vor.
- Gründe für Maschinenabklärung aus seiner Sicht:

  - Kühe werden nicht leer
  - Plötzliche ZZ Erhöhung
  - Verändertes Verhalten der Kühe (Unruhe)
- Melkarbeit sehr wichtig, moderne Anlagen lassen sich an den Melker anpassen;)
- Beim Melken möglichst nicht stören ist wichtig.
- Gute Anlagen sind:
  - Korrekt installiert (Isonorm)
  - Vermeiden von Über-oder Unterdimensionierung der Leitungen 50-60 Zoll; 75 Zoll Leitungen sehr stabiles Vakuum aber es braucht viel Power zum reinigen!
  - Gefälle bei hochverlegten Leitungen muss stimmen 05%, keine Knicke oder Brüche, wenig Krümmungen
- Nicht aufgrund von Eindrücken entscheiden sondern Maschine ausmessen lassen!
- Wichtigstes Teil ist das Melkzeug, da direkt mit der Kuh in Kontakt:
  - Gewicht (nicht zu leicht klettern einfacher, nicht zu schwer wegen Belastung der Zitzen)
  - Lufteinlass durchgängig: 1.1 mm kann mit einem 1 mm Bohrer überprüft werden!
  - Zitzengummi
  - Vakuummessung im Melkzeug gibt manchmal Aufschluss über Probleme
- Lärm: max. 70 DB, soll angenehm sein v.a. auch für Melker sonst wird der nervös!
- Vibrationen v.a. bei Melkständen auf dem Rohrsystem: Kühe sehr empfindlich 0.3-0.4 m/s^2 max.
- Milchflusskurve ist sehr hilfreich beim Erklären von Problemen z.B. Blindmelken
- Kriechstrom: Schwieriges Thema
  - Q ist sehr empfindlich aber individuelle Unterschiede
  - Verdacht bei: stark schwankenden ZZ, wechselndem Ausmelkgrad, Wetterfühligkeit, hohe ZZ sterile Proben, bessere Melkbarkeit wenn Standeimer benützt wird
  - Handystrahlung und Magnetfelder eher kein Problem
  - Wer macht Messungen?
    - J. Doppmann ZMP, Max Waldburger Bamos AG, Fredy Krummen (unabhängig)
- Kugelventil-Melkzeug von GEA: keine Gefahr von „falscher Luft“ beim Anhängen, weil bei jedem Eingang ins Sammelstück ein Kugelventil ist.
- Multilaktor: System ohne Sammelstück, Lufteinlass am Zitzengummi auch beim Roboter, bei Happel und beim Milkrite Melkzeug
- Bei den meisten Herstellern Vakuum an der Zitzenspitze konstant; bei Happel und Biomilker Entlastungsphase + Vakuumabbau => wenn die Zitzengummis alt sind funktioniert das System nicht mehr.
- Gleichtaktsysteme: Vakuumabfall an Zitzenspitze in Entlastungsphase durch die Milch im Sammelstück, funktioniert aber auch nicht immer.
- Wiederverwendung des Laugenwassers: Nicht empfohlen wird als „Stapelreinigung“ bezeichnet
- Einfache Beurteilung durch Praktiker?
  - Tests nach Spohr nicht sehr aussagekräftig
  - Sind auch nur statische Messungen
- Zitzengummis:
  - Schwarzgummi und Silikon

  - Schwarzgummi = Legierung, 2500 Melkungen ca. 6 Mt Lebensdauer, mehr Verschleiss bei Peressigsäurezwischendesinfektion
  - Silikon: Wechsel all 5000 Melkungen, mehr Hyperkeratosen wenn Vakuum hoch da Silikon weich, Vakuum deshalb 2 kPa tiefer stellen; nehmen Fett auf
  - Runde Köpfe grosse Öffnung: Grosse und lange Zitzen auch gut für nicht gerade Euterboden, wenn Zitze zu kurz und zu dünn = Haftprobleme
  - Flache Köpfe: Kurze Zitzen, gerade Euterboden, eher Einschnüren bei gröberen Zitzen und Stufeneutern
  - Kompromissmodell mit runder Lippe und eher kleiner Öffnung
  - Schaftdicke auch entscheidend für Haften
  - Wahl: am häufigsten 21 mm Öffnung, Schaftdicke anpassen
  - 3-eckiger Zitzengummi: funktioniert nur mit kurzen Zitzen
  - Zitzenbecher: Metall besser als Plastik stabiler, schwerer und weniger anfällig für Risse
  - Klettern der Zitzenbecher: Zu grosser Schaft, nasse Zitzen beim Anhängen, nicht genügend angerüstet
- Vakuumprobleme:
  - Standeimer einfach eingeschlauft in Rohrmelkanlage: zu hohes Vakuum da Verlust des Milchtransports nach oben wegfällt! Gibt ein spezielles Teil dazu, das dies reguliert.
    - In Melkständen kein Problem, da nach unten gemolken
- Anrüstautomatik:
  - Im Gruppenmelkstand (side by side und Fischgeräte) unter Schweizer Verhältnissen Nein, Rhythmus finden ca. 4 Kühe vorbereiten, dann zurück gehen und anhängen
  - Doppel12 er side by side oder 2 x 4 Tandem: Ja, erleichtert das Management
  - Zeitgesteuert besser als Milchflussgesteuert
  - Besser zu lange anrüsten als zu kurz
  - Gewisse Automaten pfeifen, das stört die Kühe
- Problembetrieb stellt auf Roboter um
  - S. uberis Problem: chronische Tiere ausmerzen…
  - Stress beim Angewöhnen ist immer da: 4 d nur als Kraftfutterautomat brauchen, damit die Kühe reingehen
  - Zwischendesinfektion sicher zu empfehlen
  - Saubere Kühe und saubere Euter: Euterhaare abflammen!
  - Möglichst wenig an Einstellungen schrauben
- Separierte Gülle als Einstreu?
  - Muss schnell trocknen dazu braucht es gute Durchlüftung des Stalles
  - Bakteriell belastet aber auch nicht pathogene Bakterien => Gleichgewicht
  - Besser als man denkt
- Kalk-Strohmatratze
  - Kohlesauren Kalk verwenden! Stroh nachstreuen
  - Im Anbindestall mit Strohhäksel, nur Kalk auf Gummimatten reizt die Haut zu stark
  - Verträgt sich schlecht mit Chlorhaltigen Zitzendippmitteln (Inaktivierung und Austrocknung der Haut)
- Melkarbeit:
  - Vormelken
  - Putzen: Schaumreiniger haben sich gut bewährt, pflegen die Zitzenhaut
  - Feuchttücher: cave spröde Haut

- Schaumbecher nach jedem Melken waschen!!
- Rhythmus finden 1 min Anrüstzeit als Richtwert!
- Lufteinbrüche beim Anhängen vermeiden
- Ausrichtung der Melkzeuge korrekt machen, Servicearme einbauen falls nicht vorhanden; side by side problematisch
- Ausmelken sollte nicht nötig sein

2. Feedbackrunde

Alle wünschen sich einen Betriebsbesuch mit Experte wird auf April vorgesehen wenn nicht mehr so kalt

Vet: So viel Notizen wie noch nie!

Vet: Service gemacht heisst nicht dass auch alles einwandfrei funktioniert, wenn die Kuh an der Maschine hängt

Vet: Beobachten ist oft wichtiger als messen! Oder zuerst beobachten und dann bestätigen mit messen.

Vet: Problem mit Vakuum, wenn Eimer an der Rohrmelkanlage

Protokoll Trockenstellen

a. **Erfahrungsaustausch**
   a. Bei wie vielen Betrieben stelle ich schon selektiv Trocken (in Prozent)?
   b. Bei wie vielen Betrieben funktioniert das gut (in Prozent)?
   c. Was sind die häufigsten Probleme, die ihr seht?

VET: Bei uns macht das keiner. Einige haben es auf eigene Faust versucht, jedoch ohne es mit uns abzusprechen oder uns zu informieren. Die sind gescheitert. Haben viel negatives Feedback von denen bekommen. Bei einem einzigen hat es gut funktioniert, der hat aber aufgehört.

VET: Bei uns gibt es keine Alternative mehr. Punkt. Die kommen und man bespricht, es gibt keine andere Wahl. Dadurch habe ich auch Kunden verloren. Der Orbeseal Verbrauch ist bei uns gestiegen. Wir sind auch auf Wiederstand gestossen. Bei einem müssen wir eine Halb-Lösung machen, damit wir ihn nicht verlieren.

VET: 80% stellen bei uns selektiv trocken, ein paar kommen ganz ohne antibiotische Trockensteller (AB-TS) aus, und manche stellen immer mit AB trocken. Manche kommen mit Liste und fragen ob man helfen kann und dann schauen wir uns die Zellzahlen (ZZ) zusammen an. Bei uns in der Praxis sind AB-TS runter und wir verkaufen mehr Orbeseal. Bei ein paar Mastbetrieben ist es schwer, weil die zukaufen, da geht es fast nicht ohne AB

Exp: Die kann man eigentlich nicht ohne AB Trockenstellen. Das ist klar.

VET: Am Anfang mussten wir viel diskutieren! Jetzt sind LW immer mehr aufgeklärt. Geht immer besser.

VET: Der grösste Teil stellt bei uns selektiv trocken. Wir setzen AB-TS nach Auswahlverfahren ein. Orbeseal ist bei uns auch rauf. AB-TS vielleicht runter? Weiss ich nicht. Das funktioniert bei uns eigentlich Tip Top, Am Anfang viel geredet, viel diskutiert und dann zusammen eine beste Lösung gefunden.

VET: Vorher haben wir schon selektioniert. Jetzt selektionieren wir bei AB-TS noch mehr. Vereinzelte machen nicht mit, das sind meist die älteren LW, die jungen kommen schon von alleine. Manche bei uns haben von selber abrupt aufgehört mit AB-TS. Die hatten dann vermehrt Gust-Viertel und waren hässig deswegen. Bei uns ist der Orbeseal Verbrauch auch gestiegen.

VET: Ich habe noch nichts gemacht und lass sie trocken stellen wie sie wollen. Aber ich gebe schon weniger AB-TS ab. Ich hab auch LW, die es selber gemacht haben, das hat nicht funktioniert. Und mit manchen habe ich schon diskutiert.

Exp: Du kannst also nicht sagen, dass du noch nichts gemacht hast. Diskutiert hast du schon.

VET: Ja, diskutiert habe ich schon. Meine LW überlegen auch selber, aber jemanden, der schon immer mit Biclox trockengestellt hat und die Kühe eine hohe Leistung haben, da mach ich nicht rum, sondern verkauf lieber Bicolx. Ich warte mal den AK heute ab.

Vet: Ich habe am Anfang einen Infoabend für die Landwirte gemacht. Zur Sensibilisierung. Das hat gut funktioniert. Viele machen gut mit und stellen selektiv trocken. Ein paar von denen haben Hochleistungskühe, da funktioniert das nicht gut, aber das Problem ist, dass die schon nicht gut die Kühe aufs Trockenstellen vorbereiten. Ich habe auch Kunden fast verloren. Und bei 2-3 trau ich mich nicht mit dem selektiven Trockenstellen. Die Kühe haben zu hohe Leistung und sind Zuchtschaukühe. Bei mir gibt es keine Grosspackung mehr. TS gibt es nur mit Namen und Grund. Die Richtung stimmt. Bei ein paar Betrieben funktioniert das mit dem Orbeseal nicht gut. Ich bin sicher runter gegangen mit dem Verkauf von AB-TS.

VET: Wie ist das, ist da immer jemand da, wenn der Landwirt kommt zum AB-TS holen? Schaut ihr euch das an?

VET: Bei uns ist immer jemand vorne und man kennt ja seine LW. Generell bin ich mir beim selektiven Trockenstellen nicht sicher, ob das das Ende der Fahnenstange ist. Die AB-TS gehen zwar runter, aber die Injektoren für Mastitiden dann vielleicht rauf?

Exp: Mir geht es wie euch allen. Die Idee ist mit der Änderung der TAMV gekommen. Das war eine politische Entscheidung. Ich hatte am Anfang Sorge, dass sie die AB-TS ganz verbieten werden, wie in Holland. Das wäre eine Katastrophe. Ich bin VET Meinung, dass die AB-TS evtl. runtergehen, aber die Injektoren für Laktationsbehandlungen steigen. Beim TS gibt es eine bessere Heilungsrate, keine AB-haltige Milch. Bei Betrieben mit grenzwertiger Eutergesundheit kommen dann die Probleme, da bin ich eigentlich eher dafür, dass man mehr AB TS einsetzt.

b. Expertenrunde

Exp: Ich habe die Eutergesundheitsdaten von 5 Betrieben mitgebracht (s. Folien v. Exp)

VET: Frage zu Betrieb 3. Ist das ein Betrieb, wo Haltung und Fütterung gut ist?

Exp: Das ist ein Roboterbetrieb mit einer Durchschnittsleistung von 12000kg und der Bauer hat die Einstellung Wenn meine Kühe mit zu hoher Leistung zum TS gehen, dann habe ich die Kühe zu früh besamt. Eigentlich hat der Betrieb zu wenig Fressplätze.

Exp: Generell kann man sagen, dass es vermehrt zu Vierteln am Anfang der Laktation führt, wenn man selektiv trocken stellt.

VET: Kannst du einen Vergleich von den verkauften Antibiotika machen. Also vor dem selektiven TS im Vergleich zu nach dem selektiven TS.

Exp: Das Problem sind da die fehlenden Daten……

Exp: Akute Viertel hast du immer, jedoch sind S. uberis ohne AB-TS mehr vorhanden.

Viele: Zustimmung

Exp: Ich würde raten S. uberis und S. aureus Betrieb nicht selektiv trocken zu stellen. Grangeneuve hat nun mehr klinische Viertel. Aber die sind am Anfang auch von 100 auf 0 und waren strategielos.

VET: Diese Betriebe haben bei uns dann schon wieder mit AB Trockengestellt.

Exp: Grangeneuve ist ein Kantonsbetrieb, da kommt mehr Druck von oben. Daher sind die nicht wieder auf 100% Antibiotika zum Trockenstellen zurück, sondern stellen nun selektiv trocken.

Ich würde Tiere, die im Sommer raus kommen mit Penicillinen TS, die Weidemastitis mit T. pyogenes scheint da wirklich ein Problem.

Man muss auch immer das Keimspektrum des jeweiligen Betriebes berücksichtigen und das individuelle Management. Manche machen ja ihr Management eher spontan. Um eine Kuh vernünftig Trockenzustellen muss man sie gut vorbereiten. Und Landwirte haben z.T. immer noch den Irrglauben, das mit AB-TS die Milch zurückgeht.

VET: Ich sehe das grösste Potential bei der Haltung von Transit Kühen.

VET: Ja, die Haltung von Galtkühen ist wichtig. Die Weide finde ich da von der Hygiene am besten.

Viele: Zustimmung

VET: Bei grossen Herden geht es nicht unbedingt jeder Kuh gleich gut.

Exp: Wenn über 20% hohe ZZ am Ende der Laktation haben, dann gibt es einen Major-Keim und die Zukunftsempfehlung wäre AB-TS.

VET: Was ist die Grenze für hohe ZZ?

Exp: Wenn 20% ZZ über 150.000-200.000 haben, dann kann es auch ein Problem mit minor Keimen wie CNS sein, wenn jedoch die ZZ von 20% der Kühe zwischen 250.000 und 300.000 liegt, da gibt es einen Major-Keim.

VET: Nur nochmal zum Verständnis, bei den Beispielbetrieben waren die weissen Felder die Kühe, mit niedrigen ZZ, die nicht antibiotisch trockengestellt wurden?

Exp: Ja

VET: Und in Prozent? Wie viele stellen ohne Antibiotika trocken?

Exp: Wir haben keinen Betrieb der unter 50% der Kühe antibiotisch trocken stellt.

VET: Schade, dass man das nicht besser auswerten kann.

Alle: Zustimmung

VET: Hast du erfasst wie viele AB du verbraucht hast im Vergleich zu AB-TS?

Exp: Das wird in Zukunft ausgewertet werden.

VET: Meine Taktik ist abwarten

VET: Ja, das ist sicher nicht schlecht

Exp: Von Seiten der Behörden ist es ja, das ungesehenes AB-TS falsch ist, jedoch ist anschauen und zusammen diskutieren und dann AB-.TS richtig.

VET: Man erreicht die Bauern ja auch besser mit dem Diskutieren. Bei mir gibt es nicht unbedingt mehr Gustviertel.

Alle: Zustimmung

VET: Bei manchen Betrieben habe ich keine Gustviertel mehr.

Exp: Da spielen auch immer mehrere Faktoren eine Rolle.

Die Milchmenge beim TS ist entscheidend. Jedoch ist das Management für die Galtkühe dann auch mehr Arbeit, die Routine muss geändert werden und das ist schwer.

VET: Was empfiehlst du da?

Exp: Nährstoffdichte muss runter, Stroh einmischen. Bei grossen Betrieben funktioniert das z.T gut, da die die Kühe selektionieren und in eine separate Gruppe tun.

VET: Jedoch ist das z.T. schon ein Problem. Evtl. funktioniert das in einem Anbindestall doch besser?

Exp: Evtl. ist es da einfacher zu handhaben, aber ich habe die Erfahrung gemacht, dass es auch in grossen Betrieben gut funktioniert.

VET: Die Gustkühe drin lassen wäre auch eine Lösung

Exp: Da ist zwar mehr Aufwand, aber geht auch.

VET: Alles was mit Zwang kommt ist schlecht. Was braucht eine gute Eutergesundheit. Im Zuge der Infoveranstaltung für meine LW haben wir auch Fotos gemacht in den Betrieben. Die Bauern haben dann ihre Ställe wiedererkannt und das hat zum Aha-Effekt geführt. Seitdem wird mehr Zitzentauchmittel benutzt, es wird mehr mit Handschuhen gemolken und die Becher sind sauberer. Die Infoveranstaltung hat sensibilisiert.

VET: Bei klinischen Vierteln mit S. uberis. Was ist da die Empfehlung?

Exp. AB-TS und versiegeln, weil er ein Umweltkeim ist. Aber ob er immer wieder kommt oder es eine Neuinfektion ist, das kann ich nicht sagen. Wenn S. uberis vermehrt auftritt ist das Gelichgewicht gestört. Wo kann man da ansetzen? Das Problem liegt bei der Immunität der Kuh.

VET: Abrupt TS mit …?

Exp: Ja, aber bei einer Kuh mit einer Leistung von 30 Kg natürlich schwer.

VET: Bei manchen funktioniert das gut.

VET: Orbeseal funktioniert gut.

Exp: Man muss auch bedenken, beim TS ist es ja so, dass keine Milch im Euter ist, also weniger Nahrung für die Keime. Und beim TS geht zwar am Anfang das Immunsystem runter, dann jedoch wieder hoch.

VET: Man kann ja impfen! Die Paraimmunität erhöhen.

VET: S. uberis ist ja in der Umwelt und 70-75% der Gustviertel haben einen schlechten Verschluss. Daher empfehle ich bei S. uberis vermehrtes Zitzentauchen. Orbeseal könnte das aber auch verhindern, oder?

Exp: Ja

VET: Bauern, die mit hoher Leistung Trockenstellen aber Orbeseal benutzen. Meiner Meinung nach hilft das da nicht, da die Milch das Orbeseal ins Euter "schwemmt" bzw. die Milch am Pfropf vorbei leckt.

Exp: Ich habe die Erfahrung gemacht, dass bei Orbeseal oft viel Luft noch in dem Injektor ist. Die sollte man vorher raus lassen. Darauf muss man Tierhalter hinweisen.

VET: Ich empfehle den Bauern, dass sie das Orbeseal "runtermassieren", in die Zitze

Exp: Abschliessend kann man sagen, wenn die Eutergesundheit stimmt, sollte man nichts ändern.

Nebenthema Trockenstellen: "CNS"

a. **Erfahrungsaustausch**
    i. Wer macht Spezifikation?
    ii. Empfindet Ihr diese als Hilfreich oder als Verwirrend?
    iii. Was macht ihr dann damit? Was ist die Konsequenz?

VET: Wir machen den Maldi-Tof von XYZ. Ich empfinde es 50/50 als hilfreich bzw. verwirrend. Am Anfang war es mehr verwirrend, jetzt ist es eher interessant. Ändern tun wir jedoch nicht so viel. Diejenigen, die beim Schalmtest angeschlagen haben, werden eh behandelt.

VET: Behandelst du mit Pen G oder machst du ein Antibiogramm?

Vet: Bei XYZ gibt es eine Tabelle mit den gängigen Resistenzen. Da schaue ich immer drauf.

Exp: Allgemein kann man sagen, dass 40% Penicillin resistent sind. Z.T. sind sie auch Penicillin und Cloxacillin Resistent, jedoch Amoxicillin sensibel. Das wird aber nicht vom mecA-Gen versursacht.

VET: Dann macht es aber doch keinen Sinn mit Gentapen zu behandeln?

Exp: Erfahrungsgemäss funktioniert das ganz gut, allerdings gibt es da die unwirksame Komponente Penicillin drin, was nicht ganz unproblematisch für die weitere selektion von reistsnten Populationen sein kann. Trockenstellen am besten mit Biclox.

VET: Der Plan für dieses Jahr ist mehr Antibiogramme zu machen und dann zu schauen.

VET: Und VET mit Schalmtest positiv meinst du, dass die im Schalmtest positiv waren und mit einer hohen ZZ aufgefallen sin, oder?

VET: Ja

VET: Der Vorteil vom einschicken ist ja auch, dass man Zeit gewinnt, und es evtl. ein paar Tage später schon wieder gut ist.

Alle: Zustimmung

VET: Wir differenzieren nicht. Haben ein paar Mal Resistenztest gemacht Hat das überhaupt Behandlungskonsequenzen? Das Geld kann man sich doch wahrscheinlich sparen.

VET: Ich probiere oft die nicht zu behandeln.

Exp: Seht ihr klinische Viertel?

Alle: Sehr selten

VET: Ab und an sehe ich schon eins. Die gibt es schon.

VET: Aber kann es da nicht sein, dass es eigentlich ein Coli Viertel ist, und Coli eben schon kaputt ist?

VET: Klar, das ist möglich

VET: Wir differenzieren auch bei XYZ. Und es hilft nicht wahnsinnig, verwirrt aber auch nicht. Wir beobachten. Und schauen bei den Betrieben, ob sich was ändert.

VET: Wir differenzieren auch bei XYZ

VET: Wir auch. Konsequenz hat das eigentlich nicht. Evtl behandeln wir am Anfang der Laktation mit Schalmtest +++ oder wir schicken am Ende der Laktation die Kuh früher zum TS.

VET: Ich differenziere nur wenn ein Betrieb Probleme hat und ich immer den gleichen Keim auf der Platte sehe. Das wird dann notiert und weiter beobachtet.

b. Expertenrunde

Exp Zusammenfassung der Keime

Staphylococcus chromogenes Ansteckungspotential, aber keine schlimme Reaktion

Staphylococcus haemolyticus Zitzenspitze und Haut, auch überall in der Umwelt

Staphylococcus xylosus kommt überall vor, oft resistent, besiedelt Euter und Strichkanal

Staphylococcus vitulinus besiedelt Strichkanal

Staphylococcus sciuri MecA-Gen rel. häufig vorhanden, also resistent, kommt eher selten vor

Staphylococcus equorum Macht in Deutschland mehr Probleme (glaub ich), in der Schweiz eher nicht, ist auch eher im Strichkanal zu finden

Exp: Alle sagen die Endkonsequenz von der Differenzierung ist gemach, gemach, mal abwarten

Bei mir ist es anders, da ich ja auch ein Akademisches Interesse an der Differenzierung habe

Man kann sehen, dass die Keime Betriebsspezifisch sind. Bei einer konstanten Herde bleibt das Resistenzmuster gleich. Ein Antibiogramm reicht vielleicht alle halbe Jahre um zu sehen, ob sich was geändert hat.

VET: Das Resistenzmuster bleibt gleich?

Exp: Ja, nur wenn aggressiv Makrolide eingesetzt werden verändert es sich rel. schnell. Sonst bleibt es konstant. Evtl. tauschen die verschiedenen Spezies die Resistenz Gene untereinander aus, das ist aber noch nicht ganz klar.

VET: Wie teuer ist das Differenzieren denn?

VET: 22 CHF mit der Differenzierung bei XYZ

VET: Und der Resistenztest ist separat?

VET: Ja

VET: Warum können die das bei der PCR nicht unterscheiden?

Exp: Die sind sich wahrscheinlich zu ähnlich

VET: IDEXX hat den Maldi-Tof und XYZ die abgespeckte Version

VET: Wir kreuzen Maldi-Tof nur an, wenn es unsere Landwirte auch interessiert.

Exp: Das ist auch eine finanzielle Frage. Aber Diagnostik für Herdenprobleme wird evtl. günstiger in Zukunft, der Bund will gezielt die Diagnostik im Fall von Bestandesproblemen unterstützen

VET: Am Anfang haben wir nicht differenziert, das hat sich geändert.

VET: Ja, mehr Landwirte wollen einschicken.

Exp: Das Ganze diskutieren hat schon einen Effekt

VET: Mit den Tuben ist es ja das gleiche, zuerst wollen sie nur die eine, und dann diskutiert man mit Ihnen, dass es andere Tuben erst nach der Untersuchung gibt.

Exp: In Kanada versuchen die nun selektives TS pro Viertel.

VET: Da bringt ja nix

Alle: Zustimmung

Exp: Habe ihr schon mal Staph epidermidisgehabt?

Alle: Nein

Exp: Der ist multiresistent, bildet einen Biofilm, hebt gut im Euter, ist ansteckend und macht hohe ZZ

VET: Ich habe neulich die Behauptung von jemandem gehört, dass ja alle Keime einen Biofilm machen

Exp: Alle? Das wäre dann ja blöd.Das Gen für Biofilmbildung gilt als wichtiges Virulenzgen und färdert die Persistenz im Euter, gleichzeitig schwer angreifbar für AB. Gilt für gewebe und für die Milchleitungen.

VET: Habt ihr schon von dem Enzym Präparat gehört? Heisst glaub ich Veyxin? Soll wohl zuerst den Biofilm auflösen, Ein Betrieb mit einem S. uberis Problem hatte damit super Erfolge.

Exp: Biofilmentfernung funktioniert nach meiner Information nur mechanisch. Aber wer weiss?

VET: Oder pflanzlich.

VET: Ja, stimmt. Zuerst Veyxin und nach 12 h dann Pen.

VET: War S. uberis chronisch oder akut?

VET: Nach Milchprobe haben wir S. uberis festgestellt.

VET: Tuben mit Vit. A sollen auch helfen.

Exp: Der Biofilm ist eher Thema bei Chronizität.

VET: Bei den Kleintieren nimmt man bei den chronischen Blasenentzündungen ja auch Cranberry. Das bricht wohl den Biofilm auf.

VET: Das Enzympräparat heisst Masti Veyxym (im Internet nachgeschaut)

Exp: VET, was war denn die Konsequenz von dem Herrn, der das gesagt hat?

VET: Das war nur ein Nebensatz von ihm. Ich habe dann gestutzt ob das so ein Problem ist

Exp: Man kennt ja die Gene zum Bilden eines Biofilms, aber man weiss halt nicht, was sie damit im Euter machen bzw. wann sie aktiviert sind und wann nicht

VET: Man kann auch sagen, Kuh überlebt trotz unserer Therapie.

VET: Oder, weil wir nix machen.

Alle: Zustimmung

VET: Ich habe nochmal eine Frage zum selektiven TS. Wie ist das aus wissenschaftlicher Sicht?

Exp: Man kann Keime auch mit Behandlung zum TR auf Resistenzen selektionieren! Aber wenn man während der Laktation eine Mastitis behandelt, geht AB in Milch, die wiederum in die Umwelt kommt. Das bleibt ewig im Boden und hat höchstwahrscheinlich einen grösseren Einfluss aufs grosse Ganze.

VET: Und was tut man den Kälbern an mit AB-TS, da bleiben doch bestimmt Restmengen im Kolostrum?

Exp: Die AB sind dann schon unter der Nachweisgrenze, aber wahrscheinlich noch vorhanden.

VET: Mikromengen werden bestimmt auch über den Kot ausgeschieden.

Exp: Ja

Exp: Und bei AB Gabe währende der Laktation werden eben in weniger Zeit mehr AB ausgeschieden. Minus malum! Es ist extrem schwer da das "richtige" zu finden. Auch ethisch. Aber wenn auf einmal doppelt so viele AB in der Laktation eingesetzt werden müssen, das kann es nicht sein. Man muss auch nicht 100% ohne selektiv TS, aber mit 85 oder 95% ist auch gut. Man braucht halt einen Plan. Und man kann sehen, dass die Diskussionen gefruchtet haben

Feedbackrunde

VET: Bezüglich CNS Schwer. Ich werde das "erstmal-nicht-behandeln" beibehalten. Und man muss nicht alle einschicken, ausser wenn es massiv ist. TS Das hat sich evtl. etwas drastischer angehört, als es eigentlich ist. Wir haben halt nie die Diskussion gesucht. Aber manche Landwirte haben es bestimmt irgendwie umgesetzt, denn auch bei uns ist der Verkauf von Orbeseal hoch.

VET: CNS Auch ich werde nicht alle gleich behandeln, und mit dem Antibiogramm ein wenig zurückfahren. TS Wichtig ist für mich weiterhin, dass der Tierarzt entschiedet und wir werden weiter motivieren, bei denen wo es gut funktioniert. Ich will auch mal schauen wie viel sie bezogen haben

Exp: Einwurf vom AK Ost Schweiz Die haben gesagt, dass sie die TAMV Besuche gerne zum Anlass nehmen um das zu besprechen. Und die Landwirte nehmen es gerne an.

VET: Ja, das macht Sinn dann mit Landwirten zu reden.

VET: Beim TS. Wir haben viel erreicht, aber was macht man mit den schwarzen Schafen? Ich hatte gehofft, dass die bei der blauen Kontrolle auffliegen, aber das war leider nicht so. Ich werde meine Strategie beibehalten

CNS Bei Betrieben mit viel CNS werde ich in Zukunft differenzieren.

VET: Bei CNS werde ich mit den Antibiogrammen zurückfahren

TS Da fahre ich so weiter. Suche weiter die Diskussion. Bei Betrieben mit S. uberis Problemwerde ich evtl. wieder Antibiotisch TS um den S. uberis Druck zu senken.

VET: TS Euterschutz ist nicht schlecht. Und wir sind nicht auf so einem schlechten Weg. CNS Abwarten ist nicht schlecht. Weiterhin beobachten.

VET: TS Ich bin froh, dass ich nicht verurteilt worden bin. Ich schaff aber dran. Bei Betrieben mit einer guten Eutergesundheit, da könnte es funktionieren. CNS Probiere es so weiter. Abwarten Die Info von XYZ war eine gute Info. Und was ich auch gelernt hab ist das mit dem MecA und B

Exp: MecA ist das Gen, das für eine Multiresistenz gegen alle Betalactame codiert. Im ZOBA machen sie immer ein normales Antibiotgramm (MHK) und wenn eine Oxacullinresistenz auftritt untersuchen sie noch auf das MecA Gen. Oxacillinresistenz ist also ein Indikator für eine Methicillinresistenz also eine Multiresistenz.

VET: Ich bin nicht neben der Spur. Auch andere haben die Erfahrung gemacht, dass mehr Mastitiden auftreten beim Trockenstellen ohne AB. Ich werde evtl. mal einzelne Betriebe auswerten.

CNS Sehr viele im AK machen schon Resistenztests bei Keimen. Das kann ich intensivieren. Aber ich hatte früher auch mehr Problembetriebe. Jetzt deutlich weniger.

CNS Vielleicht mal intensiver nachschauen, wir behandeln nur bei Klinik.

VET: TS Ich steh nicht komplett alleine da und AB-TS ist nicht nur schlecht. Ein wenig pragmatischer anschauen. Und Reden! Wir sind auf keinem schlechten Weg.

Exp: Ihr fühlt euch alle sicherer als früher. Das ist eine super Entwicklung.

Mod: Vielen Dank für die tollen Treffen. Ihr wart eine sehr angenehme Truppe, habt euch ausreden lassen, auch ernst genommen, mich als Moderator ernst genommen. Alles in allem Sehr angenehm!

Nächster Arbeitskreis bzw. Abschlusstreffen

Viele Teilnehmer sind interessiert an einer Fortführung des Arbeitskreises.

VET hat schon einige Themen im Kopf Kälberaufzucht, Fütterung,…..

Die Interessierten sollen sich bitte bei Exp melden.

Protokoll Melktechnik

VET: in den letzten Jahren zweigeteilt: ein Teil der Bauern lasst es sein und macht nichts, anderen klären mit Hersteller ab (insb. die mit Melkroboter) oder mit ZMP oder anderen Melkberatern. Gute Erfahrungen mit Roboterbetriebe gemacht.

VET: ab und zu Melkberater dazu gezogen, hängt von ZZ, AB-Verbrauch und Bakteriologie ab. Zum Beispiel C.bovis und keine akute Mastitis, eher Richtung Technik gesucht, also Maschine oder Melkarbeit. Manchmal sieht sie selber gewissen Sachen, aber manchmal ist es besser wenn man sich eine zweite Meinung holt.

VET: gewisse Betriebe verbrauchen viel Oxytocin

VET: Guischeint sensibler als anderen zu sein (Milchablassen)

Vet: möglich, Genetik spielt auch eine Rolle, HF sind so gezüchtet, dass sie die Milch gut geben

VET:er/sie fragt nach den Grundsätzen, wie Melken, Handschuhe ja/nein, Maschinenservice, Dippen, Desinfektion. Viele Antworte so gefunden, gerade bei grossen Problemen wie S.aureus.

VET: seine erste Frage ist „wann war den letzen Service?“. Weiter beobachtet er das Melken und den Anzahl Tiere, die vorhanden sind und Probleme haben. Behandlungen werden grundsätzlich am Fressgitter gemacht und die Milchproben selber genommen. Probleme mit Keime wie C.bovis werden schneller abgeklärt. Bamos stellt auch einen Melkberater zur Verfügung. Hat gerade einen Fall gehabt von einem Neubau mit Kriechstrom.

VET: beobachtet wie VET, dass die ZZ im Sommer steigen, im Zusammenhang mit Mais (Mykotoxinbelastung Grünmais)

Vet: nimmt zuerst Milchproben (selber ausgewertet), schaut wie Bauer melkt, auch Basics (Vormelken, nassen Zitzen, Verhalten der Tiere). Wenn das Gefühl da, dass Technik schlecht ist, lässter/sieschnell den Berater kommen. Es ist zudem auch gut, dass jemanden die gleichen Punkte betont.

Berichtet von einen Anlass mit Bauern, wo ihr klar geworden ist, dass nicht alle Bauern den Basics kennen (zB Kolostrumversorgung), obwohl es für TA klar ist.

VET: legt Wert auf Melkhygiene und Anrüsten, es ist hilfreich, den Bauern ein Bild zu zeigen (zB Effekt von zu frühes Ansetzen auf Milchflusskurve).

VET: fragt EXP wie das Aggregat trocknen muss – EXP: nach Waschen von Fixierung wegnehmen, damit Luft reingehen kann und Trocknen ermöglicht wird, ansonsten Gefahr einer Biofilmbildung. Das Melkgeschirr muss schön hängen und nicht abgeknickt sein, sonst schlechte Trocknung und Rissbildung im Gummi.

Vet: wie gross ist das Problem mit resistenten Dippmitteln?

VET: laut Deutschen Experte, ist Kresol nicht brauchbar. Früher gab es keine Resistenzen, jetzt immer mehr.

Exp: viele Dippmitteln auf dem Markt, Swissmedic testet auf Wirksamkeit, aber evt sind anderen auch gut

Exp: beste Erfahrung gemacht mit Jod, mit Chlorhexidin hat er das Gefühl, dass es mehr Resistenzen gibt (zB Pseudomonas). Jod kann theoretisch trocknen und reizen, aber wenn pflegende Komponenten drin sind, ist es gut. Milchsäure gibt schöne Zitzen, aber zu schwach desinfizierend, insbesondere für S. aureus-Betriebe. Was gut funktioniert, ist die Kombination von Milchsäure und Peressigsäure. Wichtig ist, dass der Becher immer sauber und getrocknet wird. Wenn zu viel Desinfektionsmitteln angewendet werden, kann es zur Hautablösung kommen, vor allem wenn Chlorhexidin mit Kalk (Kalkmatraze) kombiniert. Man soll Kohlensäurekalk brauchen.

VET: ist Peressigsäure krebserregend? EXP: unklar, aber die Lüftung ist sicher ein Faktor. Peressigsäure alleine in Roboteranlage zu kurze Wirkung, Dampf als Zwischendesinfektion ist besser.

Exp: berichtet von Klebsiellen an Roboterbürsten.

Exp: sagt, dass im Normalbetrieb Peressigsäure genügend ist. Hygiene ist aber die Grundvoraussetzung für eine gute Desinfektionsmittelwirkung.

Es gibt vielen Pflegemitteln, aber man soll aufpassen, da Keimverschleppung möglich.

Peressigsäure funktioniert als Zwischendesinfektion gut, zwischen Gruppen soll es in Melkbecher gesprayt werden. Problem in Anbindestall, da es nicht lang genug wirksam ist. Melkbecher zu sprayen wirkt besser als tauchen, und Peressigsäure funktioniert gut trotz Milchreste, ist auch kein Problem für Biobetrieb und gibt kein Problem mit Rückstände in der Käserei. Chlor greift hingegen die Haut an und Rückstände können problematisch sein.

VET: tauchen nicht besser? Spülen?

Exp: spülen nicht nötig, sprayen in Becher genügt, 30 Sekunden wirken lassen (1 Minute ist sicherer), Konzentration 1000 ppm ist gut. Nachteil: Gummi ist dann nicht trocken, aber ist eigentlich generell nicht wirklich trocken.

VET: gibt es eine chemische Reaktion von Chlor mit Kalk? EXP: ja, Bluemax (blauen zitzen -massive hautreaktionen).

Exp: einen Dippmittel mit Zuckerrübenkalk, hat viele Probleme gemacht - Bakterienkontamination von Zuckerrüben. Zitzentauchmittel wurden früher von Swissmedic auf Wirkung gepüft, jetzt wird nur geschaut, dass keine verbotene Stoffe drin sind.

Exp: zur Frage Zitzen tauchen oder sprayen? Hängt vom Präparat ab (Viskosität). Beim Spray ist das Problem das Zielen und braucht mehr Mittel um rundum zu treffen. Becher ist ok wenn man zwischen das Melken wäscht. Non-return ist auch ok, man muss es aber nach 2 Melkzeiten auswechseln.

Vordippmittel gute Sache und immer mehr gebraucht, aber man muss aufpassen da es wenig desinfizierende Wirkung hat und mehr Bakterienkontamination möglich, da auch an dreckige Zitze angewendet (nach dem Melken muss man den Behälter waschen).

EXP: Schaum vor dem Melken gut. Man muss der Schaum einwirken lassen, gruppenweise anbringen/putzen, abnehmen mit Reinigungstuch (trocken). Löst Dreck gut und trocknet Zitze nicht aus aber bleibt auch nicht nass.

Zudem: Vormelken reicht als Stimulation, zT bei gewissen Tiere reicht Predippen schon. Vormelken ist zentral zur Melkbereitschaft.

Mod: schauen TA auf das (Vormelken/genügende Stimulation)?

Vet: ja, 1 min, wie lange, alle Tiere gleich?

VET: nur wenn nicht leer werden, ansonsten nicht speziell.

Exp: es gab Fälle, wo sie massive Melk- und Zellzahlprobleme hatten, wo das Vormelken/Anrüsten der Schlüsselpunkt war.

VET: Oxytocin ist das Wohlfühlhormon - es braucht die Zusammenarbeit von der Kuh, dem Melker und der Maschine.

Exp: Anrüstautomat - zT auch Fälle wo bei 10% der Tiere nach Umschaltung nichts gekommen ist.

EXP: wenn die Kuh nicht vorbereitet und angehängt wird, dann ist die Zitze schlaff und hat kein Kontakt zur Wand, danach klettert der Gummi und die Zitze wird oben abgeklemmt. Man kann an den Zitzen gut beobachten, ob der Anrüstautomat gut eingestellt ist, wenn nicht, sind die Zitzen abgedrückt und eine sehr lange Melkdauer zusammen mit Zitzenschäden (Ring). Nicht bei allen Zitzen kann Klettern beobachtet werden (kurze Zitzen), aber es gibt trotzdem eine zweigipfelige Melkkurve. Das Problem tritt auch je nach Konformation auf. Die schlechte Vorbereitung ist aber eine der häufigsten Ursachen. Das Ziehen am Aggregat bzw. belasten des ASammelsückes ist nicht hilfreich. Je nach Laktationsstadium ist die benötigte Vorbereitungszeit unterschiedlich lang, 1 Min idR ok, schneller bei Frischlaktierenden, länger bei Richtung trockenstellen (bis 2 Min). Da wo Tiere in eine rel. kleine

Gruppe im Melkstand gehen ist kein Anrüstautomat nötig, im Gegenteil, mit der Vorbereitung ist der Milcheinschuss gut genug. Ein Anrüstautomat hat hingegen Sinn in einem grossen, langen Melkstand mit vielen Tieren und langen Wegen. Die Frage ist auch, wie der Bauer arbeitet und wie lange er für Vorbereitung hat. Lieber zu lange als zu kurz, sonst klettern die Gummis (s.oben). Die Grenze ist etwa 90 Sekunden, aber bei 2 Min kann es noch gut sein. Wenn zu kurz fast immer problematisch. Frischlaktierende Kühe zeigen zT auch Schmerzen, da Druck weh tut. Wenn keinen Einschuss vorhanden ist, dann geht es erst nach 5 Min wieder (rel. kurze Refraktärzeit).

VET: ist dann weniger Überwachung in Betriebe mit Anrüstautomatik gefragt?

EXP: zum Teil, aber in grossen Gruppen geht man auch schnell von Kuh zur Kuh. Vormelken muss in beiden Fälle (mit oder ohne Anrüstautomat) gemacht werden.

Mod: wie ist es im Anbindestall?

EXP: Automat gut, muss aber gut eingestellt werden. Es gibt Flussgesteuerte vs Zeitgesterute vs kombinierte Systeme. Die hochfrequente Vibration bringt keinen Vorteil, zieht Luft und die Kühe sind dann unwohl (GEA). Delaval ist weniger hochfrequent. Exp: zT erschrecken die Kühe.

Flussgesteuert: müsste mindestens 6-8dl (Duovac) sein, weniger (wie 2dl) ist nicht sinnvoll. Es gibt alte Modelle, die bei gleichem Fluss an- und abhängen, ist nicht ideal. Es gibt auch individuelle kuhgebundene Modelle. Roboter: früher ging es zu lange um anzusetzen, heute ist es fast zu knapp. Man sieht beim Ansetzen ob der Milcheinschuss da ist, man kann es einstellen (z.B. länger Bürsten bzw Waschen je nach Modell).

Mod: Frage an VET - Erfahrung mit Einstellungsproblemen beim Roboter?

VET und VET: ging zT lange, bis das Problem gelöst wurde.

Exp: bei Lely ist manchmal die Vorbereitung problematisch, ergibt nicht eine schöne Kurve, zT zweigipflig.

Exp: Die Abnahmeautomatik ist auch ein Thema, zT zu früh, zT zuspät

EXP: wenn die Tiere nicht komplett ausgemolken sind, gibt es den Eindruck, dass das Aggregat zu früh weggenommen wird, aber das Problem ist vorher. Der Schwellenwert ist auch einen Faktor. IdR werde 2dl eingestellt, was nicht schlecht ist, aber bei Kühe die viel Milch geben sind 4 dl auch ok. Nach Abnahme muss man warten bis zur Kontrolle (30 Sek, damit sich der Druck oben löst), man sollte noch Milch melken können (zB für CMT). Das Ablassen muss sanft geschehen, zuerst Lufteinlass während 5 Sek., dann wegnehmen. Man muss nicht ziehen, sonst gibt es Rückspray (und Vakuumschwankungen) - dh Erregerverschleppung, insbesondere an nassen Zitzen. Der Lufteinlass muss funktionieren (hörbar). Wenn noch viel Milch nach dem Melken vorhanden ist, ist es ein Zeichen für Klettern. Klettern wird durch nassen Zitzen und eine schlechte Vorbereitung begünstigt.

Schnellen, hohen Vakuumschwankungen (durch Lufteinbruch) sind schädlich, weniger schädlich wenn oben (Zitzenbasis) 2-3 kPa-Schwankung.

EXP: mit Beobachtung ist viel erreichbar: wie wird vorbereitet, angehängt (wenig Lufteinbruch), wie ist die Position des Geschirrs, kein Zug am Schlauch (hängt runter und zieht, zB bei „Side by side“ und Tandem, es kann ein grosser Einfluss haben und führt zu längerer Melkdauer, grösseren Zitzenbelastung, schlechtes Ausmelkgrad). Ausmelken ist nicht nötig, die Kuh sollte mit schönem

Euter und Zitzen leer werden. Bei alten Kühe mit schlechte Konformation ist es anders, aber Zug ist nicht notig.

Exp: nicht alle Melker haben die gute Routine und es ist schwierig, dies umzustellen. Falls Messungen gemacht werden, ist es sinnvoll nicht zu direkt zu viel zu kritisieren und Fragen zu stellen (insbesondere über das, was gemacht werden soll). Am besten beobachten, wie gearbeitet wird.

EXP: nach dem Melken müssen die Zitzen angeschaut werden: Ring, Farbe (blau, weiss). Falls es oben einen blauen Ring gibt, ist es dass es zu viel Vakuum oben gibt, zb wenn ein grosser Gummi klettert und die Zitze einschnürt, oder auch wenn der Gummi zu eng ist.

Vet: gewissen Bauern sind betriebsblind und merken zB die Ringe nicht.

Exp: melken ist etwas sehr persönliches, Diplomatie ist nötig.

EXP: darum ist es mit Messung einfacher, da man ein Problem auf eine Kurve zeigen kann (zB: bei 4er Swing-over-System zT sehr langen Zeiten bis zum Melken der Kuh)

Mod: wann wird um Hilfe gefragt?

Z.B. beim ZMP wird wenn man Richtung Abzüge geht, eine Beratung empfohlen. Die Schmerzgrenze ist unterschiedlich, bei gewissen Bauern 800000 Z/ml, anderen 300000 Z/ml.

VET: es gibt auch solchen, die die eigene Performance verbessern wollen und darum ein Techniker holen.

Exp: häufig ruft der TA an, wenn die Sperre droht. Häufig heisst es „der Bauer will was machen“ - aber eigentlich eher der TA. Ein Kompromiss zu Beginn hilft, es ist einfach die Anlage anschauen zu lassen als sich direkt beobachtet zu fühlen. Die Anlage kann als Einstieg dienen.

EXP: die Anlage gehört sowieso dazu. Beurteilt wird die Installation: Vibration, wie lange die Leitung ist, wie die Milch fliesst, gibt es unnöntige Bogen. Aber Vorsicht: eine schlecht installierte Anlage kann trotzdem gut funktionieren, eine Nassmessung ist nötig! Zeichen für Probleme in der Melkanlage oder Melkprozess: Unruhe, häufige Lufteinbrüche, schlechter Ausmelkgrad, Zitzenveränderungen, chronisch erhöhten oder schwankende ZZ.

Die Servicemessungen sind Trockenmessungen, d.h. Anlage ist nicht am Tier in Betrieb - aber es wird an der Zitze gemolken, d.h. es ist wichtig, was an der Kuh passiert.

Vet: wenn der Service gut ist, heisst es nicht dass Anlage gut eingestellt ist?

EXP: Man muss die Vakuumhöhe, die Ventile, der Verschmutzungsgrad, der Lufteinlass, der Gummiwechsel, und die Reinigung beurteilen. Eine Stromabklärung gehört auch dazu - die Messung ist relativ einfach mit den richtigen Geräten, das Beheben ist schwieriger. Elektriker haben früher die Erdung gemessen, nicht der Strom (dafür braucht es eine Stromzange). Der Bahnstrom kann Strom im Elektrosystem im Stall beeinflussen. Das Problem ist meistens lösbar: Erdung, Frequenzgesteuerte Pumpe und Ventil, Potentialausgleich). Es soll kein Strom zwischen verschiedenen elektronischen Systemen im Stall zirkulieren. SBB-nahe Betriebe haben mehr Probleme. Tiere sind empfindlicher als Menschen, die Resistenz unterschiedlich (3-500 Ohm Kuh, Mensch viel mehr).

Mod: Konkrete Fragen zu den Gummis?

EXP: Werden idR zu spät gewechselt. Es gibt den Silikon und schwarzen Gummis, das Melken ist etwa gleich. Silikongummis überleben ca 5000 Melkungen, Schwarzgummis ca 2500 (halbjahrlicher Wechsel, ausser in sehr grossem Melkstand). Silikon ist doppelt so teuer, somit kein grossen Unterschied. Gummis werden mit der Zeit locker (verlieren Spannkraft, es braucht Spannung um zu

massieren, werden oval). Schwarzen: Gummi mit Aufweicher gemischt, zT Unterschiede zwischen Chargen (seltener bei Silikon). Kopfhöhe der Gummis: hängt auch von Zitzenlänge ab. Kurze Zitze: flacher Kopf, massieren weiter oben und sind enger, nicht gut für grosse Zitzen. Ein runder Kopf passt sich besser am Euterboden an, aber ziehen mehr Luft an als flachen. Durchmesser: 24mm gut für gröbere Zitzen. Der Berater hat idR Erfahrung um richtig zu wählen. Besser zu grosser als zu kleiner Gummi. Problem Gummi oder Melken: viel Ringe, blaue oder weisse Zitzen, nassen Zitzen. Gummi zu eng: langsam gemolken und Zitzen werden verengt. Zu weit: eventuell Ringbildung und Blauverfärbung oben an der Zitze sichtbar wegen Klettern.

VET: muss man verschiedene Grössen von Gummis brauchen? Exp: Frischlaktierende evt separat melken, dann umstellen, kann eine Lösung sein. Es gibt Gummis mit verschiedenen Durchmesser und Formen (rund, dreieckig, früher sternförmig).

VET: Bedeutung der Hyperkeratose?

EXP: Überlastung, hohes Vakuum, oder zu langes Melken bei tiefem Vakuum, Blindmelken am Zitzenende, schlechte Vorbereitung und langes Melken.

Taktverhältnis (Saugphase:Entlastungsphase): 60:40 Druckschaden häufiger im Vergleich mit zu wenig Massage. 65:35 melkt schneller.

Elektropulsatoren sind heute konstant und genau, aber sie sind relativ aggressiv im Gegensatz zum Hydropulsator. Jetzt wird ein Puffer eingebaut damit der Zitzengummi etwas sanfter schliesst. Es bringt was, man hört es und sieht es bei der Kuh, auch messbar.

VET: manchmal gibt es Kühe, die wie einen Schleimhautprolaps des Strichkanals haben wenn sie aus den Robotern kommen und mit der Zeit verhornt es.

EXP: Wenn Einzelfall, kann es ein Problem der Kuh sein (spitzige, feine Zitzen), wenn bei vielen Tiere dann ist es eher ein technisches Problem.

Exp: Wenn eine Kuh viele Zitzen mit Hyperkeratose Score 4 hat, dann kriegt es man fast nicht mehr weg. Bei den wenigsten, geht es über die Gustzeit weg. Die Hyperkeratose ist auch problematisch um eine saubere Milchprobe zu nehmen.

Nasse Zitzen: Problem beim Vorbereiten, oder wenn am Ende: Milchstau (zu kleines Sammelstück, früher kleinen Durchmesser des Milchschlauches, häufig bei Rückspray).

Exp: Euterwaschen ist ein Problem wenn nicht richtig abgetrocknet wird. Es ist besser, eine saubere Boxe zu haben, als Zeit ins Euterputzen zu investieren. Da sammelt sich dreckiges Wasser an der Zitzenspitze.

VET: Muss man Kühe, die viel Oxytocin verbrauchen, ausmerzen?

EXP: man muss schauen wo das Problem ist (Melktechnick).

Exp: es ist schwierig, die Tiere zu entwöhnen. Der Stress (zB Plazverhältnis in Side by side) hat einen Einfluss, aber es ist nicht das Adrenalin sondern wahrscheinlich andere Komponenten (Aussage Bruckmaier

EXP: Milchflussgeschwindigkeitmessgeräte QID für TA sind nicht sehr teuer, aber nicht sehr spezifisch, man muss auch wissen welche Kuh wie vorbereitet wird.

Ideal wäre, mehrere Messungen zu machen, aber wenn man alles einmalig anschaut, sieht man viel. Mehrere Druckmessungen (mehrere Tiere) sind sehr wertvoll.

Exp: hat sich mal überlegt selber auch zu messen, aber es gibt gute Leute dafür, und es braucht Zeit um es richtig zu lernen und zu machen. Es gibt nicht viele Leute, die es machen, aber darum sind die die es machen gut.

Mod (und alle): Danke EXP!

Feedbackrunde

VET: die Erklärungen über die Melktechnik und Nassmessungen (Exp) waren eine gute Auffrischung und zT auch neues gelernt. Es ihm deutlich geworden, dass man sich in ein Thema einarbeiten muss, um die verschiedenden Fälle auseinander zu nehmen.

Vet: idem (Erklärungen EXP), das Interesse wäre da aber keine Kapazität, um sich in diesem Thema zu vertiefen. Es war ihr nicht bewusst, dass die Kuh so sehr auf Strom reagiert.

VET: mitgenommen, dass Peressigsäure (PES, zur Desinfektion) auch in Kombination mit Milchsäure und Pflegemitteln funktioniert. Elektronischen Pulsatoren viel „schärfer“ als alten „analogen“.

VET: Erläuterungen von EXP sehr spannend, hat wenig Ahnung von Technik. Nimmt auch mit, dass man verhindern soll, eine Beratung zwischen Stall und Auto (so schnell nebenbei) machen soll.

VET: EXP hat guten Bilder vermittelt. Wichtigkeit des Anrüstens bewusst geworden. Auch die Tatsache, dass man Desinfektionsmitteln mit Pflegemitteln kombiniert, ist interessant.

VET: s. VET (Kombination PES/Milchsäure). Gelernt, dass auch wenn man das Gefühl hat, das „hinten“ etwas nicht stimmt (Kühe werden nicht leer gemolken), dass man alles anschauen muss (auch Arbeitsbeginn, insb. Vorbereitung). Beobachten ist sehr wichtig und bringt sehr viel, auch bei „normalen Bauern“ die es gut machen. Die Melkarbeit anzusprechen ist heikel, zuerst bei der Maschine/Service anzufangen ist ein guter Tipp.

VET: s. VET. Möchte sich mehr mit Melktechnik und Messungen beschäftigen, aber braucht viel Zeit und wird wahrscheinlich doch nicht den Fall sein.

Exp: ist besser in Beobachten, als mit Geräte zu arbeiten. Man kann ermitteln, in welchem Schritt der Melkarbeit, das Problem liegt (Triage). Wichtig ist auch, zuzugeben wo die eigeine Limit ist und wann Hilfe benötigt wird.

Mod: AK weiterfühern?

VET: ja! Jeder soll sich Themen Überlegen und per Email melden. Es wird kein neuer Termin mit neues Thema jetzt abgemacht, zuerst findet die gemeinsame Veranstaltung statt.

VET: Milchinhaltsstoffe wäre ein Thema

Protokoll Diagnostik

Expertenrunde

Vet: Welcher Agar ist sinnvoll?

Exp: Zuerst Ziele und Genauigkeit der Diagnostik festlegen und danach den Agar aussuchen.

Vet: Antibiotika-Plättchen

Exp: Das Ergebnis der Plättchen (resp. der Hemmhof) ist stark abhängig von verschiedenen Faktoren wie pH-Wert und Feuchtigkeit.

Die Richtlinien von CLSI und EUCAST verlangen Spezialmedien für die Resistenztestung mittels Disk Diffusion, welche sich unterscheiden von den Medien für die allgemeine Bakteriologie. Das zu testende Isolat muss rein sein, sich in der exponentiellen Wachstumsphase befinden (nach rund 18-28h Bebrütung) und in einer bestimmten Dichte auf die Testplatte aufgetragen werden.

Betalactame werden rasch durch Hydrolyse inaktiviert, weshalb insbesondere die Plättchen aus dieser Wirkstoffgruppe immer in Röhrchen mit einem Exsikkator aufbewahrt werden müssen und diesem jeweils nur kurz entnommen werden sollten.

Fazit von allen: Einsatz von AB-Plättchen gestaltet sich im Praxislabor schwierig

Vet: XYZ Test

Vet hatte Exp schon einmal per Mail dazu befragt. Das Fazit war, dass dieser nicht Praxistauglich ist.

Vet: Selektivplatte Vétoquniol

Vet: Eignet sich, um den Äskulin Agar zu benutzen.

Vet: Äskulin Agar: Wie zuverlässig um S. uberis zu diagnostizieren?

Exp: S. uberis lässt sich nicht zuverlässig mit Äskulin Agar allein oder einem Schnelltest diagnostizieren, Goldstandard: MALDI TOF. In der Praxis könnte jedoch der Äskulin- mit dem PYR-Test (s. unten) kombiniert werden, um die ebenfalls Äskulin-positiven Enterokokken auszuschliessen.

Vet und Vet diagnostizieren S. uberis nie direkt.

Vet: filtert mit XYZ Latex Agglutination Test Enterokokken raus.

Fazit von allen: Es gibt eine Fehlerquote, mit der man leben muss.

Exp (nachträglich Anmerkung):

PYR-Test

Wir verwenden den Testkit (Testblättchen und Tropfflasche mit Reagens) von Remel, REF R30854301. Zu beziehen via Oxoid oder Thermo Fisher Scientific.

„Pyroglutamat-Test für Pyrrolidonyl-Arylamidase (Pyrrolidonyl-Arylamidase-Test bzw. PYR-Test) ist ein Verfahren zur schnellen kolorimetrischen Bestimmung bestimmter Gruppen von Bakterien auf der Basis der Aktivität des Enzyms Pyrolidonyl-Arylamidase. L-Pyroglutaminsäure-Beta-

Naphthylamid ist in die Testplatte imprägniert und dient als Substrat für den Nachweis von Pyrolidonyl-Arylamidase. Die Hydrolyse des Substrats führt zur Bildung von Beta-Naphthylamid, welches sich mit dem PYR-Reagenz (p-Dimethylamino-Zimtaldehyd) verbindet, und eine hellrosa bis kirschrote Farbe bildet. Ein positiver PYR-Test erlaubt den Nachweis von Streptokokken der Lancefield-Gruppe A (v. a. Streptococcus pyogenes) und Enterokokken.“

Vet: Mikroskopieren, welche Bedeutung hat das für euch?

Vet, Vet, Vet und Vet: Mikroskopieren regelmässig und messen dem Mikroskop eine grosse Bedeutung in der Diagnostik bei.

Vet: S. aureus Diagnostik

Exp: Bei einer doppelten Hämolyse von einem Keim, der aus dem Euter kommt gehen wir von S. aureus aus. Periodische Kontrollen mittels MALDI-TOF haben im Euter bisher nie einen S. schleiferi / pseudintermedius / lugdunensis als mögliche DD erbracht.

Vet macht noch zusätzlich Koagulase Test zur Identifizierung

Vet: Stehen auch Zusatzinformationen auf Laborberichten (z.B. Schleimbildung)?

Exp: Nein, noch nicht einheitlich. Soll evtl. geändert werden

Vet: PCR

Exp: Man muss wissen, was PCR kann und was nicht, ist spezifisch für diejenigen Spezies, für die sie deklariert ist, alle anderen werden nicht nachgewiesen. Ausserdem muss immer beachtet werden, wie die Probe genommen wurde (Pool / Einzelgemelk / steril / aus Tank).

Ist eine gute Diagnostikmethode bei Bestandes Sanierung bei S. aureus.

Vet: Ist eine Genotypisierung sinnvoll?

Vet: Ändert sich was wenn du das weisst?

Einwand: Ja, die Beratung für den LW beim Melken (Melkreihenfolge)

Exp: Genotyp B ist nicht allein für schwierige Verläufe verantwortlich. Soviel von der bislang nicht publizierten Methode bekannt ist, wird nur der Genotyp nachgewiesen, die Spezies S. aureus an sich jedoch nicht. Aus diesem Grund wird diese Diagnostik ambivalent beurteilt.

Vet: Die Genotypisierung gibt einen wertvollen Hinweis für die Beratung. Problem Bestände können danach eingeteilt werden.

Erfahrungen untereinander austauschen

Vet: bei ihrer 1. Stelle gelernt

- Giesst Milch über Blutplatte
- Blutagar, Äskulinagar

- differenziert Enterokokken mit Äskulinagar (liest nach 24h und 48h ab)
- differenziert Staph. und andere, aber nicht S. aureus

Vet: 2-30 Proben am Tag,

gelernt bei ehemaligem Chef, Weiterbildungen und Selbststudium

- Mit Öse ausstreichen auf Blutplatte
- H_2O_2 und Gramfärbung
- Kein Antibiogramm
- Falls man nicht weiterkommt, fragt man bei Exp nach
- Differenziert Staphylokokken (mit/ohne Hämolyse), Streptokokken (mit/ohne Hämolyse) und ggf. Schleimbildung
- Laborordner mit Anleitung

Vet: 2-5 Proben am Tag, TPA macht Labor, gelernt durch Weiterbildung und Selbststudium

- Blutagar, DNAse
- Referenzbakterien als „Qualitätssicherung" einmal im Jahr
- Laborordner mit Anleitung und Bildern von seltsamen Fällen, die man eingeschickt hat
- Subklinische streicht er einen Tag später aus

Vet: Fachtierarzt Labor

- Streicht mit Tupfer aus
- Schafblut und Gassner Agar
- Diff quick
- Plasmakoagulase
- Nimmt Tabelle aus dem SAT Magazin her, um Resistenzlage einzuschätzen
- Hat Flowchart in Praxis für alle Angestellten

Vet: gelernt bei …

- Mit Öse ausstreichen auf Blutplatte
- AB-plättchen

Sein Anspruch ist nicht die sauberste Diagnostik, sondern Hilfe bei akuten Mastitiden

Alle sind sich einig: Anspruch ist anders an ein Praxislabor.

Praxislabor nicht perfekt, korrekt und vollständig.

Klinik muss immer mit einbezogen werden

Vet spricht an, dass es von der Arbeitssicherheit nicht erlaubt ist, selber Bakterien anzuzüchten.

Feedbackrunde

Stimmungsabfrage: Alle kleben gelbe Punkte aufs Smiley Blatt.

- > Zeit nicht optimal eingehalten
- > Aber Offenheit wird geschätzt
- > Guter Austausch unter Kollegen
- > Guter Exp
- > In-house Diagnostik ist machbar, man muss nur wissen, was man will
- > Einfacher als gedacht halten
- > Simple halten
- > 2 gute Ideen zum „mit nach Hause nehmen“ bekommen
- > Stimulierender Austausch
- > So muss Weiterbildung sein
- > Exp sollte das nächste mal 5-10 min Input geben, ein kleines Referat wäre wünschenswert gewesen.

Protokoll– Bestandesmedizin

Moderator:

- Eutergesundheit in Rahmen von Bestandesbetreuung?

- „Diagnostikbesuch“?

VET: bei Bestandesbetreuung Frage „wie sieht’s mit den ZZ aus?“, fragt nach Blätter

VET: idem

VET: Bauer fragt TA aktiv bei Problemen, seit neuen TAMV neuen Blätter zur Kontrolle der ZZ und TR-Management gemacht

VET: Bauer fragt oder TA weist darauf hin, interessant verschiedene Schmerzgrenzen bei Bauern, TAMV-Kontrollbesuche als Diskussionsgelegenheit genützt. Durch vermehrte Diskussion weniger Euterproblemen. Bauern akzeptieren dadurch mehr, dass nicht immer alles antibiotisch behandelt wird.

VET: Bauern mit weniger Interesse an Eutergesundheit sind auch solche, die Aufhören

VET: junge Bauern sind offener über Thema

VET: S.aureus-Betrieb, 1x/Mo auswerten, wollte nach 1 J weitermachen. Anderer Fall immer gut, dann 2 Bestände zusammengelegt (Betriebsgemeinschaft) dann Eutergesundheit plötzlich sehr schlecht, auch langjährig betreut. Beide machen mit Bestandesbetreuung weiter und schätzen es sehr. Zusätzlich noch einen mit punktueller Betreuung, Kommunikation nicht so einfach, aufgefallen dass er mehr Euterinjektoren braucht. Melkarbeit/technik Problem.

VET: ca 5 Betriebe über längere Zeit betreut, es wird geschätzt – Gesundheit wird überwacht, bei anderen ist es nicht so regelmässig

Exp: nicht alle gleich, einer will gar nicht darüber sprechen, bei den meisten ist Betreuung gewünscht und sie bezahlen dafür

VET: stimmt, gewissen denken schwarz/weiss, „mein Vater hat es auch so gemacht…“

Exp: solange der Bauer es nicht als Problem empfindet, gibt es auch kein Problem. TA und Bauer müssen motiviert sein, ansonsten bringt es nichts.

VET: Schmerzgrenze unterschiedlich

VET **jüngere VS ältere Generation, Motivation von Bauer essentiell, Schmerzgrenze ist unterschiedlich und Ziele müssen Betriebsadaptiert sein**

VET **TAMV-Ko als Gelegenheit, Diskussionen anzufangen**

Moderator: Stolpersteine

VET: falls im Stall etwas verändert werden muss (z.B. Spaltenboden vor Melkstand, Bauer will es nicht verschliessen). Problem wenn Bauern nicht so schlau sind, Kommunikation problematisch

VET: immer wieder nachhaken nötig, das Ziel muss an die Herde und Bauern angepasst werden – was ist für das nächste gewünscht? Realistisches Ziel muss vorhanden sein

VET: es braucht Zeit bis Massnahmen wirken, geht nicht schnell

Exp: hilfreich zu sagen, dass kritische Punkte angeschaut werden und dass es kein Urteil über die Person/Betrieb ist.

Exp: hilfreich manchmal offen zu fragen, warum die einte/oder andere Massnahme nicht umgesetzt wird.

VET: wenn keine MLP-Daten ist es problematisch, keine Daten vorhanden – das sind auch die die nicht monatlich den CMT-Test machen.

Manchmal wird es gemacht aber nicht notiert, trotz vorhandene Blätter.

VET: hat eine Datenbank erstellt, mit Test und Behandlungen als visuelle Hilfe.

Zugang zur Daten wird gerne dem TA gewährleistet, manchmal TA besser informiert.

Exp: Bauer froh um Unterstützung und bezahlen dafür, auch wenn Resultat nicht immer das ideale entspricht (individuelle Anpassung)

-> Kommunikation ist essentiell, Bauer muss einsehen wieso gewissen Punkte angeschaut werden und dies nicht als einseitige Kritik interpretieren, sondern als Grundlage um das Problem einzugrenzen.

-> wenn keine MLP-Daten vorhanden sind, ist die Arbeit sehr schwierig. Auch Dokumentation von Fälle/CMT ist nicht immer vorhanden.

-> Intelligenz der Kunde beeinflusst Vorgehensweise und Intensität der Betreuung stark

Moderator: Bedürfnisse

VET: entweder bei Probleme, oder bei der Bestandesbetreuung. Mentalitätsänderung, eine höhere ZZ wird akzeptiert wenn man keine klinischen Fälle hat und nicht zu viele Therapie machen muss.

VET: ideal ist Herdendurchschnitt gut und 1-2 Millionärinnen toleriert (besser als alle hoch)

Exp: Gruyère Bonus Tank <80‘000 Z/ml, nicht mehr so sinnvoll (VET auch 1) – fraglich was es für Käsequalität ausmacht.

Roboter verboten da bei Verkürzung Zwischenmelkzeiten anderes FS-Muster.

VET: z.T. Kunden enttäuscht dass nicht immer alles behandelt wird -> nicht alle Wissensentwicklung mitgemacht, Unterschied auch zwischen TA

Exp: TA haben z.T. Angst dass eigene Schuld wenn Kuh nicht gut kommt, Tendenz ist da übermässig zu behandeln

VET: Nachverfolgung ist wichtig, wird nicht immer gemacht, wenn dann in Rahmen Bestandesbetreuung

VET: Kommunikation und viel darüber zu sprechen bringt viel und wird geschätzt -> für TA Zeitintensiv und anstrengend

Exp: wenn Bauern ein Benefit bei Dialog (und TAMV-Besuch) sehen dann auch einverstanden dafür zu bezahlen

VET: in kleine Betrieben keine TAMV-Vertrag und keine Vorrat abgegeben.

Alle: zT wird von Kantone (Kontrolleuren) TAMV-Vertrag angeordnet auch wenn kein Vorrat, was absurd ist.

VET/VET: Mentalitätsänderung auch durch die AB Reduktionsthematik, höheren ZZ werden toleriert wenn weniger klinische Fälle vorhanden sind und weniger behandelt werden muss

VET: Unterschied zwischen TA was MP und Behandlungen angeht, gewissen Kollegen haben das Wissen nicht und behandeln viel oder wollen nicht riskieren, das ein Fall nicht gut kommt und darum behandeln sie mehr. Dies verwirrt die Landwirte, da kein einheitliches Vorgehen vorhanden. Darum Info/Diskussion wichtig.

Moderator: Fragen

VET: Bio für jede Kuh Bakt und ABgramm nötig – nicht so sinnvoll. Zudem ist auch nicht vorgeschrieben wo ABgramm und mit welchen Methode es gemacht werden muss. Viel Kosten verursacht.

Exp: politische Regelungen wollen strenger sein als IP. Kein Standard vorgeschrieben für Antibiogramme!!

Moderator: Bestandesproblem hohe ZZ

VET: Vorher hat Bauer keine Strategie gehabt und Kiloweise bei TA (anderen) AB geholt.

Alle: Anwesende nicht klar, wie man als TA so viel AB abgeben kann ohne MP zu nehmen. Fraglich auch wie man die Resultate interpretiert.

VET/VET/VET MP durch LW geht nicht bei allen gleich gut, ansonsten kein Nutzen. Evt. nötig Bauern nochmals zu instruieren.

VET: diesen Betrieb auch viele FL Kühe, aber nicht alles gleichzeitig angegangen.

Exp: verschimmeltes Futter kann viele Probleme auslösen, Beispiel von Betrieb mit Schimmel in Kraftfuttersilo.

Futterqualität und Mykotoxine grösseres Problem als was eingeschätzt wird.

VET: viele Kühe mit B.licheniformis, häufig im Silo (Exp in Sickersaft)

VET: Betriebe mit gute ML und ZZ, ende Sommer plötzlich über 2 Wägungen hoch, Problem mit Mais. Frag wie häufig muss man Futter untersuchen? In diesem Fall nur 5 von 80 Kühe behandelt. Mais war frisch und wie kann man es kontrollieren? Ist man immer zu spät?

VET: bei Schweine häufig Mykotoxine untersucht, Problem das im Futter zu suchen ist, dass Verteilung nicht gleichmässig, dann Methode auch unterschiedlich. Im Ausland viel billiger.

VET: als TA untersuchen wie Futter zu wenig, auch in den Fütterungsberatungen. Bräuchten eine Strategie für regelmässige Untersuchungen von Mais.

VET: Anders bei Schweine und Mast, ohne TS kann man Futterkurven nicht rechnen.

Exp: untersuchten Mykotoxine ->nur Spitze des Eisbergs, aber was wird gemacht wenn man es findet?

VET: Ist es möglich Zeitgerecht zu intervenieren trotz Untersuchungen?

Antwort/Anmerkung Exp: Totale Pilz- und Hefenbelastung kann auch bestimmt werden, Probenentnahme und –lagerung bleibt problematisch.

VET, VET, VET: Fütterung wird nicht immer durch bzw. mit dem TA angeschaut, da besteht Versbesserungspotential

VET: Mycoplasma-Betrieb, rel. ruhig, ab und zu akute Mastitis, bei jede Mastitis PCR gemacht. Neben Mastitis auch Pneumonien, Therapie ist schwierig, Oxyttc oder Marbocyl und NSAID, keine Lokale Behandlung (sobald bewusst dass Mycoplasma ist). Besitzer versteht nicht dass man Tiere ausmerzen muss.

VET: auch Mycoplasma – harte Vierteln, nur NSAID. Selbstheilung ist bei gewisse Tiere beobachtet. Tiere wurden separiert. Positive Tiere waren ca. 5Mo tragend (vs VET alles Stadien).

Exp: bei XYZ kann man eine Milchwägung beantragen auch wenn kein Abo.

VET: Hemmstoffnachweis mit biologischem Testsystem (Brillantgrün); Snaptest rel.teuer und weniger sensitiv, Delvo für Tankmilch vorgesehen (geht aber länger).

ALLE: seriöse Betreuung/Abklärung ohne ZZ-Kontrolle ist kaum möglich

VET: häufig da wo ZZ Problem Melkberater dazu geholt da es schwierig ist. Kompatibilität von Techniker und Berater muss vorhanden sein.

Exp: Manchmal wird technisches Problem als zu wichtig beurteilt, die anderen Faktoren sind auch wichtig (Management, etc).

VET: Alternativmedizin funktioniert ähnlich, es wird ähnlich an das Problem angegangen.

Exp: unterschätzt wird auch, wie wohl sich die Kuh fühlt beim Melken und Reingehen im Stand. Stress spielt eine grosse Rolle.

Exp: Wie sind die Erfahrung mit Milchmaschinetechniker?

VET/VET Schlecht bis sehr gut, Bauern haben manchmal Mühe zu glauben, dass wenn Service ok ist dass nicht zwingend alles gut ist.

Exp: TA beurteilt melken und wichtig mit Berater gute Beziehung zu haben. Nicht wahr, dass Roboter immer fehlerfrei funktionieren.

VET: gewissen Roboter sind inzwischen alt, und sämtliche Techniker sind nicht up-to-date.

Exp: Lahme Kühe, dreckige Kühe, Stall-Comfort sind Eckpunkte. Mehrheitlich Umweltkeime.

VET: erzählt von Betrieb neu mit Roboter und 2 Ursprungsbestände, kein Pura-System und S.aureus Problem verursacht.

VET: häufig sehr dunkel im Roboterbereich auch ein Problem, die Kühe kommen nicht freiwillig.

Exp: Zusammenarbeit Melkberater/Milchtechniker/TA ist wichtig und auch hier ist offene Kommunikation sehr wichtig.

VET/VET/VET: Service gut heisst nicht per se alles gut

VET: Roboter unterschiedlich gut je nach Generation, Ort, etc

Exp: Kuh- und Stallhygiene, Stress, Comfort sind neben Technik wichtig

Exp: Was funktioniert, womit gibt es schneller Ergebnisse, oder Bauer Problem?

VET: wenn konkrete Keim oder Technik das Problem geht es gut, wenn es sich um ein Fütterungsproblem handelt schwieriger, da Kontakt/Wissen mit Beratern nicht immer einfach.

VET: Fütterung Knackpunkt, wo TA auch anstehen.

Exp: Ziel von Fütterungsberatung als TA ist gesunde Kuh, nicht hohe ML oder Futter zu verkaufen.

Milchinhaltstoffe, BCS, Kotwaschen als Hilfsmittel. Mit Agrideaprogramm grobe Abschätzungen von Rationen. Wichtig auch zu fragen, wie es die andere Seite sieht, Bauer und/oder Fütterungsberater.

VET/VET: Konkrete Probleme (Keim, Technik) einfacher anzugehen als z.B. Fütterung

Exp: Unterschiedliche Blickwinkel TA und Berater

VET: als TA eingrenzen des Problems, aber für Details Berater. Betreffend ZZ, auch nützlich das Formular der Kontrolle anzuschauen und Anmerkungen/fehlende Untersuchungen zu identifizieren.

Dippmitteln und gew. Desinfektionsmitteln coselektionieren für Resistenzen, Studien über Status quo sind im Gange (Exp).

Exp: wichtig, dass desinfizierende Mittel drin ist, aber auch Sauberkeit des Bechers beurteilen. 2-Komponente darf nicht der Kälte exponiert werden. Chlor im Dippmittel und Kalk wirken irritierend auf Zitzen und die Wirkung der Chlorverbindung wird aufgebhoben. Im Moment keine klaren Strategien betreffend Dippmitteln. Verkauf seitens TA ist stark vermindert. Suissemed-Nr heisst nur, dass Wirksamkeit überprüft wurde, nichts mit Zulassung zu tun.

VET: Dippmittel mit Peressigsäure gibt es auch, rel. gute Erfahrungen als Alternative zu Jod.

Exp: auch ein Provet Dipp mit 2-Kompnenten (Lactose und NaCl, dass dann zu einer aktiven Chlorverbindung werden soll) Wirkung?

ALLE: Viele Dippmitteln vorhanden, Unklarheit herrscht über Vor/Nachteile der jeweiligen Mitteln

Zusammenfassend von allen geäussert:

- Tierhalter muss mitziehen
- Fütterung muss im Detail angeschaut werden, wenn dort Problem ist es komplexer

VET: Trockenstellen wenn CNS ¼ dann AB in alle Vierteln?

Exp: im Moment keine Evidenz dass Sinn macht nur einzelne Vierteln antibiotisch trocken zu stellen.

S. uberis häufig chronisch und klinisch /subklinisch, andere Stamm als akute? -> in Routineuntersuchung nicht untersuchbar, aber verschiedene Stämme bekannt. Kontrolle nach Behandlung macht Sinn.

VET: Behandlung ist manchmal nur einige Mo effektiv, dann kommen sie wieder und bleiben in höhen ZZ-Bereich.

Feedbackrunde:

VET: Fütterung mehr ansprechen statt nur auf Melken zu konzentrieren

Exp: TAMV-Besuche in andere Licht, positiv

VET: Leistungsverrechung (Beratung) braucht noch Anpassung und Bedürfnis da um Preis festzulegen (VET: 200Fr pro h muss man haben um alle Kosten zu decken)

VET: fest auf Tier selber konzentriert, nächsten Besuche Melken und Fütterung anschauen

VET: mehr mit Fütterung machen, einfach mal anschauen. Nicht-Herdebuchbetriebe dazu bringen ZZ zu kontrollieren.

VET: Fütterung, Melkanlage, Desinfektion als Themen für anderen Arbeitskreise. TA verrechnen zu wenig.

VET: Mycoplasma als mögliche Mastitisursache wahrnehmen

VET: Dippmittel Kältefehler bei 2-Komponente und Chlor/Kalk Kombination als Problem nicht bewusst.

Protokoll: Mastitis

EXP: Wie ist das Grundvorgehen bei Anruf in der Praxis „akute Mastitis“? Wann wird man als TA angerufen, Einteilung der Mastitiden etc.?

VET: Fieber >40° und Viertel hart bedeutet für meine Bauern akute Mastitis und rufen mich an (bei 39.2°, und Viertel verändert warten viele noch und probieren mit Salben und ausziehen). Generell bei einem Anruf wegen Mastitis ist das Spektrum sehr breit was der Grund ist. Von CMT positiv bis Milchprobe bringen sehr unterschiedlich und stark von Bauer abhängig, NSAID i.v. immer wenn Euter verändert, auch wenn noch kein Fieber

EXP : Es gibt ein ganzes Spektrum von Anrufanamnesen, je nach Tierhalter. Wie ist es mit Medikamentenabgabe?

VET bejaht: Gentapen

EXP: Nur in milderen Fällen?

VET: Nein, initial immer Gentapen

VET: Bei mir gibt es keine Eutertuben-Abgabe in TAP, Vorgehen Anamnese: Kuh mit Viertel, immer nachfragen ob akut oder nicht, dort wo akut: Einteilung 1.-3. Grades dann je nach dem therapieren

1. Grad: neu angefangen tlw. nur Entzündungshemmer
2. Grad: Initialbehandlung und 5 Tübli abgeben
3. Grad: Fieber: systemische AB, Sepsis: je nach dem schon an den Tropf

Milchprobe (MP) bei allen, halte aber nichts von den Schnelltest, Bei S.aureus akut 3. Grades, telefoniere ich nach wie es der Kuh geht

EXP: Einteilung der Grade:

1. Grad = veränderte Milch, etwas schwerer Viertel
2. Grad: bis 39.5° Temperatur
3. Grad: Kreuzviertel, hohes Fieber, vermind. AZ evtl. Zeichen von Toxämie

VET: In Baar: Anrufanamnese sehr breites Spektrum. Von Fetzen bis 40.5° und nur noch Brandwasser gibt es alles. Initialbehandlung mit GP, bei Fieber immer mit Entzündungshemmer (für 3. Tag Abgabe an LW), bei akutem Viertel mit hohem Fieber Vetagent, häufig auch mit Mamyzin

VET: Ich finde Mamyzin eher bei chronischen geeignet, sonst Trimethazol

Wird von anderen bejaht

VET: Synulox und Gentapen geben wir ab, vor allen Dingen Synulox

VET: Neo- M Abgabe ist bei uns die Standardtherapie für „nicht schlimme Fälle“, Advocid von Anfang an bei ganz schlimmen Fällen, und noch Ca Infusion und NSAID

VET: Ich mache eine Mischung zw. Vet und Vet: Vorrat Gentapen/Synulox; Enroflox bei ganz schlimmen Fällen systemisch, Milchprobe nehmen wir immer, immer NSAID sobald Viertel hart auch wenn noch kein Fieber (auch bei chronischen)

VET: Bei Coli nur noch Sulf- Trimeth., so umgestiegen und ist sehr gut gelaufen (Trimethazol und Borgal)

VET: Diskussion geführt ob nur noch Sulfonamid Trimethoprim, aber Cornelia hatte einige verschleppte Fälle gesehen, deshalb nicht

VET: Jahrelang Borgal jetzt auf Fluorquinolon i.v., NSAID geben wir immer, Milchprobe bringen bei verschleppten, Synulox geben wir ab. Bei akutem Viertel mit hohem Fieber: einmal alles, wenn Gram - behandeln wir bei guten Bauern nur systemisch (und lokal). Bei Betrieb mit bekannten chron. Klebsiellen: mit NSAID behandeln und auf längere Zeit ausmerzen

EXP: Literatur Empfehlung: klare Unterscheidung zw. mild, mittelgradig, schwergradig (Ohren hängen lassen, rote SH, toxisch), Grundsätzlich für schwere Fälle hat intramammäre Behandlung keine Evidenz dass es nützt, aber es gibt hohe Evidenz, dass systemische Therapie , inkl. Infusion + Fluorquinolon Klinik verbessert in schweren Fällen, ABER nur für toxisches Limit oder Bakteriämie (ganz wenige % kommen überhaupt so weit), dort macht es Sinn bakterizid,

schnellwirkendes AB zu nehmen, Moderate und mildere Fälle mit Trim-Sulfa sehr gute Erfolge mit hoher Dosis 48mg/kg über mehrere Tage all 12h, bei nur veränderter Milch, wenige Symptome: nur NSAID

VET: Nicht alle Bauern machen das mit, viele wollen Tübli

EXP: Das ist klar...

Schnelltests sind gemacht für grosse Betriebe, es wäre aber eine Möglichkeit dass man intramammäre Therapie erst anfängt wenn gram +

Bei wie viel % wäre diese Therapie möglich? Wie ist sonst das Gefühl mit "Nichts in das Viertel geben"?

VET: Ca. bei 20-30% der Kühe möglich (grosse Betriebe, aber wenig Bauern)

VET: Denke kommt nicht gut an, habe es noch nie probiert

VET: Chronische schon, sonst aber nicht bei einzelnen Vierteln

VET: Anfangs i.v. und Tübli dann nur abgeben falls am Abend nicht besser oder Landwirt hat „schlechtes Gefühl“. Dann kann er es selbst noch geben. Häufig werden sie so nicht eingesetzt, da Bauer selber darüber nachdenken muss.

VET: Wir behandeln bei chronischen nach Milchprobe, wenn wir wissen was es ist, nur mit Tylan ohne intramammäre Therapie (Tylosin empfindlich getestet, ABG mit Plättli, AB 3d: zuerst i.v., dann abgeben (3x 33ml), gebe gerne Initial 40ml). Im akuten Fall immer intramammäre Therapie.

VET: Je nach Bauer: sind sicher noch sehr wenig, aber versuche es immer mehr anzureissen

VET: Bei vielen schon Diskussion geführt... sinnlos

VET: Bei gram + Verdacht immer lokal, aber bei chronisch dann systemisch?

Kurze Diskussion über Tylan + Pencillin = zeitabhängig, Fluorquinolone= Dosisabhängig

VET: Vetagent? Gentamicin braucht niemand?

EXP: Gentamicin: Nur in akuten Fällen: wenn Blutmilchschranke offen nützlich, wenn aber Fibrinkoagula im Euter sind wirkt es nicht gut. Studie zeigt: Borgal: in Mastitismilch die wenig verändert ist wirkt es besser als bei normaler Milch oder ganz stark veränderter Milch. Bei stark dehydrierten daran denken, dass Gentaausscheidung über Niere erfolgt, Studien zeigen: Fluorquinolon am wirksamsten: aber nur bei ca. 5% der Fällen indiziert

EXP: Ausmelken mit Oxytocin? Standard?

VET: Eher wieder weniger, wirkt nicht immer

VET: Initialtherapie: Oxy spritzen, dann bereit machen und ausmelken lassen

VET: Früher Coli alle 2-3h ausmelken lassen

EXP: Studie sagt es bringt nicht mehr so viel, es gibt aber auch Unterschied zwischen regelmässig Viertel ausziehen und 4x tgl. Euter leeren

Studie zeigt: Uberis mit Oxy ausmelken macht nur dass sie besser haften können

VET: Frage: Ich verwende als NSAID Flunixin, weil ich dachte, das sei ein Toxinfänger, der Chef findet das aber zu teuer. Und es steht nur in der Packungsbeilage von Metacam und Metacox das sie Toxinfänger sind, bei Flunixin nicht: ist das alt hergebracht?

EXP: Ja, hat sich sicher eingeschlichen, da Flunixin viel älter als Metacam etc. (Vet)

VET: Ich brauche Metacam

VET: Ich brauche Ketoprofen

EXP : Metacam ist langwirksam, Keto nur kurzwirksam, Flunixin je nach Zustand nach 24h wiederholen

VET: Wir benutzen Metacam oder Fluni, Initial 20 ml Fluni (1.1mg/kg)

VET: In Literatur steht 2.2mg/kg für Rinder (1.1mg/kg sei Pferde Dosierung)

VET: Wir nehmen bei Metacam 15-18ml

EXP : Chronische, wiederaufflammende nur mit NSAID, da häufig schon viele AB hinter sich und anscheinend nichts nützt

VET: Flunix – Metacox ist man sich nicht einig?

EXP : Fluni für Mastitis zugelassen, deshalb übernommen von Metacox

VET: Laut Studie soll Konzeptionsrate bei Kühen mit Fluni behandelt (bei Mastitis) besser sein ;)

EXP: Hattet ihr schon mal das Problem, das eine an Magenulcus gestorben ist?

Im anderen AK: Fallbeispiel von 16d Dolovet (chron. Schmerzen mit Unfall) an Tag 14 war Kuh noch fit, dann am Tag 16 Magenulcus. Aber Kuh hatte auch Schmerz- Stress und evtl. waren nicht nur die NSAID dafür verantwortlich

Von der Aussage: „>1mal NSAID macht Labmagenulkus“ bin ich nicht überzeugt

VET: Am FiBL gab es mal eine Studie: 10ml Dexa, dann 5d Meloxicam, da sei nicht mal etwas passiert

VET: Warum wird Cortison so verteufelt? sei nicht gerechtfertigt?

EXP : Früher auf Praxis habe ich auch Dexamethason eingesetzt. Heute aber nicht mehr, nur noch NSAID

VET: Gibt jemand Dexa intramammär?

Viele: Nein weil Synulox hat Pred drin

VET: Ich gebe 2ml in Zisterne bei starker Schwellung nach Zitzenverletzung

VET: Wie viel Liter Infusion geben?

VET: Ich drenche: Wasser + Omasin + Eigenrezept von Bauer (nur bei 2 Bauern Infusion)

VET: 45L drenchen : Wasser + Omasin+ Pansenaktiv oder Drenchlakt;

Bei Infusionen: 10 L Infusion, und nochmal 10 L abgeben (10L in 24h)

VET: Bei Infusion lasse ich eine isotone Lösung im Strahl volllaufen, so viel wie möglich, also sicher viel mehr als 10l. Ich drenche nur mit Wasser, wenn überhaupt, keine Zusätze wie Omasin etc.

EXP: Ja genau, isotonische Lösung so viel geben wie möglich, beim Drenchen: so viel wie möglich, „Nicht Drenchen im Liegen“ ist ein Ammenmärli

VET: Ca- Infusion: als Toxinfänger und zur Gefässabdichtung, wird separat zu anderer Infusion gegeben

Kurze Diskussion über Festliege- Therapie: mit Phosphorsupplementation auch über Drenchen + Bovikalk P Boli(Vet), Paste (Vet)

VET: Euter spülen?

EXP : Nur bei offener Amputation

VET: Wir machen das manchmal noch

EXP: Wer macht das mit Glucose?

VET: Nur wenn Bauer es wünscht: Kristallin Peni und Genta auflösen und mit Infusion hoch in Euter, Ich bin davon nicht ganz überzeugt

VET: Solutio Parabene nur bei Gentapen, dort wo ölige Medis wird das nicht gemacht

VET: Frage: Vit. E/Selen toxin binder? Tocoselenit bei toxischen Kühen? Gibt es dazu Studien?

EXP: Euterabwehr lokal besser wenn Selenmangel und Selen gegeben wird. Wenn kein Mangel vorhanden bringt es aber nichts. Rinder haben jedoch häufig einen Mangel

Kurze Diskussion zu Kälber und Selen: alle geben IMMER Selen bei Kälbern

EXP: Sicher nicht schlecht, man schadet bestimmt nicht

VET: In akuten Fällen, Baytril intramammär 5 ml?

EXP: Kann man i.v. geben weil es ja gut ins Euterpenetriert, Formulierung ist genau für das gemacht, und nicht für ins Euter, ich rate davon ab

VET: Ich habe einen Landwirt, der im Bestand Staph. aureus akute Mastitiden mit blauen und kalten Eutern hat

EXP: Habt ihr schon über Impfung nachgedacht, das wäre eine geeignete Indikation

VET: Ja, aber der Bauer möchte nicht

VET + VET: Kennen das nur bei Schafen mit diesen akuten S.aureus Vierteln

VET: Soll man bei akuten Streptokken Mastitiden länger nachtherapieren als bei akuten Coli Mastitiden?

VET: Wir therapieren 5d nach

EXP : Ich unterstütze das, bei Mastitiden mit sehr gut adaptierte Keime ist eine längere Therapie häufig sinnvoll

VET: Hefenmastitis? wer macht was?

EXP : Empfehlung: NSAID und ausziehen (ist selbst aber nicht sehr begeistert davon), Für die Praxis wäre das Wiedereinführen von Mastimycin super, Panalog ist aber ABSOLUT nicht zu empfehlen

VET: Ich habe gehört, dass Imaverol gut helfen soll. Ist aber momentan nicht lieferbar.

VET: Wir geben Azidosan (Kälberinfusionslösung) um pH zu verändern intramammär, wird auch abgegeben, nach jedem melken 50 ml, reicht für 5d

EXP: Werdet ihr nach Viertel Veröden gefragt von euren Bauern? In der Romandie ist das ein Thema, es werde über Iodtinktur und Akradinorange diskutiert.

Alle verneinen

VET: Ich verstehe nicht warum das helfen soll.

EXP : Die haben lieber ein totes Viertel als eine Mastitis, die immer wieder kommt.

EXP: Wir hatten einen Fall mit 2 Kühe mit atypischen Mykobakterien. Die wurden dann in der Melkmaschine (Sammelstücken) nachgewiesen. Die Kühe waren klinisch etwas angeschlagen, hatten aber keine typischen Viertel, PTA hat nichts gefunden, CMT war positiv

Die Diagnose konnten wir stellen, weil wir die Bakteriologie speziell gefragt haben lange zu bebrüten, wachsen auf Blutagar

VET: Wie therapiert ihr Cereus?

EXP: Häufig zu spät, wenn es nur eine milde Mastitis ist, und er wird identifiziert, dann ist es nur eine Kontamination

VET + VET: Tropf, antitoxisch behandeln, versuchen Kuh zu retten

Das Fazit Expertenrunde: Es ist empfehlenswert ganz schlimme Fälle nur noch systemisch nicht mehr intramammär zu behandeln!

Erfahrungen untereinander austauschen

1. E. Coli
2. Klebsiella spp
3. S. uberis
4. Streptococcus spp
5. S.aureus
6. Bacillus cereus
7. Hefen

1. Anamnese
2. Therapie
3. Therapieerfolg

Mod: Wir streichen die Keime, die wir schon ausführlich mit Exp besprochen haben, die anderen könnt ihr ja noch kurz in 2er bzw. 3er Gruppen durchsprechen.

VET: Ich finde das unnötig, weil ich bei akuter Behandlung gar nicht weiss welcher Erreger die Mastitis verursacht. Deshalb behandele ich initial alles gleich.

Oder man macht es so wie VET und nimmt erst eine Milchprobe und entscheidet dann. Aber das machen die wenigsten.

VET: Das Milchsekret, gibt mir noch einen Hinweis auf den Erreger

VET: Findet das unzuverlässig v.a. bei Coli

VET: Ich gehe nur bei Pyogenes nach Sekret, sonst nicht

VET: Denkt so wie es Vet macht, macht es eher Sinn Erreger für Erreger zu besprechen, weil er immer NSAID gibt und dann MP

VET: Initialbehandlung immer gleich und dann MP, dann evtl. mehr Tübli oder andere auf anderes Antibiotikum umschwenken

Diskussion über Bescheid geben: ob Telefon oder wie sonst? wie Beratung verrechnen? Vet meint verrechnet nichts, weil es auch niemand bezahlt. Bei akutem Viertel werden so viele Medikamente gegeben, dass es eh so teuer wird, dass Beratung mit inbegriffen ist!

Mod: Akute Mastitis für alle abgehackt?

Allgemeine Zustimmung

Feedbackrunde

VET: Behandlung von akuten Mastitiden in Wandel im Vergleich zu früher, Schlaue Idee mit Hefemastitisbehandlung: Azidosan

VET: Wird versuchen Vetagent bei akuten Mastitiden zu vermindern, eher Borgal brauchen,

Paar Sachen gerne besprechen In Praxis, Findet Hefegeschichte toll mit Azidosan, Ca mehr im Bewusstsein für toxische Kühe

VET: Nur NSAID bei nicht ganz schlimmen Fällen: bei einigen Betrieben den Mut zu haben, Besprechen mit Drenchen und 10l Kanister aufhängen (momentan nur 1-2L im Sturz von Glucose und Ca, aber nicht wirklich Volumen): Anschaffungsgespräch mit GL in Praxis

VET: Ich werde meine Therapie für akute Mastitiden anpassen und Borgal höher dosieren

VET: Azidosan für Hefemastitis

Protokoll Trockenstellen

Mod: Flowchart von Praxis Müller aus Deutschland verteilt

Vet: Flowchart von ihrer Praxis verteilt

Vet: Praxislabor nach dem Diagnostik Kompass der Firma Boehringer, bis vor 2 Jahren Antibiotika-Plättchen für Resistenztest, möchte es evtl. wieder einführen

Vet: Richtig für Resistenztest wären die Mikrodilutionsplatten

Vet: dies machen die Labore aber auch nicht

Vet: Praxislabor nach dem Diagnostik Kompass der Firma Boehringer

Alle: Labor in St. Gallen sehr gut für Milchdiagnostik

Vet: Wie kontrolliere ich, ob selektives Trockenstellen gut funktioniert?

Aussage vom Landwirt muss nicht der Realität entsprechen

Bei hoher Milchleistung (9000-10000l) fängt Risiko für selektives TS an.

schlechte Rückmeldungen mit Orbeseal bei hohen Leistungen

-> Evtl eine Milchbakt vorher machen?

Erfahrungen von Experte (NOP Betrieb)

Milchkuhbetrieb, Bio, 48ha, 55 Milchkühe, Brown Swiss, 8000l Leistung

Seit 8 Jahren ohne Antibiotika und seither 1 Trockensteller bei einer 10000l Kuh gebraucht.

Homöopathisch seit 2005, seit 3 Jahren Antibiotika frei, auch die Kälber

1. Management, Klima und Fütterung sind das A und O
2. Homöopathie braucht Zeit und Geduld
3. Wichtig: Früh erkennen und reagieren

4. Die Bauern müssen mehr Verantwortung übernehmen und die Zusammenarbeit mit dem Tierarzt ist wichtig
5. Gesamtherde ist entscheidend, nicht die Einzelkuh
6. Zucht ist ein weiterer Baustein

- Orbeseal: gebraucht er nicht (Waschen der Melkanlage zu aufwendig)
- Verein gegründet: IG neue Schweizer Kuh → Zukunft?

Frage: Besamst du eine Kuh mit hoher Milchleitung später?

Exp: Nein, Aufnahme am besten wenn Kuh mit 60Tg stierig ist, Kuh vor 100 Tg belegen, da bessere Aufnahme

-> Optimale Energieversorgung gewährleisten

Frage nach Einsatz von Entündungshemmern:

Exp: Nein, Entzündungshemmer setzt er nicht ein

Wenn er etwas „konventionelles“ einsetzt, ist es Calcitat und Phosphor

Bestandesbetreuung ist wichtiger geworden und muss verkauft werden!

Doch: Wer zahlt für Beratung von Tierärzten?

-> will der Bauer nicht bezahlen oder traut sich der TA nicht das zu verrechnen?

Fazit: Wenn durch die Beratung etwas bewegt wird, dann bezahlt der Bauer auch

Mod macht das Angebot einen AK extra zu diesem Thema (Kommunikation) abzuhalten.

Argumente für/gegen AB freies Trockenstellen

Argumente dafür:

1. Anzahl der Galt-Mastitiden trotz antibiotischem Trockenstellen
2. Resistenz Problematik
3. Günstiger
4. Image Schweizer Landwirtschaft
5. Keine Chemotherapeutika in die Umwelt
6. Nutzen oft nicht geprüft
7. Weniger AB-Einsatz
8. Genauere Tierbeobachtung vor/nach Trockenstellen
9. Gesetzlicher Rahmen
10. Vielfach unnötig

Argumente dagegen:

1. Bestandesproblem Zellzahl
2. Bestandesproblem mit unfähigem Besitzer

3. Sicherheitsgedanke
4. Betriebe mit schlechtem Management/schlechter Hygiene die keine Aussicht auf Besserung haben
5. Ausheilung von infizierten Kühen schlechter
6. Staph. aureus Bestandesproblem

Fazit: Jedes Tier sollte einzeln beurteilt werden

Vet arbeitet in ihrer Praxis nach einem 7 Punkte Plan

Einteilung Problembetrieb vs gesunder Betrieb?

Es entsteht eine Diskussion ob diese Einteilung besteht, oder nicht.

Fazit: es kommt doch auf den Betrieb an, ob mit oder ohne Antibiotika Trockengestellt wird, also gibt es auch eine Einteilung in Problembetrieb/ gesunder Betrieb

Exkurs zum Thema Therapie:

Man muss den Mut haben, den Bauern zu überzeugen, dass er eine 40Grad Celsius Kuh nur ausmelkt

Erfahrungen von Vet und Exp., dass es durchaus funktioniert!

Feedbackrunde

- Jeder macht es ein wenig anders

- Es machen es mehr oder weniger alle gleich

- nichts Neues gelernt , es läuft auf das gleiche raus.

- selektives Trockenstellen -> reine politische Entscheidung, beruht nicht auf wissenschaftlicher Erkenntnis

Prorokoll: Trockenstellen

Expertenrunde

Vet: Kriterien um eine Kuh ohne Antibiotika Trocken zu stellen:

- Schalmtest
- Gesunde Kuh/Konstitution der Kuh
- Keine Probleme im Betrieb
- beim Einsatz von Zitzenversiegler extrem sauber arbeiten

->Beobachtung des Bauern spielt eine entscheidende Rolle!

EXP Beim Erarbeiten von Richtlinien muss man eine Mischrechnung machen, Frage der Sensitivität und Spezifität von Zellzahlmessung bezüglich IMI. Ganz klar internationaler Konsens: gesundes Euter hat <100 000Zellen/ml da wie fast alle biologischen Parameter auch die Zellzahl normalverteilt

ist, nehmen wir da noch eine bis zwei Standardabweichungen als Toleranzbereich hinzu, wie das auch für andere analytische Laborparameter gemacht wird.

Andere Länder haben auch andere Bonus bzw. Malussysteme…..

Vet: Kriterien um eine Kuh mit Antibiotika Trocken zu stellen:

- 2 Kreuz bei Schalmtest bei einem Viertel
- >150000 Zellen -> Milchprobe nehmen
- Sekret adspektorisch/Palpatorisch verändert
- Generelles antibiotisches Trockenstellen -> weniger Behandlung in Laktation

Exp: gutes Argument!!! Wenn da eine klare Strategie dahinter ist, sollte das auch beim Vollzug durchkommen.

Vet: Konkreter: Zellzahl - Grenze

Exp: Gesetzlich gibt es eine klare Grenze von 100.000 Zellen. Hat eine Herde eine ZZ von unter 100.000 gilt diese Herde als Eutergesund.

Mit der Zellzahlgrenze von 150.000 wollte man mehr Spielraum geben. Kuh mit einer Zellzahl von unter 100.000 gilt als gesund, über 100.000 gilt sie als krank

Zellzahlen differieren von Betrieb zu Betrieb und hängen auch stark vom Empfinden des Landwirts ab. Man muss als Tierarzt mit entscheiden, mit welcher Zellzahl-Grenze jeder einzelne Betrieb am ehesten zurechtkommt. Ein Betrieb, der bei 250.000 anfängt, muss man Schritt für Schritt langsam zurückfahren. Es wird nie Richtig/ Falsch geben. Man muss das auf Betriebsebene anpassen.

Vet hat einen Betrieb der ZZ von 280.000 hatte. Er stellt diesen nun konsequent mit Antibiotika und Orbeseal trocken. Liegt nun bei 80.000. Er spart lieber nicht bei den TS, sondern bei AB-Einsatz im klinischen Bereich.

Vet merkt an, dass das Gesetz ja nur sagt, dass die Verantwortung nicht mehr beim Landwirt liegt, sondern nun bei uns. Und wenn der TA findet, dass der Einsatz von AB-TS gerechtfertigt ist, ist das so OK.

Vet: Einzelne Betriebe müssen individuell berücksichtigt werden. Die Zusammenarbeit mit dem Betriebsleiter ist wichtig!

Vet: Bei einem unerreichbarem Ziel ist man immer unzufrieden. Ziel müssen erreichbar sein!

Exp: Man hat sich auf TS gestürzt, weil nicht jede Kuh einen TS braucht, sprich es zu einer prophylaktischen Anwendung von AB kommt. Resistenzen können auch während der Galtperiode entstehen.

Vet fragt, ob es noch AB TS mit kritischen Wirkstoffen gibt. Die Frage wird verneint (keine Präparate unter kritische Wirkstoffe auf dem Clinipharm)

Vet: Sterile Milch?

Exp: Milch ist nicht steril, jedoch ist bei deiner gesunden Kuh mit einem Standard-Inokulum nichts nachweisbar.

Vet: Bei uns gilt bei weniger als 5 Kolonien bildenden Einheiten (auf Schafblutagar), das "nichts" ist.

Vet: Staph aureus:

Exp: Behandlung ist nicht das wichtigste. Heilungschancen sind sehr gering, evtl. über Galtzeit möglich sonst Metzgern. Er/sie rät nicht zur Behandlung.

Vet: Was mache ich mit Herde, in der die meisten Kühe staph haben?

Exp: Priorisieren. Diejenigen behandeln, bei denen am ehesten mit einem Therapieerfolg zu rechnen ist: junge Kühe, keine Mastitis-Vorgeschichte, ein Viertel betroffen, frische Infektion, Ende der Laktation, danach diejenigen bei denen es sich evtl. auch noch lohnt und als letztes diejenigen, die schon mal staph Befund hatten.

Man könnte auch alle miteinander behandeln und evtl. früher Trockenstellen.

Therapiekontrolle mit PCR (Proben können gepoolt werden, dieses mit Labor absprechen)

Melkzeug nach 10 Kühen wechseln, die Ideen/Kreativität vom Landwirt fördern!

Vet alter Chef hat Staph aureus mit Hefen "behandelt". Danach war S. aureus weg

Vet: Eine bestehende Krankheit mit einer anderen "behandeln".

Vet: Resistenz bei Trockensteller:

Exp: Auch beim Einsatz von TR können Keime selektioniert werden => „Normalflora“ (Absprache mit XYZ) sicher positiv: lange genug therapeutischer Spiegel

Vet: Antibiogramm bei Reserveantibiotika ist nachvollziehbar...aber nicht bei Penicillin...Tier schon Tod bis Antibiogramm kommt...

Exp: Antibiogramm auch nicht bei jedem Keim und immer empfohlen. Bei akuter Masitits muss ganz klar sofort behandelt werden, MP zur Seite legen und evtl. noch analysieren lassen, bei schlechtem Ansprechen und Coli und Klebsis würde ich eins machen lassen. Bei subklinischen Mastitiden v.a. bei CNS => Auswahl Trockensteller!!

Frage von Exp: Wie behandelt ihr eine akute Mastitis?

Vet und Vet: Marbocyl IV

Exp: Fluorchinolone verteilen sich halt gut im Euter

Vet: Pen/Gen lokal, früher Marbocyl , jetzt Sulfonamid/Trimethoprim IV

Exp benutzt Sulfonamid/Trimethoprim IV, NSAID, Flüssigkeitstherapie, keine lokale Antibiose

Vet merkt an, dass Fieber früher oft als Lebensfeuer=Energie galt, ist die Entzündung heilsam, oder nicht? "Kocht" die Entzündung gut? Ob man die Kuh stehen lassen kann ist abhängig vom Landwirt und von der Kuh, die Fieberhöhe spielt keine Rolle. Entzündungshemmer (wegen den Schmerzen und der Entzündung, NICHT wegen dem Fieber) spritzen und eine Milchprobe nehmen.

Der Betriebsleiter muss es gut einschätzen können, und man muss von Fall zu Fall neu entscheiden.

Vet: Weideaustrieb hat auch starken Einfluss auf die Zellzahl -> Relevanz?

Exp: Relevanz sehe ich kuhseitig, Austrieb kann ein Stressor sein und ist immer noch mit Fütterungswechsel verbunden => Immunsystem evtl. kompromittiert.

Vet sieht E-Coli Viertel bei Kurzrasenweide

Exp: Im Hochsommer können Lästlinge und Wetter Stressoren sein

Vet: Eine Problemkuh ist eine Problemkuh -> hier nützen Antibiotika Trockensteller

Exp: Korrekt, kann man auch grad sein lassen mit AB zum TR Schlachtung vorsehen!

Exp: Obwohl der Landwirt meint, diese explodieren irgendwann, tun sie es meist nicht

Vet: Nein, es explodieren die, die mit ZZ niedrig sind

Exp: Vermutlich können diese nicht schnell genug die Immunzellen mobilisieren.

Vet Oder bleiben in der Herde zu viele Kühe mit einer schlechten Eutergesundheit, wenn wir mit Antibiotika-haltigen Trockenstellern arbeiten?

EXP Ich denke es sind v.a. die chronischen, die wir besser im Auge behalten sollten und nicht wiederholt therapieren sollten.

Vet: Genetik ist ein wichtiger Punkt -> Robuster

Exp: YES!

Exp: Beispiel von Norwegen: die haben mit Genetik viel geschafft, jedoch auch zur Lasten der Milchleistung.Die Kühe können die Keime besser eliminieren (S. uberis wird z.B. nicht therapiert). Jedoch muss man auch bedenken, dass die Keime dort wahrscheinlich andere Genotypen sind, als wir sie in der Schweiz haben

Vet: Reine Penicillin Trockensteller gibt es nicht...sind immer noch Aminoglykoside drin

Auf Cloxacillin sind viele resistent...umstellen?

EXP. Pen-Aminoglycosid ist eigentlich eine ganz günstige Kombination, damit erwischt man sehr gut auch die CNS!! Alternativ kann auch Amoxicillin-Cloxacillin verwendet werden, wirkt auch meisten gut gegen CNS Und Uberis….

Vet: Was machen bei Cloxacillin Resistenzen bei Staph?

Exp: Man ist abgedeckt mit Pen-Aminoglykosiden.

Vet: Werden im Labor wirklich die im Feld eingesetzten AB benutzt um Resistenzen zu testen?

Exp: In der Bakteriologie Bern werden momentan MHK Antibiogramme entwickelt, die nur noch die AB, die im Feld eingesetzt werden können getestet werden. Das soll auch von anderen Laboren in der Zukunft übernommen werden.

Vet: KNS:

Exp: Wenn bei einem Betrieb die Immunkompetenz der Kühe kompromitiert wird und/oder ein anderer Staph sich aufgrund seiner Eigenschaften etablieren kann gibt’s auch mit dieser Keimgruppe eine unbefriedigende Tankzellzahl. Die KNS leben auf der Zitzenhaut und im Strichkanal, deshalb ist die Integrität des Strichkanals auch entscheidend.

Vet: hat man Zahlen, ob man KNS über Galtzeit heilen kann?

Exp: Momentan laufende Studie aus Kanada in der sie einzelne Viertel AB trockenstellen. Und in Holland läuft eine Studie ob man bei KNS AB trockenstellen soll.

KNS erwischt man gut mit AB Trockenstellern. Ist meist ein opportunistischer Keim

Also ja, bei KNS AB trockenstellen!

Vet merkt an, dass KNS hohes selbstheilungspotenzial hat.

Exp behandelt nie während der Laktation. Nach 3 Wägungen hat sich ZZ meistens wieder normalisiert.

Vet: Ganze Euter oder einzelne Viertel behandeln?

Exp: Bei Staph: Wenn 1 Viertel betroffen steigen die Chancen auf über 50%, dass andere Viertel auch betroffen, möglicherweise auch weil betroffene Zitzen sehr nahe beieinander sind und so eine Ansteckung stattfinden kann, evt. Über Melkzeug Stichwort Vakuumschwankungen und Respray.

Vet: Was machen mit Problem-aber Lieblingskuh?

Vet: Ohne AB Trockenstellen und ohne Orbeseal

Vet: Bringt man Strep. Uberis Kühe mit Penicllin-Aminoglykosid TS über Galtzeit?

Exp: Kombipräparat, Aminglycosid kaum Wirkung auf Strep nur Pen. Heilung möglich aber nicht super, wenn chronische Infektion.

Vet: Melkreihenfolge beachten ist da besonders wichtig. Und den Landwirt fragen: Magst du die Kuh behalten, oder in der ZZ runtergehen? Eine euterkranke Kuh bleibt mit oder ohne AB-Trockensteller eine euterkranke Kuh. Bei Staphylokokken habe ich gute Erfahrungen mit der Nosodentherapie gemacht. Milch vom Betroffenen Viertel mit 70%igem Alkohol vermischen und stehen lassen. Dann aufpotenzieren und per OS eingeben. Über gesamte Galtzeit.

Vet: 1 Viertel Trockenstellen, wie genau?

Exp: Kennt viele die das erfolgreich machen, Hemmstoffuntersuchung machen, da AB nicht ausgemolken!

Vets: Ein normales Tübli (Penicillin) hoch und dann stehen lassen

Milchrückgang unterstützen

Exp: Taritral hilft (Salbei)

Feedbackrunde

Höhepunkte des Abends:

- durch den Anfang wurde Struktur in den Abend gebracht
- es gab konkrete Antworten, Ergebnisse
- Wissen von Exp bzw. die Grenze ihres Wissens
- es ist uns gut gelungen uns dem Problem zu stellen
- spannende und fruchtbare Diskussion

- es wurden mehr Fragen beantwortet
- jedes Thema wurde abgerundet, zu Ende gebracht
- Exp hat jeden Standpunkt ernst genommen
- super Experte
- man liegt nicht ganz falsch in dem was man tut
- das Miteinander, wir haben uns angehört
- Wir sind ein Tropfen auf dem heissen Stein, die Kunst ist wie kann man wann was einsetzen
- Es konnte was mitgenommen werden
- Gruppe war kleiner
- Die Diskussion war auf einer guten Höhe, Themen wurden differenziert angegangen

Tiefpunkte des abends

- Kopfweh
- Am Anfang haben wir "was auf den Kopf" bekommen mit der Standortbestimmung

Protokoll Mastitis

Échanges d'expériences personnelles.

On forme des groupe de 2 -> vet et vet (A), vet et vet (B), vet et vet (C).

a. S.agalactiae :

Groupe A :

- Anamnèse : CMT, mammite clinique, germe hautement contagieux
- Traitement : Pénicilline dans le quartier atteint. -> Les autres groupes font plutôt Pénicilline dans toute la mamelle. En plus de ça, ils font un contrôle bactériologique après le traitement (délai d'attente + 10 jours = 3-4 semaines)
- Succès de la thérapie : Bon

exp conseille aussi de faire à toute la mamelle et pas seulement sur le quartier atteint. Elle/Il dit qu'il faut faire attention car certains germes sont zoonotiques et se transmettent de l'homme à l'animal.

b. S.uberis :

Groupe C :

- Anamnèse : Le groupe s'intéresse au troupeau et à la clinique (subclinique). L'hygiène est aussi importante. C'est le germe le plus fréquent chez certains.
- Traitement : La thérapie est meilleure en début de lactation. Si on agit tout de suite on a plus de chance. Il faut faire une thérapie à la Pénicilline (+ Mamyzine (systémique)) pendant 5 jours. vet utilise Novomat et traite pendant 4 jours.
- Succès de la thérapie : On a l'impression que c'est bon cliniquement, mais il y a des récidives. Si la mammite devient chronique, ça devient très très difficile. Le succès est moyen (50%).

exp dit qu'on a meilleur temps d'utiliser de la Pénicilline que des Céphalosporines qui sont mieux adaptées pour les Gram-. Elle/Il confirme aussi que les piqûres de Mamyzine sont bien car celles-ci restent dans la mamelle. Cette méthode n'est pas très recommandée avec les tubes, mais dans les cas spéciaux, ça peut aider.

vet demande si il existerait un trempage efficace contre S.uberis. exp connaît quelque chose contre S.aureus, mais pas contre S.uberis. Ca peut aider de changer de produit. Il existe un produit à deux composantes qui est efficace mais qui ne peut pas tout le temps être utilisé, notamment à cause de la température. Il y a une discussion sur les produits qui déposent un film sur le trayon. Cela ne fonctionne que quand le trayon est propre.

vet utilise Kenostart (ersatz de Euravet) et dit que ce produit est importé en Suisse.

exp parle encore d'un produit de trempage avec des lactobacilles qui servirait à concurrencer les germes embêtants mais cela n'est vraiment pas efficace ! A éviter !

c. Autres streptocoques :

Groupe B :

- Anamnèse : Rien de particulier
- Traitement : Pénicilline pendant 24h.
- Succès de la thérapie : Bon

vet parle à nouveau de XYZ qui permet de différencier S.uberis et S.dysgalactiae.

La question se pose de savoir si il faut vraiment traiter S.dysgalactiae ? Apparemment pas forcément.

d. S.aureus :

Groupe A :

- Anamnèse : Mammite subclinique, troupeau, augmentation du nombre de cellule dans le tank
- Traitement : management (notamment de l'hygiène et de l'ordre de traite). On a deux possibilités de traitement : soit on traite, soit on réforme. Si on veut traiter, ils nous conseillent : Canamastine(6tubes / quartier)+ Pharmasine (33ml pendant 3 jours) 2X toutes les 12h + 2X toutes les 24h. Cela coûte très cher, mais le client est au courant et a choisi de traiter, donc il assume.
- Succès de la thérapie : pas mauvais selon vet, il obtient un 60% de succès chez les vaches traitées. Mais il fait toujours un contrôle bactériologique après la thérapie (20 jours) par PCR. 2X négatif = libre de S.aureus.

exp dit que pour augmenter les chances de réussite, il faut le faire soit pendant le tarissement, soit réformer la bête. Il est également important de tenir un registre afin de savoir qu'elle vache on veut garder et si oui quand la trait-on.

vet lui, demande un antibiogramme et traite non seulement en fonction du germe (Pénicilline si germe sensible / Traitement long avec Ubrolexine, Pharmazine si germe résistant), mais aussi en fonction de la vache. On ne traite pas de la même manière une jeune vache et une vieille vache... Le succès est estimé à 50%. Il a une exploitation qui est 100% positive à S.aureus et instaure un système de groupes de traite. Ca a l'air de fonctionner. Il a également été traire avec son client afin de voir les possibles erreurs.

De manière générale, un antibiogramme est effectué pour savoir de quel S.aureus on doit s'occuper. exp dit que le type B est le plus rapide, mais tous les S.aureus ont le potentiel d'être contagieux.

La question du vaccin est mise sur le tapis. vet le conseille mais seulement si la vache n'a pas encore vêlée. vet dit que cela permet de faire baisser les cellules, mais cela ne guérit pas l'animal.

Dans les exploitations qui possèdent un robot de traite, exp conseille le vaccin au début afin de diminuer la pression d'infection. Mais en général, on arrête tout assez vite.

e. SNC :

Groupe C :

- Anamnèse : Regarder l'hygiène de traite
- Traitement : Amoxicilline + Acide Clavulanique après antibiogramme
- Succès de la thérapie : Bon

vet ne traite que sur antibiogramme.

f. C.bovis :

Groupe B :

- Anamnèse : -
- Traitement : Pénicilline pendant 4 jours, mais seulement si l'animal présente une clinique.
- Succès de la thérapie : ?

exp ne traite jamais les mammites à C.bovis ou alors dans de très rares cas exceptionnels (avec une induration clinique, si c'est un problème de troupeau), mais dans tous les cas, elle/il ne traite que sur le court terme. La mammite peut durer 2-2,5 mois. Le suivi de troupeau est quelque chose d'important pour ce germe, l'hygiène aussi.

vet ne traite pas non plus et prend le temps d'expliquer pourquoi au client.

g. E.coli, Klebsiella :

Groupe A :

- Anamnèse : Il faut faire attention car la clinique peut fausser. En principe, clinique aiguë
- Traitement : vet propose un traitement intraveineux (Dexamethasone) et intra-mammaire (Gentapen). Ils trouvent dommage que Cloxacoli n'existe plus. On parle de Cobactan, vet trouve qu'il faut mieux tuer l'animal que d'utiliser ce produit.

-> vet traite ce genre de mammite avec Sulfonamide-Trimethoprim (Tandozine) et affirme que ça marche. exp utilise aussi cette méthode à la clinique.

-> vet propose un injectable (directement dans la mamelle) avec au moins 5 traites par jour, il faut vider le pis.

-> Si la vache a le pis très dur, vet propose d'utiliser du Marfloquin (+ Methiocalcine). Même si cela va à l'encontre des principes modernes, il trouve que cela fonctionne bien et que le succès est meilleur.

-> On évoque aussi Cobactan, Synulox, Mastiplan, Cloxa.

-> vet ne met rien en intra-mammaire

- Succès de la thérapie : 50% voire plus.

Si la vache est vraiment mal, vet propose de "Drencher" l'animal pour lui redonner un coup !

vet s'interroge de savoir si les Quinolone sont bien pour ce genre de mammite ? Il pense que non à cause de la libération des endotoxines lors de la mort bactérienne. exp trouve que c'est discutable, il faut en tout cas une bonne perfusion. Elle/Il recommande les Sulfonamides !

vet demande si Genta en IV et Genta en intramammaire pourrait être une solution. exp ne le recommande pas (il y a en plus des temps d'attente). vet l'a essayé une fois mais la vache y est restée.

h. Levures :

Groupe B :

- Anamnèse : changement de tube trop rapidement ?
- Traitement : autrefois, il utilisait Panolog mais maintenant ne sais pas quoi faire. C'est une mammite difficile, le mieux c'est de traire (management!) -> vet propose Mycolog
- Succès de la thérapie : ?

exp conseille de traire à outrance la vache et dit qu'il faut beaucoup de patience !!! Cela peut durer très long, même si en 2-3 semaine on arrive à faire baisser le taux de cellule, la mammite est encore là.

4. Parole à l'experte :

exp est contente des discussions et trouve que les participants ont eu un bon raisonnement. Ils réfléchissent juste ! Elle/Il admet/confirme/affirme aussi que :

- On peut parfois être en conflit avec les recommandations. C'est normal, et il faut "improviser en temps voulu".
- Pour les mammites chroniques / subcliniques, il ne faut pas utiliser de Pénicilline car Elle/Il ne se répartit pas correctement dans le pis.
- Il faut prolonger la thérapie pour les germes à problèmes. Si on choisi de traiter, il faut le faire comme il faut ! Donc assez longtemps.
- Il ne faut pas hésiter à se poser cette question : traiter ou réformer ?
- Le problème principal à l'heure actuel est S.uberis. Les symptômes peuvent être très très variables.

Récolte des opinions :

- vet : 9. Est très content, Ne met pas 10 car n'a pas trouvé la recette miracle contre S.uberis. A trouvé très intéressant de savoir les méthodes des autres, surtout pour les germes à problèmes
- vet : 9. Est contente d'avoir pu se situer.
- vet : 9. Est très content car comme il travaille seul, ça ne lui arrive pas souvent d'entendre les conseils/astuces d'autres vétérinaires. Cela lui permet de (ré)-ouvrir son esprit. Il compte sur vet pour la présentation de XYZ.
- vet : 9. Est content. Il explique qu'il a eu beaucoup de problèmes avec la bactériologie et que par conséquent, il fait peu d'antibiogramme et se base surtout sur l'expérience. Il demande l'avis de exp au sujet des ponctions de la tétine pour savoir le germe ? -> exp en a entendu parler mais ne sais pas encore trop quoi en penser. En tout cas, ce n'est pas encore dans les mœurs.
- vet : 7. Est content mais un peu déçu de ne pas avoir trouvé de solution miracle ...

– vet : 8. A déjà donné son opinion.

Protokoll Mastitis

Erfahrungen untereinander austauschen

2 Gruppen gebildet:

Innerhalb der Gruppen wurde die Anamnese, die Therapie sowie der Therapieerfolg von folgenden Keimen diskutiert. Danach wurden die „Ergebnisse“ in der gesamten Gruppe diskutiert

2. KNS

1. Anamnese:

VET Diese ist eigentlich bei allen Keimen gleich:

Fragen nach ZZ, vorheriger Mastitis, Laktationsstadium, wer hat Probe genommen, Viertelgemelksprobe oder 4/4 Probe (v.a. bei KNS)

> Therapie:

VET eher keine Therapie evtl. Penicillin oder über Trockenstehzeit

> Therapieerfolg:

VET evtl. Spontanheilung

EXP Es kann über 2-3 Monate gehen, bis ZZ wieder normal ist

VET Heilungserfolg schwer zu beurteilen, entweder ich ruf Landwirt deswegen nochmal an, oder beim nächsten Besuch wird danach gefragt

VET Wenn Bestandsproblem vorhanden war ist es für mich ein Erfolg, wenn es kein Bestandsproblem mehr gibt

VET Wie ist es mit Amoxiclav?

EXP 38% sind Penicillinresistent, Therapie mit .... sinnvoll

VET Macht systemische Therapie Sinn? Mit Tylosin?

EXP Ich habe noch nie systemisch behandelt. Es ist nicht grundlegend falsch, jedoch kommt man mit weniger auch zum Ziel.

VET Wenn mehr als 2 Viertel betroffen sind, ist es evtl. billiger

EXP Und Macrolide verteilen sich gut im Euter.

VET Diese werden auch wenig in der Humanmedizin eingesetzt. Finde das daher nicht schlecht.

EXP Trim/Sulfa geht auch gut ins Euter

VET Welche Wirkstoffe gehen denn gut ins Euter?

EXP Macrolide, Trim/Sulfa, ...,.........,..........,.............

Genta geht nicht gut ins Euter

VET Da stellt sich wieder die Frage, ob Blut/Euter Schranke geschlossen ist oder nicht

VET: Und wie gross ist überhaupt der Einfluss von AB bei Mastitiden?

Habe im Notfall einmal Landwirt am Telefon geraten Pen zu geben. In der Bakteriologie kamen Coli raus, jedoch ging es Kuh wieder gut.

VET Ich war bei der „XYZ Veranstaltung“ in Zürich. Die empfeheln bei akuter/perakuter Mastitis:

AB i.v., NSAID, Flüssigkeitstherapie

und dann bei gram - keine lokale Therapie und bei gram+ später noch lokale Therapie

VET: Eine Kuh die frisst und Fetzen in der Milch hat, die kommt eh gut

VET Für mich ist es klar Coli, wenn "Milch" Eiersuppenartig/wässrig ist

EXP lokal im Euter macht E.Coli viel LPS (Lipopolysaccharide), daher gibt es eine überschiessende Reaktion, NSAID machen daher Sinn

VET noch kurz eine Anmerkung zu akuter Mastitis: ich gebe immer 2 ml NSAID ins Euter

3. S. aureus GTB

1. Anamnese: s. oben
2. Therapie:

VET bei GTB: Merzen!

VET jedoch ist das auch immer wieder eine Diskussion mit dem Landwirt

VET stimmt, man muss von Stall zu Stall schauen. Bei einer jungen Kuh kann man es evtl. noch mit einer systemischen Therapie mit Mamyzin versuchen

Behandlung über 5 Tage, Therapie nach Antibiogramm, meist Amox/Clavulansre

VET Wie ist es mit Penicillin?

VET: Ich habe mit Ubrolexin z.T. Erfolg

VET Wie Viele behandelst du Homöopathisch ?

VET: Habe Nosodentherapie in einem Bestand mit S. aureus durchgeführt: 50% sind wieder gut geworden

VET Und allgemein bei chronischen? Wieviel behandelst du nur homöopathisch?

VET Die Mehrzahl der Tiere, ist jedoch nicht repräsentativ, da ich viele Bio Betriebe, oder Betriebe habe, die keine AB mehr einsetzen

4. C. bovis

1. Anamnese

VET: eigentlich ein Hautbesiedler, macht Euterreizungen, keine Mastitis

2. Therapie:

eigentlich nicht therapieren, evtl. Penicillin oder TS

VET: Melkhygiene und Dippen ist wichtig

VET: Wir haben in unserem Kundenstamm einen Bestand mit chronischer Mastitis und oft C. Bovis in der Kultur. Macht er evtl. trotzdem chronische Mastitiden?

EXP Das ist uns auch aufgefallen. Wir sammeln daher gerade C. Bovis Stämme. Evtl. gibt es eine Unterspezies, die Problem macht. Dass Kühe, die eine Begleiterkrankung haben, dann auch mit C. Bovis auffallen, hat sich nicht immer bestätigt.

VET Welche Behandlung ist sinnvoll? Penicillin?

EXP im Moment gibt es kein Standard-Protokoll für einen Resistenztest, wir wollen dieses mit den gesammelten Stämmen auch etablieren, es gibt 2 Stämme, die auf Penicillin weniger empfindlich sind, diese sind jedoch auf Aminoglykoside empfindlich

VET Das mit der minimalen Hemmkonzentration bei den Resistenztests empfinde ich im Allgemeinen als nicht gut. "Hat er noch erklärt warum?"

EXP Ich frage nochmal nach wo die Limiten sind. Bei Bakterizid oder Bakteriostatisch.

5. S. agalactiae

1. Anamnese:

   VET: sehen wir selten

2. Therapie: Penicillin

   VET: Wer sieht A. agalactiae bei chron. Mastitiden?

   EXP wir haben es einmal gesehen

   VET Welcher Streptococcus ist entscheidend

3. Therapieerfolg: gut

6. S.dysgalactiae

1. Anamnese: s. oben

2. Therapie:

   VET Differenzierungen sind oft Penicillin empfindlich, bei chronischem Stadium ist oft entscheidend wann therapiert wird

   VET Ist eine systemische Therapie sinnvoll?

   VET Wenn mehr als 1 Viertel betroffen ist, dann ja, ausserdem noch zusätzliche Klinik, wie dass das Euter geschwollen ist

   VET: Man sieht S.dysgalactiae eher selten, oder?

   VET Wenn man es einschickt, kommt es selten raus

   EXP Wir sehen es selten

   VET. Wir haben einen Betrieb der Probleme mit S. dysgalactiae hat

   EXP Wenn ein Betrieb Probleme mit S. dysgalactiae hat, ist es wahrscheinlich, dass er immer wieder damit zu kämpfen hat, die Melkreihenfolge ist wichtig, und während der Laktation behandeln

   VET. Bei der "…..-Veranstaltung" wurde eine Untersuchung vorgestellt bei der es deutliche Behandlungserfolge gab (Prozent?), S. aureus hatte schlechtere Behandlungserfolge (Prozent?), bei Streptokokkus war der Heilungserfolg mit Antibiotischer Behandlung bei 83%, bei KNS waren 81% der Behandlungen mit Antibiotika erfolgreich

7. S. uberis

1. Anamnese:

   VET akute/klinische Phase? Wurde schon mit Gentapen behandelt?

2. Therapie:

VET Wenn mit Gentapen behandelt wurde, umstellen auf Synulox. Das und Novomate benutze ich gerne.

VET Warum Synulox?

EXP 5% zeigen Resistenzen auch gegen Cloxacillin, jedoch gegen Amoxiclav empfindlich

VET Alleine wegen dem psychologischem Effekt auf Synulox umstellen. Das sind andere Tuben!

VET Wenn man von Monocillin auf Milliopen umstellt hat man auch schon diesen psychologischen Effekt.

VET: Exp hat doch damals erwähnt, dass S.uberis einen Biofilm macht.

VET Würde das für eine systemische Therapie sprechen?

VET: Ja. Bei einem Bestand habe ich alle Proben eingeschickt. Eine Kuh hatte S.uberis. Diese 4 Tage systemisch mit Mamyzin behandelt.

VET Mamyzin 10, 4 Tage hintereinander mit zuerst erhöhter Dosis? Warum? Penicillin ist doch Zeitabhängig?

VET Wegen dem therapeutischen Fenster, damit man schnell auf der hohen Dosis ist

EXP Man sollte beachten, dass die Kühe heute schwerer sind als damals als das Präparat zugelassen wurde.

VET Wie dosierst du, Exp?

EXP 3 Flaschen Mamyzin 10 über 5 Tage systemisch, ob systemische oder intramammäre Gabe spielt evtl. eine untergeordnete Rolle, bei der Behandlung von mehr als einem Viertel macht eine systemische Gabe von der finanziellen Seite aus evtl. mehr Sinn.

Bei chronischen Fällen sind die meistens nicht gut

VET Bei nur erhöhter Zellzahl? Spontanheilung

EXP Eine Behandlung mit Antibiotika ist empfehlenswert

VET Macht es Sinn über Trockenstehzeit zu therapieren?

EXP Das ist grundsätzlich besser. Alleine schon wegen dem hohen Wirkstoffspiegel im Euter und weil keine Milch im Euter ist

VET Hast du da keine Angst vor akuten Vierteln, die dann entstehen?

EXP Ich habe Kühe auch schon mal gar nicht behandelt. Bisher ist nichts passiert. Manchmal fallen die dann mit Flocken auf.

VET Ich hatte mal eine Akute 3. Grades mit S. uberis. Nach Nachfrage bei dem Landwirt hatte diese Kuh vorher schon S. uberis. Gibt es da vielleicht verschiedene Stämme?

EXP Es gibt Literatur die besagt, dass wenn viel S.uberis im Bestand ist und ein hoher Druck von aussen, "nistet" sich dieser im Euter ein, arrangieren sich unterschiedliche Stämme. Norwegen!

VET: Diese Diskussion gibt es seit Jahren

VET Das mit den Pathogenitätsfaktoren ist auch seit Jahren in der Diskussion. Da gibt es so viel Bewegung. Evtl. sollte man davon wegkommen.

EXP Nochmal zur parenteralen Behandlung: wenn systemisch behandelt wird, dann ein Präparat nehmen, dass sich gut im Euter verteilt. Das erste, was man sich überlegen muss: wie viele Viertel und wie lange schon?

In Holland sowie den USA gab es grosse Studien, die gezeigt haben, dass es keinen stat. Vorteil gibt, jedoch kann es gut sein, dass Einzeltiere darauf ansprechen es ist jedoch nicht der Standard

Also bei Einzelfällen, wenn Tier für den Besitzer wertvoll ist, S.uberis ohne Vorgeschichte, dann ja

Jedoch stark Besitzerabhängig

Bei gram pos. wirkt Penethamat, dies ist jedoch ein Reservewirkstoff

In der Schweiz und Norwegen wird Penethamat systemisch eingesetzt.

VET: Ich kenne einen Tierarzt, der in seiner ersten Praxis bei S. uberis keinen Erfolg bei der intramammären Therapie hatte, jedoch in der nächsten Praxis bei Mamyzin systemisch ja

EXP Es gibt definitiv Praxisunterschiede. Welche Fälle evaluieren sie wie? Wie viel machen LW selber?

VET: Erfahrung hat gezeigt, wenn ich hinter der Therapie stehe und überzeugt bin, dann funktioniert es besser

VET Wie ist das mit Salben zu therapieren. Bringt das was? Welche gibt es?

EXP Salbe ist immer gut.

- Eutrawest ad us. vet., Emulsion (Wirkstoffe: Campher - Eucalyptol - Guajacol - Methylsalicylat)
- Starke grüne Salbe ad us. vet. (Wirkstoffe: Campher - Methylsalicylat)
- Mammamint (Japanische Minze)
- Permammas® ad us. vet.[V], Emulsion (Campher - Guajacol - Menthol - Methylsalicylat) in der Schweiz nicht mehr zugelassen
- starke Rote??
- Weitere??

VET Es kommt nicht darauf an, welche man benutzt, oder?

VET Nützt das überhaupt, ausser der erhöhten Tierbeobachtung?

EXP Entzündungshemmer und Hyperämisierung helfen schon.

VET: Man kennt das doch selber. Manchmal tut es einfach gut. Man sollte die Kühe fragen können. Die Tierbeobachtung ist auch wichtig, ob es gut tut oder nicht.

VET Jedoch bringt es doch nur was, wenn ich es gut einreibe. Ein einfaches "Hinschmieren" bringt doch nichts.

EXP Man kann dem LW einen Plan geben. Dann ist er "beschäftigt".

VET Vor allen Dingen beschäftigt er sich dann mit den Tieren!

VET Bei KNS überbrückt man so die Zeit. Laut XYZ bringt ausmelken nämlich nichts.

EXP Bei einer Toxämie bringt das Ausmelken schon etwas. Jedoch setzt sich nicht jeder alle 2 Stunden unter die Kuh. Wir sollten uns von Standards, die auf alles passen verabschieden.

8. **Andere Streptokokken bzw. dann über Enterokokken diskutiert**

VET Welche sind damit gemeint. S. canis? S. equi?

EXP alle anderen ausser S. uberis, S. dysgalactiae, S. agalactiae

VET Sollen wir an Stelle der anderen Streptokokken nicht lieber die **Enterokokken** besprechen

EXP Gerne kein Problem!

Wie viele seht ihr so?

VET Sehr viel!

Diagnostik mit StrepTex?

VET In Schaffhausen ja, es gibt aber auch Enterokokken, die da nicht kommen, in der anderen Praxis machen wir Äskulin und CPS Agar. Das finde ich besser.

VET Ich diagnostiziere ihn zu wenig. Bei therapieresistenten Äskulin positiven ist es dann eindeutig

VET: ohne AB sehe ich auch gute Verläufe

Vet: Ich sehe es ab und zu, behandele mit Neo M und manche kommen gut

EXP Langwierige Behandlung mit hoher ZZ wie S.uberis

VET Wenn dann lange Therapie mit Penicillin

VET 20% der Enterokokken sprechen nicht an

EXP Ich habe ein schlechtes Gefühl bei der Heilungsrate, langwierig

VET Welche Wirkstoffe helfen?

EXP laut Antibiogramm sind sie Penicillin sensibel

VET Äskulin positiv und H_2O_2, mit Pen erwischt du sie (im Gegensatz zu KNS)

9. **Coliforme**

VET wir sind innerhalb der 3er Gruppe dann auf die akuten Fälle gekommen

- Anamnese:
  VET: wer hat die Probe genommen
- Therapie:

  VET Eher nicht behandeln, nur wenn nötig behandeln mit Gentapen. Ob behandeln oder nicht ist abhängig vom Landwirt
- Therapierfolg: kurz

Expertenrunde

i. Müssen chronische Mastitiden durch KNS bedingt therapiert werden?

wurde vorher schon beantwortet

ii. Chronische Mastitis: zusätzliche systemische Antibiose vorteilhaft oder nicht?

wurde vorher schon zur Genüge diskutiert

iii. Evidenz von Behandlungen

EXP Es gibt unglaublich viele verschiedene Studien mit verschiedenen Präparaten, eine Metaanalyse ist da sehr schwer

In Holland gab es eine Feldstudie mit verschiedenen Therapien über 3 Tage bei S.aureus und S. uberis (bei anderen war das nicht zielführend). Eine zusätzliche systemische Therapie zeigte dort keinen statistisch signifikanten Heilungserfolg. In Norwegen konnte gezeigt werden, dass Heilungschancen sinken bei chronischen Kühen, chronischer S.uberis wird mit NSAID behandelt und chronische Kühe werden vermehrt ausgemerzt

iv. Relevanz Bakteriologie (Erreger) -Ergebnisse in Bezug auf Therapie

grosse Relevanz, wurde durch vorherige Diskussion ja gezeigt

v. Antibiotikareduktion via ausmerzen von Problemtieren. Zusammenarbeit mit Milchverarbeitern

bei S. aureus klappt dort die Zusammenarbeit mit Betriebsleitern z.T. echt gut

VET: Mir hat mal ein Betriebsleiter den Tipp gegeben, dass es hilft, um die Melkreihenfolge zu beachten, den Tieren ein Halfter anzuziehen und die positiven dann festzubinden.

D. und VET: Das macht aber auch viel Arbeit!

vi. Ist eine intramammäre Behandlung bei G- Keimen sinnvoll?
Das wird Thema beim nächsten AK sein
VET Vorbehandlung mit Trockenstellern?
EXP Nein! Auch nicht 2 mal TS, Trockensteller und Orbeseal dagegen sinnvoll
VET: Bringt Orbeseal etwas?
EXP Ja!
VET: Wird es mehr gebraucht?
EXP Ja, vor allen Dingen wegen der Sensibilisierung
VET Bei Behandlung mit TS passt es nicht, Probe ist zu 1/3 von der Kuh und 2/3 vom Tank
VET Ich mache es s, wenn LW Probe bringt, dann bekommt es Trockensteller und Tübli, somit erziehe ich den LW, dass er Proben bringt

EXP wird vielleicht irgendwann nicht mehr nötig sein
Diskussion über die neuen Bio-Richtlinien zum Gebrauch von TS. Bei der Abgabe von TS ist nun jedes Mal ein Antibiogram nötig. Dabei ist es egal, wer das Antibiogram macht. Dies ist nur zum Abgrenzen von den konventionellen Betrieben gedacht. Exp schickt die neuen Richtlinien diesbezüglich rum

VET: Das ist ja nur eine politische Entscheidung, ohne medizinischen Hintergrund
VET Da gibt es zu viel Freiraum
EXP Das animiert um „irgendetwas“ in der Praxis zu machen
D: selektives Trockenstellen ist an sich gut. Aber für mich nur eine politische Entscheidung

Feedbackrunde

Positiv:¨

- Fachlich gut
- 2-3 Sachen kann ich heute mitnehmen
- Glacé ☺
- Angenehmer Abend
- Verschiedene Seiten /Therapien gehört
- Man macht es ähnlich, ich bin nicht so verkehrt ;)
- Infos über Resistenzen fand ich hilfreich
- S. uberis und S. aureus länger behandeln, das nehme ich vom heutigen Abend mit
- Diskussion angenehm
- Experte ist zu Wort gekommen, guter Experte
- Angeregte Diskussion mit jemanden auf Augenhöhe
- Moderation
- Gute Vorbereitung vom AK
- Fachlich: Wo stehe ich, ich muss nicht viel ändern und nehme trotzdem ein paar Tipps mit

  Negativ:

  Zu heiss

Protokoll Trockenstellen

Vet présente ses résultats

Tout d'abord, il regarde lesquels parmi ces clients sont intéressés par les tarisseurs. Il fait son étude sur le nombre de tarisseurs qu'il a vendu. Il veut une estimation des exploitants sur le nombre de tarisseurs antibiotiques utilisés. Il obtient un résultat, mais l'étude n'est pas tout à fait juste car les primipares faussent les statistiques. Il décide donc de tourner différemment son étude. Il recherche dans ses dossiers toutes les ventes d'Orbeseal qu'il a effectué auprès de ses clients depuis 2009. Il va comparer ces taux de vente de tarisseurs sans antibiotiques et de tarisseurs avec antibiotiques au fil des années pour voir le progrès obtenu. Le but étant de diminuer les tarisseurs antibiotiques.

Voici les résultats en bref :

– 2009 : Orbeseal 4% ; Orbenin 96%

– 2015 : Orbeseal 36,2% ; Orbenin 63,8%.

Il est donc très content du résultat même si cela met du temps, il voit le changement et le travail investi. La méthode utilisée est la communication aux éleveurs! A force de sensibiliser ses clients, il a réussi à avoir un impact sur ses ventes de tarisseurs.

=> Question de vet sans réponse : Que perd-il financièrement?

Réactions des participants sur l'étude de vet :

- vet dit que les éleveurs peuvent commander des tarisseurs bon marchés (3 euros pour 1000 vaches) en France. Cela pose un problème car il ne peut pas tout contrôler. "Les éleveurs ont 2 frigos!"
- vet et vet sont aussi concernés, ils connaissent des clients qui importent.
- vet : il demande si les éleveurs font aussi la combinaison (avec et sans antibiotiques)

-> vet répond que oui.

Chacun présente les tarisseurs qu'ils ont l'habitude d'utiliser. vet ne travaille qu'avec Orbeseal (exceptés les tarisseurs antibiotiques), les autres ont plusieurs tarisseurs différents dans leur stock.

Le délai d'attente est souvent le critère le plus important du point de vue de l'éleveur !

Réactions de exp sur cette discussion :

exp demande comment vet a fait pour valoriser Orbeseal.

vet répond :

– **Promotion au niveau du discours. Urgence de la problématique (il faut agir maintenant!)**

– C'est plus facile depuis que la loi d'Avril 2015 est en place. Les éleveurs semblent accepter plus facilement de changer depuis qu'il y a une loi sur le sujet...

– **L'important c'est d'avoir un bon discours et d'avoir une alternative à offrir.** Dire qu'il faut arrêter les antibiotiques ne suffit pas, il faut pouvoir proposer quelque chose d'autre (ici Orbeseal)

exp rappelle qu'une loi est déjà en vigueur depuis plusieurs années pour les exploitations bios ! Il ne faut pas l'oublier !

Rapport d'experte

experte présente son exploitation. C'est une exploitation bio, plutôt intensive pour du bio, il a beaucoup de bêtes en première lactation. Il a été suivi pendant 7 ans par le Tierspital de Bern pour son suivi de troupeau. Collaboration plus que positive car elle lui a permis de contrôler une grave infestation de S.aureus.

Les conseils d'experte pour un bon tarissement :

- 2 mois avant terme, échantillon laitier des 4 tétines -> envoi au laboratoire. Ce dernier lui transmet les résultats avec des conseils correspondant à son cas! Cela lui permet de savoir exactement comment traiter le cas échéant!
- Si le laboratoire lui dit de tarir avec des antibiotiques, il le fait, sinon il ne fait aucun traitement et laisse tarir naturellement. Il pense qu'Orbeseal est interdit en bio -> Affirmation à vérifier !
- Si il ne doit pas utiliser d'antibiotiques, il trempe les pis dans une solution de trempage tous les jours durant la première semaine du tarissement. Si il y pense, il fait de même la semaine avant le vêlage.

Les statistiques de l'exploitation d'experte :

Vaches taries en 2016 : -> 18 vaches taries dont 11 avec tarisseurs antibiotiques.

40% sans tarisseur antibiotiques

60% avec tarisseur antibiotiques

Réactions des participants :

De manière générale, les participants sont très intéressés par le témoignage d'experte.

vet fait les analyses lui-même à son cabinet mais trouve toujours des bactéries dedans. Quelle valeur limite faut-il utiliser ?

vet demande à experte combien de vaches ont fait des mammites lors du tarissement.

-> Réponse :aucune

vet reprend la question mais au sujet des mammites lors de la lactation suivante

-> Réponse : il y en a eu, mais cela dépend beaucoup de la vache. Toutes les vaches n'ont pas la même résistance. Mais experte dit que les vaches qui sont taries rapidement ont tendance à avoir plus de problème lors de la lactation suivante.

Mot de la fin: experte est à 100% favorable au tarissement sélectif!

Conseils d'experte sur le traitement de ses mammites et pour une mamelle en bonne santé :

La dernière vache qu'il a traité avait des S.aureus. Il a essayé avec 2 tarisseurs différents mais n'est pas du tout convaincu de cette méthode. Il ne la réutilisera plus. Il dit qu'avant (du temps de XYZ), il traitait beaucoup avec de l'homéopathie (mélange spécial de XYZ) et ça marchait pas trop mal. Ce qui était surtout bien c'était qu'il n'y avait pas de délais.

Pour des mamelles en bonne santé :

- Optimiser la machine à traire
- Entretenir la machine à traire. Et les tuyaux !!! -> changement des manchons (tous les 6 mois), utiliser des papiers et des produits spéciaux pour nettoyer.
- Vider le reste de la solution de trempage après chaque traite !
- Être strict avec les personnes qui aide à traire. C'est lui le chef, c'est lui qui décide ! Même si il n'a pas toujours raison. Trop de changement n'est pas une bonne chose.
- L'ordre de traite est très important ! Il permet d'éviter les contaminations. Il trait toujours en fonction du nombre de cellule de ses vaches.

Réactions des participants :

vet admire le fait que experte s'en sorte bien malgré sa petite production et le félicite. vet il dit que plus la production est grande, plus l'éleveur sera confronté aux problèmes.

experte

exp explique qu'il y a 2 indications pour l'utilisation des tarisseurs avec antibiotiques :

– Guérir les infections présentes

– Prévenir les problèmes -> maintenant c'est interdit sauf si l'exploitation est considérée comme étant une exploitation à problème.

Discussion des participants à ce sujet :

– vet dit que ce n'est pas idéal d'attendre les résultats pour mettre un tarisseur, car idéalement il faut le mettre au tout début du tarissement. Si les résultats sont tardifs, on perd de l'efficacité.

– vet dit que pour les petites productions comme experte c'est encore facile à gérer, mais que pour les grosses productions, cela demande un travail supplémentaire énorme.

-> exp est d'accord mais connaît quelques grosses exploitations qui s'en sortent très bien avec cette méthode.

Ce qui est important, c'est d'avoir un bon management du tarissement. Il faut le prévoir et non le subir!

– vet, vet et vet sont d'avis que le management est très important, mais qu'il n'est pas si évident de contrôler le tarissement

– vet "le vétérinaire ne peut pas faire le management des tarissement de ses clients", ça deviendrait très compliqué et il n'aurait pas le temps de le faire.

– vet trouve que cette nouvelle ordonnance donne plus de responsabilité au vétérinaire, Le vétérinaire est plus impliqué. Contrainte ou non ?

- Avantage : ressert les liens avec ses clients
- Désavantage : prend du temps et de l'énergie.

– vet dit qu'un bon tarissement ne se base pas seulement sur les tubes que l'on utilise, mais aussi sur l'affouragement de l'animal durant cette période. Il pense qu'il faudrait profiter de la problématique actuelle sur le tarissement pour sensibiliser les éleveurs sur le fait que l'affouragement est très important durant le tarissement.

La question qui revient toujours est : quelles sont les limites qui nous disent il faut utiliser les antibiotiques ou il ne faut pas les utiliser?

exp compare les différents pays en matière de critères nécessaires pour choisir si antibiotiques ou non. Les

autres participent aussi.

La Suisse est, avec un taux de cellules limite à 150'000, déjà bien au-dessous de la norme internationale qui est de 200'000 cellules. Cependant, aux Pays-Bas, les normes sont différentes : 50'000 cellules pour les primipares et 150'000 pour les multipares. **D'avis général, c'est bien de faire la différence entre primipares et multipares**. Ces chiffres néerlandais semblent être un bon compromis. Cependant, on ne tient pas en compte le nombre de germe qui a aussi son importance. Mais pour l'instant, cela donne déjà une idée de la marche à suivre.

– vet : dit qu'il utilise déjà ce système pour conseiller ses clients

Certes la Suisse a du lait de qualité car on conseille en traitement à partir de 150'000 cellules et que le lait trop contaminé n'est pas utilisé, mais la Suisse est le numéro 2 mondial de l'utilisation de tubes intramammaires!!! Tout cela remet en doute la discussion ci-dessus.

– vet : "enlever les antibiotiques pour le tarissement ? Oui mais a quel prix ?" -> pour les éleveurs, pour la qualité de lait, pour l'économie, ...

Alternatives au tarisseurs antibiotiques :

– vet dit qu'elle importait un traitement qui produit une diminution de la production laitière et qui aide au tarissement mais ce produit est retiré du marché. Sinon, elle/il utilise parfois Orotare (homéopathie). Ce produit qui diminue aussi la production laitière est aussi importé.

– exp a une autre alternative qui est très prometteuse : Tarital (plante), c'est un produit à donner dans la boisson lors du tarissement. Il favorise la production de bouchon et diminue la production de lait. Malheureusement, peu d'études ont été effectuées sur ce sujet. Mais elle/il précise que ce n'est pas LA solution miracle!

– vet conseille de prévenir les éleveurs des dangers pouvant apparaître avec certains produits "pas vraiment officialisés".

– vet pense que les éleveurs doivent faire plus d'effort pour optimiser leur tarissement et écouter les conseils des vétérinaires.

De manière générale, l'ordonnance pause problème. Elle/Il est arrivée très vite et les gens n'ont pas eu le temps de se préparer.

exp dit qu'en Suisse, le taux de cellule est l'indication pour la santé des mamelles et conseille de faire un CMT à partir de 150'000 cellules. Elle/Il dit aussi que les exploitations qui sont soumises au contrôle laitier ont un avantage sur les autres. Cela dit, il faut toujours traiter au cas par cas. Chaque exploitation est différente ! – vet ne sait toujours pas où se positionner au niveau de la quantité de bactéries. Il utilise des agar sélectifs (exp: je vais essayer). "Des bactéries il y en a, mais quand doit-on traiter?"

> -> exp répond : s'il y a une colonie bactérienne dans la culture, c'est déjà qu'il y a un problème. Ce qui est important, c'est de trouver à quel germe on a affaire. Ensuite, en fonction du germe on peut être plus ou moins souple. Au final, le tarisseur est là pour traiter les germes et non les cellules! Il faut donc s'aider des cellules pour trouver les vaches à problèmes et ensuite traiter en fonction du germe!

exp présente un peu son article (sur SVS) en parlant de la Norvège et des Pays-Bas notamment. Cela parle en faveur du tarissement sélectif!

– vet trouve que c'est une très bonne idée de prendre des échantillons avant de tarir, mais trouve que le prix des analyses bactériennes va à l'encontre du traitement sélectif car c'est trop cher !

> -> exp précise que c'est pourquoi on utilise le taux cellulaire. On peut ainsi sélectionner les vaches à analyser et diminuer les coûts.

Dans l'article de exp, il est dit que l'on se base sur les trois derniers contrôles laitiers pour estimer le taux de cellules et pour voir l'évolution de la santé de la mamelle chaque vache.

Les participants trouvent que c'est bien, mais il est assez facile pour les éleveurs de mentir sur les contrôles pour arriver à leurs fins et obtenir les antibiotiques qu'ils souhaitent. L'idéal serait que les éleveurs nous présentent les feuilles du contrôle laitier avant de pouvoir leur remettre des antibiotiques.

vet présente ses résultats

vet a distribué un questionnaire à 120 clients.

L'étude n'est pas tout à fait représentative pour la Suisse car elle/il fut faite au Valais avec des vaches d'Hérens et ces dernières ont un statut un peu particulier ; parfois, ce sont des vaches de loisirs.

Elle/Il a posé une question intéressante : pourquoi les éleveurs tarissent-ils ?

> -> réponse : pour la sécurité et la facilité.

Elle/Il explique un peu ce qu'ils ont mis en place dans son cabinet. Chez eux, ils se sont fixé pour objectif de faire les choses petits à petits. Par exemple : un client veut un tarisseur, je demande le nom de l'animal, la fois d'après je demande en plus le nombre de cellule, la fois d'après je demande autre chose. Le but ultime est que ses éleveurs arrivent chez je avec le contrôle laitier de la vache à tarir!

Elle/Il préconise la discussion avec les éleveurs!

Elle/Il parle aussi d'un vaccin (Starvac) qui pourrait permettre de réduite la pression d'infection. Le problème, c'estque ce produit ne fonctionne pas bien et qu'il coûte extrêmement cher!

– vet dit qu'il y a un nouveau produit sensé aider les vaches au début de la lactation

– exp dit que ça pourrait être bien de sélectionner les vaches résistantes, mais cette stratégie va durer des décennies.

tarir selon la loi

mod distribue les articles de loi pour que tout le monde puisse les lire. Elle/Il donne aussi une petite feuille pour que tout le monde puisse s'exprimer par écrit. Les affirmations de chacun seront discutées par après. Apparemment, il y a beaucoup à discuter.

Discussion sur la loi :

– vet : "la loi nous laisse beaucoup d'interprétation, on peut nous-même poser nos limites et faire notre loi".

– vet : "on ne risque rien juridiquement car le texte est trop flou pour être précis".

– vet : "effectivement, chaque vétérinaire fixe ses limites". Il dit qu'il va poser ses limites en fonction des recherches scientifiques, par exemple 50'000 cellules pour les primipares et 150'000 pour les multipares.

– vet met en garde que ce sont les cantons qui fixent les exigences. Il faut se renseigner avant de faire n'importe quoi. -> vet n'est pas d'accord (il travaille au Jura), les règles chez lui sont un peu différentes.

– vet dit qu'on peut traiter l'indication si ce n'est pas de la prophylaxie

– vet :" il faut de nouveau adapter ses méthodes selon l'exploitation. Mais pour sa propre sécurité, il faut toujours avoir des preuves écrites de ce que l'on fait. En fait c'est un peu chacun pour soit, le but étant de diminuer l'utilisation des tarisseurs".

exp leur distribue le plan de traitement qu'il a mis en place.

Ce n'est qu'une proposition, je ne dit pas que c'est la solution miracle mais pense que la méthode présente de bons avantages. Au moins, cela donne une idée à laquelle on peut (si on veut) se fier.

– vet résume en disant que chaque vétérinaire est libre de travailler selon ses méthodes et ses limites, la responsabilité lui revient. Mais le client est roi et il faut donc toujours s'adapter à ses clients si on veut qu'ils nous gardent. -> exp dit qu'il n'y a pas encore de solution contre les clients récalcitrants.

vet va suivre le schéma de exp et prendre ses libertés pour les clients récalcitrants.

– vet : ce qu'il va faire durant les 5 prochaines années ? : sensibiliser ses éleveurs et aller progressivement. Il va essayer de changer les mentalités.

– vet dit que le suivi de troupeau est quelque chose de très utile pour pouvoir parler à l'éleveur de ce sujet.

En résumé, il ne faut pas brusquer les choses et essayer d'avancer progressivement.

Malheureusement, aucune proposition de groupe n'a été faite. Mais il semble que chacun a une petite idée de sa propre stratégie.

exp clôt le débat sur ces mots : **il est important de discuter !**

Feedback

– vet : content d'avoir pu discuter de ce thème avec les autres

– vet : content, il avance à tâtons mais apprécie les avis des autres. Cela crée une dynamique.

– vet : apprécie l'exemple d'experte mais trouve qu'il tarit quand même beaucoup avec des antibiotiques. Elle/Il attend des alternatives

– vet : il va falloir beaucoup parler, manager les clients, cela va prendre du temps

– vet : à trouver la discussion intéressante. Il faut discuter de quel tarisseur pour quelle analyse.

– vet : mitigé. Apprécie les discussion mais n'a pas évolué, il ne sait pas vraiment quelle stratégie utiliser. Il va essayer d'identifier les mammites de tarissement et de voir ce que ces clients utilisent (-> nouvelle étude).

Protokoll Melktechnik

vet: Donne parfois des conseils à ses éleveurs mais est convaincu que discuter ne sert pas à grand chose, il faut plutôt aller voir comment travaille l'éleveur. Car même sans être un professionnel des machines à traire, il parvient tout de même à repérer des choses intéressantes durant la traite.

Vet : Il manque encore d'expérience, mais le fait d'aller voir renforce l'expérience. En allant plus souvent, il arrive mieux à se rendre compte des choses anormales et donc à mieux conseiller.

Vet ;Selon lui, l'important dans ce domaine se résume en trois points :

- Hygiène
- Machine bien réglée
- Technique.

exp:

Quand on va observer comment travaille le paysan, il faut idéalement seulement regarder et prendre note. Seulement à la fin, on explique nos observations à l'éleveur. Le fait de discuter durant la traite perturbe le travail de l'éleveur et peut gêner l'appréciation de notre visite !

vet: Il estime ne pas avoir assez d'expérience au niveau de la gestion de traite. Il a fait quelques visites mais ne peut pas conseiller dans tous les domaines car il a trop de lacunes et a l'impression de ne pas être assez compétent.

vet lui conseille de prendre le temps d'aller sur le terrain pour voir.

L'expert conseille aux vétérinaires de d'abord essayer d'aller voir durant la traite et que si il ne voit rien, il demande alors l'aide de quelqu'un de plus expérimenté.

vet: A un collègue dans son cabinet qui s'occupe de ce genre de cas. Lui a les connaissances de base dans le domaine mais ne peut que conseiller sur les généralités et non sur les aspects techniques.

exp: Ne sont ici relevées que quelques remarques, conseils, idées que exp a abordé durant la séance. Cette liste n'est malheureusement pas exhaustive car le nombre d'information reçu a largement dépassé ma capacité à écrire et retenir correctement les choses... Voici cependant sa carte de visite qu'il ne faut pas hésiter à utiliser en cas de besoin

- La **position de la griffe** est un point très important. Les manchons doivent être en position verticale -> Sinon, risque de traite à vide.

Le tuyau reliant la griffe aux canalisations ne doit JAMAIS traîner par terre, il doit toujours partir vers le haut. Cela cause des bouchons de lait qui génèrent un manque de vide. Le tout cause des irritations.

- L'**état des trayons** en dit long sur la performance de la machine à traire. Par exemple, quand l'orifice du trayon est "prolapsé", c'est souvent à cause d'un manque de pression.

La pulsation de la machine sert à permettre au sang de circuler dans les vaisseaux du trayon. La norme est de 46kP. Si la pression est trop basse, la fermeture se fait au-dessous le trayon ce qui fait que l'orifice du canal est tiré vers l'extérieur (jusqu'au prolapsus) par manque de massage sur le trayon (accumulation de sang). Les bouchons de lait dans les conduites peuvent être la cause d'un manque de pression.

exp: une fois que l'orifice du trayon est sorti, il est quasiment impossible de prélever un échantillon de lait stérile (contamination de l'échantillon).

- Les **autres problèmes sanitaires** (par exemple les problèmes d'onglons) sont des facteurs prédisposant pour une mauvaise santé du pis. L'animal ne peut pas combattre sur plusieurs fronts. Si les défenses sont dans le pied, elles ne peuvent pas être optimales dans le pis. Risque d'inflammation.

– L'**entrée d'air** lors de la mise en place de la griffe est une chose INTERDITE. Il y a un risque de transmission de gouttelette.

– La **stimulation** est également très importante. Le bruit de la machine ne suffit pas à stimuler ! Sans amouillage, la machine tire le lait de la citerne mais pas celui des alvéoles. Du coup, le temps que la descente du lait aie lieu, la griffe se décroche et le lait reste dedans ! Catastrophe !

L'amouillage automatique offert par certaines machines (DeLaval, Westfalia) est en général assez efficace.

– Concernant l'**hygiène**, l'expert conseille aux éleveurs qui ont "des crevasses où on pourrait planter des patates dedans" aux mains de porter des gants. Ces crevasses sont sources de contamination du trayon.

L'hygiène de l'écurie est également très importante.

exp: si l'éleveur doit laver les tétines avant de traire, c'est qu'il y a un problème hygiénique au niveau de l'écurie.

– Concernant le **nettoyage du trayon**, l'expert trouve que c'est une étape importante à condition qu'elle soit réalisée dans les règles de l'art. Il conseille d'utiliser soit un papier à usage unique sec, soit de la mousse. La laine de bois est plutôt déconseillée et le linge humide est carrément à bannir.

– Pour les **vaches 3 trayons**, il faut mettre un bouchon dans le manchon inutilisé et le nettoyer avant de le réutiliser.

– Concernant le **trempage post-traite** tout le monde trouve cela bien car cela offre une "multi-protection". L'expert conseille même de tremper les trayons des vaches taries à partir de 3 semaines ante partum (cela habitue la vache et évite les infections)

exp: les produits qu'elle recommanderait sont ceux contenant de l'iode et de la glycérine.

– L'**alimentation** et l'**abreuvage** sont deux points déterminants sur la qualité et la quantité de lait. Par 28°, une vache qui produit 30L boit environ 140L d'eau. Il faut s'assurer que les abreuvoirs fonctionnent. Au niveau de l'alimentation, il est particulièrement important d'éviter les mycotoxines.

– Concernant le **manomètre**, il doit être toujours visible quand on trait. Si il vacille sans arrêt, on "crée" des mammites (bouchons dans les conduites). Il doit rester stable quand la machine fonctionne et être sur 0 quand elle ne fonctionne pas.

– Lors de **changement de pièce** sur la machine, il faut garder un certain équilibre. Ainsi, si on doit changer un manchon, il faut les changer les 4, sinon cela va être déséquilibré et va causer

des problèmes lors de la traite.

Voici encore quelques questions qui ont été posées durant la séance.

Vet : Est-ce bien de mettre de l'Orbeseal aux génisses afin d'éviter les mammites d'été ?

Exp : L'avis général est plutôt pas.

Vet Et quand un génisse pisse le lait ?

Exp : Il faut la traire.

Vet Est-ce bien d'utiliser de la chaux pour les logettes ?

Exp La chaux sert à sécher. C'est bien mais il ne faut surtout pas en mettre trop car cela irrite la peau et il faut veiller à ce qu'il y ait toujours de la paille par dessus.

Exp Une bonne logette doit être sèche, plate (légère surélévation au niveau de la tête).

Exp : Il ne faut jamais désinfecter une logette !

Vet : Et qu'en est-il du composte ?

Exp est contre ! exp le déconseille également !

Vet Pourquoi certaines vaches perdent du lait 30 minutes après la traite ?

Exp C'est que quelque chose l'a dérangé durant la traite et qu'elle a retenu sont lait. C'est typique lorsque la vache est stressée car l'adrénaline est un frein pour la libération d'ocytocine. Voici quelques facteurs de stress chien qui court dans l'écurie, bruits inhabituels, portes qui claques ; courants vagabonds (rares).

Feedbackrunde :

vet: a trouvé cette rencontre très intéressante. On a vu trop de chose pour tout retenir, mais il pourra mieux se concentrer sur les aspects importants.

vet: trouve que c'était la réunion la plus intéressante depuis le début de ces cercles de travail. Du moins, c'est celle qui l'a le plus intéressé.

vet: est très content de la séance et est content de connaître maintenant quelqu'un a qui demander conseil.

Protokoll Bestandesbetreuung

Échanges d'expériences professionnelles

Chaque participant devait répondre à 4 questions concernant le suivi laitier. A savoir :

- Effectuez-vous le suivi de troupeau ? Et comment procédez-vous ?
- Facturez-vous les services de consultation à vos clients ?
- Avez-vous déjà vécu une expérience négative ? Laquelle ? Comment avez-vous réagit ?
- Utilisez-vous DSA ? Ou autre chose ? Comment et pour quoi utilisez-vous cela ?

vet :

Effectue un suivi avec le DSA avec certains de ses clients. Il télécharge les données nécessaires avant la visite et prend son ordinateur sur place pour effectuer le contrôle. Toutes les vaches laitières sont observées durant l'année.

Le service de consultation n'est pas facturé. Il facture seulement ce qui est planifié avec le client (p.ex : un rendez-vous pour effectuer un suivi de troupeau).

Les tarifs se font en fonction du travail effectué. 1 contrôle = 1 facture, 5 contrôle = 5 factures.

Il a vécu ne expérience négative avec des clients qui venaient de se mettre en association et chacun s'occupait d'une partie du cheptel. Il y avait un sérieux manque de communication entre les deux, ce qui rendait le travail difficile.

vet a appris a travailler avec DSA dès son premier travail. Il est bien familiarisé avec le système.

vet :Effectue le suivi depuis l'année passée, également avec le DSA. Elle/Il et son collègue n'ont pas encore d'expérience avec le système et cela rend le travail un peut difficile.

Elle/Il a vécu une expérience négative avec un client qui renouvelait fréquemment son cheptel, si bien qu'à chaque nouvelle visite, une partie des bêtes n'étaient plus là et quelques nouvelles étaient arrivées.

En …, les gens ont plutôt des petits troupeaux et par conséquent, connaissent bien leurs animaux. Le suivi de troupeau n'est donc pas souvent demandé.

Vet : marque chaque vache sur un calepin et reporte le tout le soir sur Oblon. Chaque vache est enregistrée dans Oblon.

vet facture la visite, les conseils ne sont pas facturés.

Durant la conversation, vet conseille d'arrêter d'utiliser des calepin et de directement insérer les données dans l'ordinateur portable. On perd beaucoup moins de temps et on ne fait qu'une fois le travail. Il peut ainsi étiqueter tout médicament sortant directement sur place et être toujours à jour avec son inventaire.

Vet : explique que ce n'est pas toujours facile d'utiliser le programme à l'extérieur du cabinet car il n'y a pas toujours le réseau nécessaire.

Vet : utilise également directement son ordinateur portable se trouvant directement sur le siège passager.

Vet : trouve que le programme coûte cher pour l'installer sur les différents ordinateurs du cabinet car il faut payer une licence par ordinateur.

vet :Effectue un peu de suivi de troupeau chez quelques clients notamment un gros clients qui a 120 vaches chez lequel il va toutes les 2 semaines. C'est le client qui fait le suivi et qui prévoit le travail à effectué lors de chaque visite.

Chez les autres, il va en principe 1x par mois et prépare le travail toujours 2-3 jours avant la visite afin que tout soit prêt.

Il facture le contrôles gynécologiques, et ceux de gestations et fait des rabais lorsqu'il y a beaucoup.

Il utilise le DSA pour quelques exploitations et facture 45.- le quart d'heure lorsqu'il utilise le programme.

Il n'a pas vraiment eu d'expérience négative. Certains clients se plaignent du prix mais c'est normal et vet a les arguments pour expliquer les tarifs.

Les tarifs sont environ de 180.- de l'heure. Les gestations sont facturées 12.-, les contrôles gynéco 22.5.- et il rabaisse à 20.- / contrôle si il y a plus de 5 contrôles.

vet :Effectue le suivi de troupeau avec une quinzaine de clients et des troupeaux allant de 15 à 150 têtes. Tous les lundis, quelqu'un du cabinet ne fait que ça.

Il facture 180.- de l'heure + les coûts de déplacement. Les actes ne sont pas facturés, les médicaments le sont. Les clients avec lesquels il fait le suivi ont le 10% sur les factures.

Il a comparé les prix entre le fait de payer à l'heure et le fait de payer à l'acte. Il faut favoriser le suivi de troupeau.

Il a vécu une expérience négative avec un client chez qui il faisait le suivi de troupeau. Le client s'est beaucoup plein car il n'y a pas eu d'amélioration dans son troupeau et un certain nombre de vaches étaient en déficit énergétique. En fin de compte, le problème ne venait pas du suivi de troupeau, mais du management alimentaire du client.

Il va au moins 12x par année faire le suivi chez ceux avec lesquels il a un contrat pour cela.

vet :

Effectue le suivi de troupeau depuis 3 ans grâce à XYZ (spécialiste en suivi de troupeau) et utilise le DSA (pour une fois qu'il existe un programme en français)

Il suit 8 exploitations en collaboration avec XYZ. Ce dernier fait les visites tous les 15 jours.

Il facture 196.- de l'heure du moment où il arrive sur l'exploitation, au moment où il s'en va. Cela offre l'avantage, que l'éleveur doit préparer les bêtes avant l'arrivée du vétérinaire car sinon il paye +.

Profite de l'expérience de XYZ pour corréler les conseils d'alimentation liés au éventuels problèmes de santé mammaire dans un troupeau. DSA est pratique pour analyser les problèmes alimentaires.

Il n'a pas vécu d'expérience négative.

Parole à l'expert

exp est l'expert du jour, il vient nous parler du programme DSA et de la nouvelle mise à jour avec les données pour la santé de la mamelle (DSA mammite).

Il explique certains truc et astuce pour pouvoir utiliser le programme de manière optimale. Je ne cite ici qu'une toute petite partie de ce qu'il a dit car il m'est difficile d'expliquer un programme que je ne connais pas. Mais exp a bien fait comprendre qu'en cas de question, il suffit de lui lancer un coup de fil et de regarder directement avec lui.

Voici quelques points importants :

- Il faut utiliser toujours les mêmes codes, termes, raccourcis afin de s'y retrouver facilement.
- Il faut entraîner les éleveurs à noter correctement les données concernant la santé de la mamelle, p.ex : en notant sur le calendrier des chaleurs les cas de mammites...
- **Entrer les bonnes données**
- **Prendre le temps de découvrir le programme**

Les avantages de DSA :

- Pratique pour effectuer un assainissement au sein d'un troupeau
- Avoir des preuves graphiques et statistiques pour étayer nos arguments lors du conseil à l'éleveur.
- Doit permettre de réduire l'utilisation des antibiotiques
- Permet de mieux visualiser la situation dans le cadre du tarissement sélectif -> il faut tout de même une bonne organisation, notamment en s'occupant du tarissement avant le jour où il faut tarir. C'est mieux de faire les analyses/contrôles 1-2 semaines avant la date de tarissement prévue.

– Sélecteurs qui donnent des alarmes pour rappeler le travail a effectué

Les désavantages de DSA :

– On ne peut pas encore utiliser les données directement depuis les logiciels (p.ex : Oblon), il faut donc introduire les données manuellement. -> Projet retardé à cause d'une initiative nationale qui n'aboutira peut-être jamais.

vet parle de ce programme et incite les vétérinaires à s'informer et à aller voter en novembre => …..a fait un résumé des ordonnances importantes.

Opinions personnelles

Vet: Les participants sont satisfaits de la séance.

Protokoll Diagnostik

Parole à l'experte.

En annexe se trouve la présentation de exp complétée avec les questions des participants et les réponses correspondantes (en commentaires), ainsi que les remarques de exp (en commentaires) et les précisions importantes de exp (en vert).

Mod : Situation actuelle de votre diagnostic.

Quand il a commencé de faire ses propres analyses, vet envoyait un double à un laboratoire afin de pouvoir comparer ses résultats. Comme c'était très souvent comparable, il a arrêté d'envoyer des doubles et fait ses analyses lui-même.

Il cherche en premier lieu a identifier le germe, et ensuite, il fait un antibiogramme si cela est nécessaire. Cela dépend du germe (il fait beaucoup d'antibiogramme pour les Staphylocoques) et de si il s'agit de mammites récidivantes.

vet a entendu qu'en agriculture bio, les éleveurs doivent obligatoirement faire un antibiogramme pour le tarissement. La question est de savoir si les laboratoires de cabinet peuvent effectuer ces antibiogrammes, ou plutôt de savoir si les antibiogrammes effectués dans un cabinet "amateur" peuvent être utilisés pour cette loi ? -> exp confirme par e-mail que les antibiogrammes "fait-maison" sont acceptés dans ces cas-là (annexe)

Échanges d'expériences professionnelles.

Mod : A quoi ressemble le laboratoire des participants ?

vet n'a pas de laboratoire pour le lait, collabore avec des laboratoires professionnels

vet utilise XYZ de chez XYZ ! Au début, il le faisait aussi à double en se comparant avec les résultats du laboratoire professionel. Il utilise cette méthode surtout le vendredi soir car les laboratoires sont fermés le week-end.

Concernant sa méthode, il trouve que c'est difficile à lire au début, il faut quelque temps avant de vraiment maîtriser la méthode. Maintenant, il lui faut 5 minutes pour faire l'analyse et 5 minutes pour interpréter les résultats. C'est pratique d'avoir un/e assistant/e car on gagne du temps !

vet n'a pas de laboratoire. Il a essayer une fois le XYZ mais a très vite arrêté !

vet n'a pas la possibilité de tenir un laboratoire dans son cabinet car il travaille tout seul et n'a

absolument pas le temps de faire des analyses bactériologiques. Il fait confiance aux laboratoires professionnels.

vet compte sur cette séance pour décider si oui ou non il va mettre en place un laboratoire bactériologique dans son cabinet ! Il attend avec impatience le "tuto de vet" et la réponse de exp sur les nouvelles régulations BIO.

vet utilise XYZ de chez XYZ Il a également un incubateur au cabinet. Il fait une démonstration de sa méthode.

Le "tuto de vet" :

Son laboratoire est composé de :

- Boîte de Pétri divisée en 3. Incubation entre 24 et 48h.
  - Blanc -> Mise en évidence des Gram négatifs
  - Jaune -> Mise en évidence des Staphylocoques
  - Rouge -> Mise en évidence des Streptocoques
- Boîtes de Pétri supplémentaires (Agar et McConley)
- Test d'agglutination pour la mise en évidence de Staph aureus (Pastorex Staph + / BioRad) .
  - Grâce à cela, on peut trouver tous les germes principaux.

Ensuite, il peut faire un antibiogramme en principe après 6h d'incubation. Il met 4 pastilles antibiotiques sur la culture recouverte de la colonie et observe la réaction pour voir si il y a des résistances. Ensuite, il mesure le diamètre autour de la pastille pour connaître les doses.

Au niveau du temps, cela lui prend environ 1 heure mais cela dépend du nombre d'analyses à faire.

Au niveau des coûts, il paye 5.- pour les boîtes + l'analyse + le temps de travail et facture cela 23.- la Bact et rajoute 10.- pour l'antibiogramme.

Il voit 2 inconvénients majeurs à sa méthode :

Puisqu'il peut faire ces analyses au cabinet, il peut également avoir les résultats le dimanche, ce qui fait que des fois, les jours de congés lui passent sous le nez

Cela prend tout de même un peu de temps -> Mais cela n'est pas un problème pour lui !

Feedbackrunde :

vet pose la question de savoir si la congélation est déconseillée pour les échantillons bactériologiques ?

exp et vet réponde que non, cela fonctionne très bien.

Le sujet intéresse vet surtout pour promouvoir l'analyse avant le traitement (quitte à mettre le lait au congélateur en attendant).

vet nous informe qu'il a invité ses clients à une soirée d'information pour les informer d'un projet pilote concernant le protocole de tarissement. A priori, le changement semble être accepté !

vet le met en garde, ça marche bien au début puis on oublie peu à peu... (ndlr : espérons que non...)

vet trouve la soirée intéressante, il a maintenant des pistes a explorer pour son cabinet.

vet est content de la soirée

vet demande des compléments au sujet des analyses dans les exploitations bios.

exp a répondu à cette question par e-mail (ajouté en pièce jointe)

Protokoll Mastitis

Erfahrungen untereinander austauschen

S. agalactiae

Anamnese

Kommt selten vor

Therapie

Neo-M-Salbe forte oder Miliopen

Ganze Euter behandeln (Blitztherapie)

Zum Trockenstellen mit Penicillin

Therapieerfolg

Gut

EXP Info: In Norwegen gibt es wieder mehr S. agalactiae, jedoch verwand mit humanen Stämmen (zoonotisches Potential), weniger strikt ans Euter adaptiert, auch im Mist, (Warteraum beim Roboter!) Keine klin. Symptome sondern erhöhte Zellzahlen
VET XYZ macht Studie, sammelt Stämme und zahlt dafür

Differenzierung von Streptokokken?
VET Schwierig, nehmen chromogenen Agar zur Hilfe aber es gehen manche Streptokokken bzw. Lactococcen unter. Spezifität ist nicht sehr hoch

S. uberis

Anamnese

Meistens chronisch, ab und zu akut, hohe Zellzahlen

Therapie

VET: Lange Behandlung, meistens eine Woche mit Penicillin (Monocillin, alle 24h) (oft gleichzeitig Mamyzin® 10 über 5 Tage) oder Amoxicillin/Clavulansäure (3 Injektoren nach 12 h dann alle 24h) auch evtl. mit Kombination Mamyzin (5 Tage)

EXP Standard nur intramammär 5 Tage, produktabhängig in welchem Abstand man dosiert, es gibt 1-2 Studien die keinen Mehrwert von systemischer Behandlung festgestellt haben. Jedoch von Kuh zu Kuh abhängig, es gibt auch welche, die sehr gut darauf ansprechen! Und man hat noch was in der Hinterhand.

VET Nur systemische Therapie, weil Penicillin schlechte Verteilung im Gewebe. Erfahrung gemacht, dass dann keine Streptokokken mehr nachweisbar, jedoch Hefemastitis oder andere Keime.

EXP Bei längerer intramammärer Behandlung ist die Gefahr etwas zu injizieren hoch!

Mamyzin : für Verteilung im Euter gemacht. Wahrscheinlich ähnlich von der Wirkung ob systemisch oder intramammär! Evtl. erwischt man S. uberis mit Mamyzin nicht so gut, wenn er sich einkapselt?

Evtl. S. uberis Stämme so gut an Euter adaptiert, dass Kuh es nicht stört, sie aber immer wieder hohe Zellzahlen bekommt und daher früher abgeht („esoterischer“ Gedanke)

VET Miliopen 5 Tage (schlechte Erfahrung, hat jedoch noch nie gute Erfahrungen mit einer Therapie gemacht)

VET Ubrolexin und Thylan spritzen (3 Tage), gibt es ab

Ubrolexin:

Studie von Böhringer: NSAID und Ubrolexin: Vet hat damit schlechte Erfolge gemacht

Vet und Vet setzen Ubrolexin auch nicht mehr ein

EXP Ubrolexin macht bei Streptococcen alleine von den Wirkstoffen her nicht viel Sinn (Streptococcen reagieren nicht gut auf Cephalosporin 1. Generation und auch nicht aufs Aminoglykosid)

VET zuerst Ubrolexin und Mamyzin eingesetzt, dann auf Miliopen gewechselt (mit Mamyzin), jedoch wegen schlechter Rückmeldungen wieder auf Ubrolexin mit Mamyzin

VET Miliopen zu wenig hoch dosiert, Monocillin gute Erfahrungen (3 Tuben alle 12h dann alle 24h) (evtl. mit Mamyzin, gibt es auch ab) bei Rezidiv Synulox

VET Penicillin und Mamyzin

Kanamastin:

VET EINMAL gemacht und dann gute Erfahrungen mit 4 Injektoren im Abstand von 12h

VET setzt es gelegentlich, wenn nichts anderes gewirkt hat ein, Erfolg lässt sich nicht beurteilen, eher bei Staph aureus als 2. oder 3. Variante

VET:Wie ist das mit alle 24 h applizieren, obwohl laut Präparat alle 12 h vorgegeben ist?

EXP Bei Penicillin Postantibiotischer Effekt (bei Cephalosporin und Aminoglykosid (Ubrolexin) ist alle 24h angegeben um postantibiotischen Effekt zu nutzen), alle 12h hat sich aus Melkrhythmus ergeben, erste 3 Tuben alle 12 und dann alle 24 h ist pragmatische Lösung, damit LW mitmacht bei der langen Dauer der Behandlung. Dieses ist wichtig! Wenn Bauer es mitmacht: alle 12 h applizieren!

Müsste man untersuchen wie weit Wirkstoffkonzentration absinkt.

Exp fragt bei XYZnach wie es mit dem postantibiotischen Effekt aussieht.

2 Tuben bei Ersttherapie auf einmal? (Bei Monocillin steht es auf der Packung drauf. Oder z.B. bei Gentapen)

EXP Wenn dosisabhängige AB dann mehr Erfolg

VET Bei akuten 2 Tuben Gentapen, bei chronischen nur eine Tube Gentapen

Therapieerfolg

VET Nicht so schlecht

VET recht guter Erfolg

VET recht guter Erfolg

Andere Streptokokken

Anamnese

Sieht man extrem selten

Therapie

Penicillin

Therapieerfolg

Gut

Strep. Canis: Beta hämolytisch, massive Zellzahlerhöhung

Exp: sehr selten, kommt von Hund/Katze; Menschen mögliche Vektoren

EXP noch nie gesehen

Dysgalactiae

VET viel von Swisslab nachgewiesen, Swisslab sagt: Umweltkeim, selten Behandlung

EXP intermediär nicht strikter Umweltkeim, jedoch noch nie ein Problem bei Behandlung

Enterococcen:

Exp: Äskulin-positiv, auf chromogenem Agar: ein wenig weisslich, nicht so viele Penicillin-Resistenzen, es sind 50% beschrieben, je von Land zu Land unterschiedlich

VET Therapie Synulox, jedoch keine guten Behandlungserfolge

EXP Anmerkung AK Romandie Merzung ist auch eine Therapie, gerade bei Tieren, die immer wieder Probleme machen

Kuh mit chronischen Streptococcen: ein Viertel trockenstellen?

EXP Kennt viele die das erfolgreich machen

VET Ein Tübli hoch und dann stehen lassen (?)

VET macht normales Penicillin und dann Orbeseal Tübli drauf. Nach 5 Tagen Milch untersuchen von anderen Vierteln > noch nie was gewesen

S. aureus

Anamnese

akutes Problem vs Bestandesproblem (viele hohe Zellzahlen, manche haben überhaupt keine Symptome)

Therapie

nach Antibiogramm und welcher Genotyp (Bestandesproblem?)

Bestandesproblem: Melkreihenfolge, Bauer muss mitmachen

Therapieerfolg

hängt vom Bauern ab

Einzeltier: Therapieerfolg z.T. gut, z.T. schlecht

EXP Publikation “Factors associated with bacteriological cure during lactation after therapy for subclinical mastitis caused by Staphylococcus aureus. Sol J[1], Sampimon OC, Snoep JJ, Schukken YH.“

Therapieerfolg höher bei jungen Kühe, keine Mastitis-Vorgeschichte, ein Viertel betroffen, frische Infektion, Ende der Laktation (gilt genauso bei S. uberis).Gerade bei jungen Kühen lohnt sich ein frühes Einschreite.

Bei chronischen, die evtl. in der letzten Laktation schon aufgefallen sind überlegen ob bei Trockenstellen therapieren oder evtl. merzen.

Bei akuten, toxischen muss man therapieren.

Bei multiresistenten S. aureus: erste Wahl: Merzen, 2. Wahl, das eine Tübli, was noch sensibel ist

Tylan oder Mamyzin als Alternative zum Penicillin

Ubrolexin auch (XYZ sagt, dass es gut funktioniert)

Vet: Bei EV ganzes Euter behandeln?

EXP Ganze Euterbehandlung (Metaphylaxe) ist vor Genotypisierung empfohlen worden. Wenn ein Viertel S. aureus positiv, dann gibt es eine erhöhte Gefahr für andere Viertel. Müsste vielleicht mal Studie gemacht werden, ob es da Unterschiede gibt.

Uni empfiehlt seit StAR nicht mehr alle Viertel zu behandeln. Beides ist vertretbar, jedoch mit schlechtem Gefühl bei der Behandlung allen Vierteln

VET Wenn mehr als ein Viertel betroffen ist, dann eher ganzes Euter behandeln?

EXP So könnten wir verbleiben. Jedoch vorher natürlich individuell entscheiden. Kuh für Kuh muss angeschaut werden und entschieden werden

>Zustimmung von allen Seiten

Vorher Trockenstellen auch eine Möglichkeit.

KNS

Anamnese

chronische Diagnose nach Milchprobe Antibiogramm

Therapie

Bei Frischgekalbten noch Abwarten mit Behandeln (mind. 6 Wochen), früher Trockenstellen

VET Kommt drauf an, ob Bauer Milch in Käserei abgibt und keine Zellzahlerhöhung duldet.

FALLS Therapie unbedingt nötig dann: Masticlav

Therapieerfolg

Gut

Prognose wenn man Abwartet vor der Behandlung: Wenn Kühe es von alleine schaffen, dass die Zellzahl runter geht, dann bleibt die Zellzahl unten

Vet: Ist eine Differenzierung sinnvoll?

EXP Quartett bzw. Quintett von Keimen, die besser ans Euter adaptiert sind:

S. chromogenes , S. haemolyticus, S. Simulans, S. Xylosus

Exp: differenziere ich gerne, weil ich die Betriebe kennt und es im Herdenmuster gut zu wissen ist. Für den Einzelfall ist es nicht nötig und beim Therapieschema und Resistenzmuster sind sie sehr ähnlich

C. bovis

Anamnese

höhere Zellzahlen

Vet freut sich, weil dann nichts Schlimmes in Probe drin ist.

Therapie

Wenn längeres Problem besteht: Neo-M- Salbe (dann wird die auch mal gebraucht ;))

EXP hat neulich ein paar Fälle gehabt, die nicht auf Pen reagiert haben

nach Antibiogramm: 2 Stämme mit verminderter Empfindlichkeit gegen Penicillin

falls man diese in der Praxis sieht, gerne einschicken

Therapieerfolg

gut

Kühe mit Schalmtest + oder ++ C. bovis kein Problem, sieht man am meisten

Jedoch gibt es ab und zu auch Kühe mit seltsamen Symptomen, chronischer Mastitis

(C. Bovis „wohnt“ im Strichkanal, evtl. ins Euter hochgewandert und verursacht da dann Probleme, auch von Immunstatus der Kuh abhängig)

E.coli, Klebsiellae spp

Anamnese

Akute, selten chronische Mastitiden

Therapie

Diskutiert wurde

- Drenchen
- Infusionen mit Glucose und Hypertoner Lösung und saufen lassen! (lauwarmes Wasser)
- Dauertropf auch eine Möglichkeit
- Parenteral Antibiose
- Intramammäre Antibiose

VET Fälle, die man Homöopathisch behandeln könnte, mit striktem (stündlichem) Ausmelken und guter Beobachtung,

Andere merken an: Jedoch nicht eine Kuh Tagelang rumliegen lassen > Tierschutzwidrig und min. ein Viertel kaputt. CAVE! Tierschutz!

Parenterale Antibiose Borgal (Sulfonamid- Trimethoprim) Wird nicht abgegeben!

EXP Sulfonamid- Trimethoprim, Tetracyclin, Fluorchinolon (nicht 1. Wahl) gehen gut ins Euter und bei akuter Mastitis ist Blut-Euterschranke eh offen

VET: Sepsis ist nicht so die Sache, sondern die Toxämie ist das Problem

VET gibt gerne systemische Antibiose, da ich denke, dass bei akuter Mastitis, wenn das Euter schnell anschwillt, die Verteilung mit Eutertübli nicht ausreicht

VET hat jahrelang nur Entzündungshemmer eingesetzt: gute Erfahrungen

Setzt jetzt bei akutem Viertel Gentamycin parenteral und Gentapen ins Euter, um den gleichen Wirkstoff zu haben

VET Pharmakologisch wohl nicht schlimm Sulfonamid und Penicillin gleichzeitig einzusetzen; funktionieren gleich, jedoch gibt es da unterschiedliche Meinungen

VET Antibiose parenteral (Pargenta) und intramammär (Gentamycin)

Standarttherapie bei akuten Vierteln ausser bei Coliverdacht (dann parenteral Baytril)

VET Marboxyl parenteral bei Verdacht auf Coli Mastitis, wenn kein Verdacht auf

Coli Mastitis, dann Gentamycin (jedoch bei Streptococcen nicht wirklich wirksam s. unten)

Diskussion, ob es Gentamycin wirklich braucht

VET Parenteral muss nicht unbedingt behandelt werden, es ist eine Toxämie!

Früher: Euter behandelt, gedrenscht und Metacam gespritzt

Jedoch mittlerweile „ängstlicher" nach Fallbericht der von XYZ korrigiert wurde, behandelt jetzt auch systemisch

Anmerkung AK Ostschweiz: Eine Kuh mit akutem Viertel „sein" lassen: Mutig sein ist schwierig. Kommt auf Kuh und auf Bauer an! Von Situation zu Situation entscheiden.

Volumentherapie ins Euter?

VET Nicht bei wasserlösliche Lösungen, das macht keinen Sinn

VET Bei Neo-M-Suspension ist es gut gegangen

Vet: Akutes Viertel: Gentapen: primär Behandlung bei Streptococcen? Gibt es einen Nachteil?

VET Kollege hat es eingesetzt in akuten Viertel, aber Vet ist zurück auf Sulfonamid-Trimethoprim

EXP Gentamycin Resistenzen von E.coli: evtl. bei 25 %? Exp: schaut nochmal nach

VET Aminoglykosid und Penicillin synergistisch bei S. uberis: es sollte funktionieren

VET Gentapen: 3-mal nacheinander und 2-mal im Abstand von 24h . Wenn Bauer sagt, dass es nichts bringt oder es gibt ein Rezidiv, dann wird Behandlung für Streptokokken angefangen

Amxicillin Clavulansre bei Coli?

Viele haben die Erfahrung, dass das nicht gut war.

VET Cobactan bei Coli hat gut funktioniert (2. Wahl)

VET setzt Masticlav (Amxicillin, Clavulansre, Prednisolon) bei Coli ein, das funktioniert gut

Diskussion, ob evtl. wegen Prednisolon?

Anmerkung bei Toxämie Glaubersalz bei akuter Mastitis (Seminar XYZ)?

Es war noch niemand so mutig!

Therapieerfolg

wenn man sie erwischt, sind sie dankbar

Hefenmastitis

Anamnese

Medikamente direkt aus der Flasche ins Euter geben

Langezeitbehandlung

Therapie

> häufiges Ausmelken
> NSAID > wiederspricht wohl Homöopathie weil Fieber ja gut ist

Therapieerfolg

braucht lange, aber dann gut

Expertenrunde

Alle Fragen, die damals auf Karteikarten geschrieben wurden, sind beantwortet worden!

Feedback

Therapie war spannend
Wichtige Überlegungen wurden gemacht: Toxämie und Sepsis unterscheiden!
Erwartungen wurden erfüllt
Gut gewesen, gute Anregungen bekommen, fühlt sich bestätigt
Viel geändert in den letzten Jahren bei Therapien
Therapie spannend, jeder hat andere Erfahrungen!
Gut gewesen
Sehr interessant, viele haben gleiche Probleme und ähnliche Behandlung
Reserveantibiotikaeinsatz machen manche eher restriktiv, manche schon strenger, man kann was mitnehmen evtl. an diesen ein wenig anpassen, aber auch sehen, dass man nicht völlig daneben liegt
Gut zu sehen, wo man steht, Therapie und Diagnostik haben sich in den letzten 25 Jahre geändert, in Zukunft wird es sich weiter ändern!

FGD

Was hat dir diese AK gebracht im Hinblick auf Therapie?

Bestätigung eigener Therapie durch Austausch mit anderen

Kleine Anpassungen, nicht generelle Wechsel

Diskussion über Einsatz Reserveantibiotika

Wissen über Supplemente

Keine Wunderlösung bei S. Uberis

Behandlung und Dauer

Teilen von Erfahrungen Orbeseal

Prophylaxe? (TS)

Bestätigung

CNS behandeln/nicht behandeln

Gram neg

Keine IMM Behandlung

Was hat dir dieser AK gebracht im Hinblick auf Diagnostik?

Ansatzpunkte für Praxisdiagnostik

Neue Methoden

Praxislabor

>AB-Gramm

>Schnelligkeit der Resultate

Vertrauen in Resultate

>Austausch

Genauigkeit/Gründlichkeit/Strenge

Ring-Trial

>Benefit Labor

>Limiten Praxis

>Vielfalt

>Vergleich

>Neg zeitliche Begrenzung vom ZOBA

CNS Typisierung

Was hat dir dieser AK gebracht im Hinblick auf dein Verhältnis zum Kunden

Gefühl: mit mehr Überzeugung therapieren

Rückblick (Kultur)

Sehr kompliziert

Kosten

Austausch von Erfahrungen

Interesse an Bildung veterinär

Glaubwürdigkeit

Sichtbares Engagement

Rückenstärkung (kritische Kunden)

Teamstärkung/Mitarbeiter anleiten

Gespräch mit Landwirt

TS Entscheidungskriterien

LW fühlt sich ernst genommen

Verstärkte Glaubhaftigkeit

In welcher Hinsicht hat sich dein AB-Verbrauch geändert?

Unterstützungstherapie fördern

NSAID empfehlen bei hoher ZZ aber ohne Wachstum in Kultur

z.B. Drenchen

Rückschritt durch Orbeseal Lieferproblem, was den AB-Einsatz anbelangt (Abhängigkeit durch äussere Faktoren)

Praxis war schon sehr fortschrittlich

Therapiedauer

TS: weniger Orbenin, mehr Orbeseal

Striktes Handeln bei Anfrage für Analyse

Limitierung der Lieferun

Analyse für kritische AB

Reduktion im Gebrauch

Nichts geändert

Noch immer ohne Analyse

Bewussterer/gezielterer Einsatz

Reduktion AB TS

Betriebliche Unterschiede

Nichts

Bestätigung

Würdest du nochmal an einem AK teilnehmen?

Ja

Ja

Austausch

Interessant

Motivation

Generationsübergreifend

Vergleichend

Persönliche Entwicklung

Ja
