# Supplementary material for: Veterinary peer study groups as a method of continuous education—A new approach to identify and address factors associated with antimicrobial prescribing
Source: PLoS One. 2019 Sep 19;14(9):e0222497. doi: 10.1371/journal.pone.0222497 (PMC6752762; doi:10.1371/journal.pone.0222497)

S1 Fig: Code-co-occurence-model of the 35 codes summarized into 9 categories. Each category is identified by a different symbol.

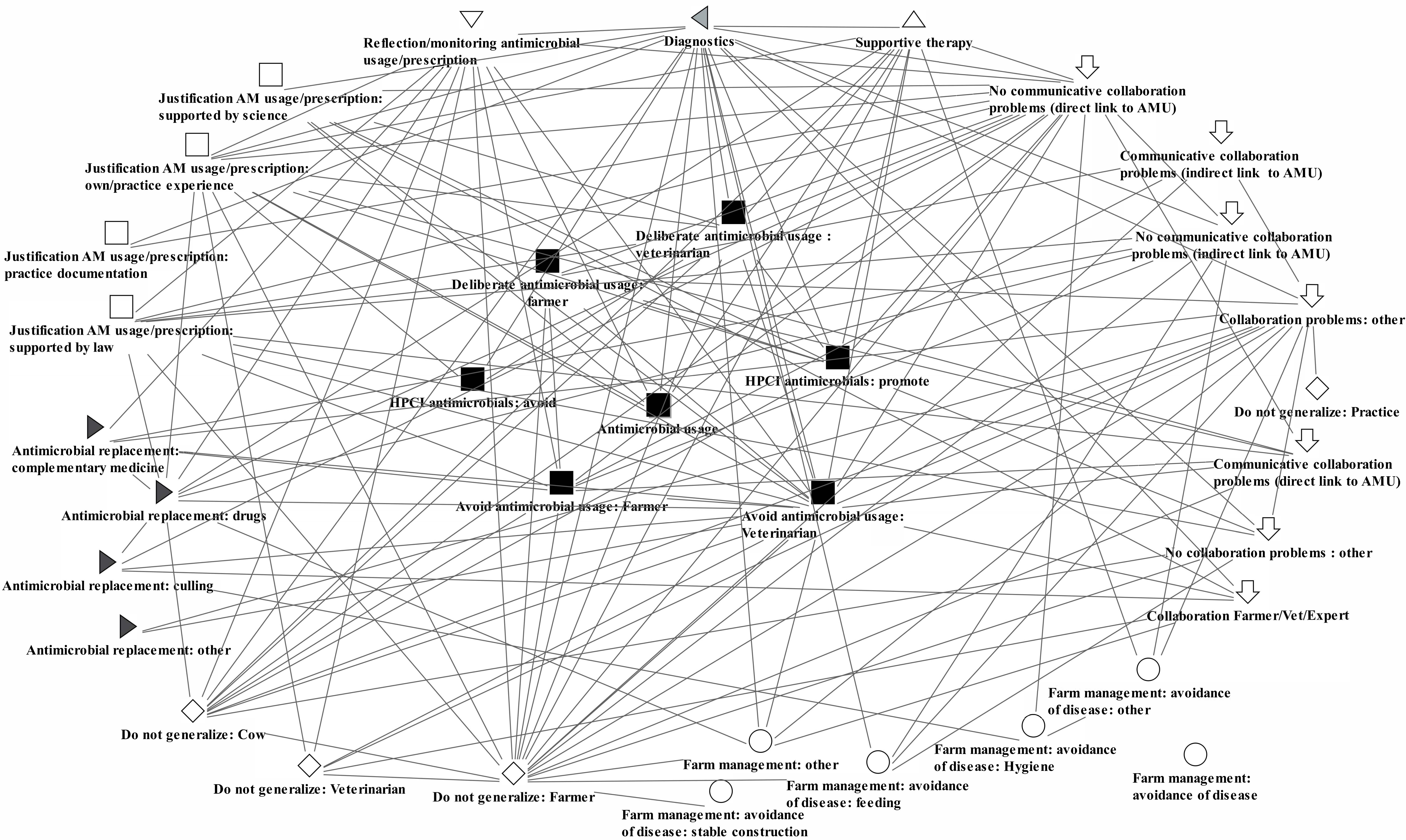

Supplement: S1 Fig — (PDF) [file pone.0222497.s003.pdf]
