# Supplementary material for: Veterinary peer study groups as a method of continuous education—A new approach to identify and address factors associated with antimicrobial prescribing
Source: PLoS One. 2019 Sep 19;14(9):e0222497. doi: 10.1371/journal.pone.0222497 (PMC6752762; doi:10.1371/journal.pone.0222497)
Supplement: S1 Table — (DOCX) [file pone.0222497.s004.docx]

**S1 Table: Number of meetings, topics and participation per VPSG.**

|  | VPSG Berne | VPSG Eastern Switzerland | VPSG Romandie | Total |
| --- | --- | --- | --- | --- |
| Number of meetings^1^ | 8 | 8 | 7 | 23 |
| Number of discussed topics | 7 | 6 | 6 | 19 |
| Number of participants | 8 | 8 | 7 | 23 |
| Mean number of participants for every meeting | 7 | 7 | 4.83 |  |

^1^Number of meetings including the initial kick-off meeting

VPSG: Veterinary peer study group
